# Improved subjective sleep quality in older adults by enhancing the GABAergic system in the sensorimotor cortex

**DOI:** 10.1113/JP290164

**Published:** 2026-07-05

**Authors:** Selin Scherrer, Sven Egger, Xinyu Liu, Anna Zoé Wick, Björn Rasch, Lijing Xin, Benedikt Lauber, Wolfgang Taube

**Affiliations:** ^1^ Department of Neurosciences and Movement Science University of Fribourg Fribourg Switzerland; ^2^ Laboratory for functional and metabolic imaging (LIFMET) École Polytechnique Fédérale de Lausanne Lausanne Switzerland; ^3^ CIBM Centre for Biomedical Imaging Lausanne Switzerland; ^4^ Animal Imaging and Technology École Polytechnique Fédérale de Lausanne (EPFL) Lausanne Switzerland; ^5^ Department of Psychology University of Fribourg Fribourg Switzerland; ^6^ Institute of Physics École Polytechnique Fédérale de Lausanne (EPFL) Lausanne Switzerland

**Keywords:** balance, exercise intervention, functional connectivity, GABAergic inhibition, healthy ageing, sleep

## Abstract

**Abstract:**

Sleep disturbances affect nearly half of all adults aged 60 and above, reducing quality of life and contributing to various health issues. While pharmacological treatments enhancing GABAergic activity can improve sleep, they often have adverse side effects. Given that balance training has been shown to enhance GABAergic inhibition in the sensorimotor cortex, we hypothesized that it could serve as a non‐pharmacological intervention to improve sleep quality in older adults. In this study, 36 adults aged 64 to 81 years either completed a three‐month balance training programme or served as controls. Before and after the three months, all participants underwent behavioural and neurophysiological measurements. Following a significant group × time interaction with repeated‐measures ANOVA, paired *t* tests revealed that the balance group experienced improved sleep quality assessed with the Pittsburgh Sleep Quality Index questionnaire. However, objective sleep parameters assessed with polysomnography remained unchanged. Neuroimaging revealed increased GABA levels in the sensorimotor cortex (magnetic resonance spectroscopy) and enhanced sensorimotor functional connectivity (functional magnetic resonance imaging). Although intracortical inhibition (transcranial magnetic stimulation) during balancing and sleep did not show significant group × time interactions, individuals with greater increases in inhibition during sleep reported larger improvements in sleep quality. Regression analyses indicated that greater increases in functional connectivity were associated with larger improvements in subjective sleep quality, whereas greater increases in GABA levels were associated with smaller improvements. These findings suggest that balance training improves subjective sleep quality in older adults, possibly by restoring the GABAergic system in the sensorimotor cortex, offering an accessible and non‐pharmacological intervention for age‐related sleep disturbances.

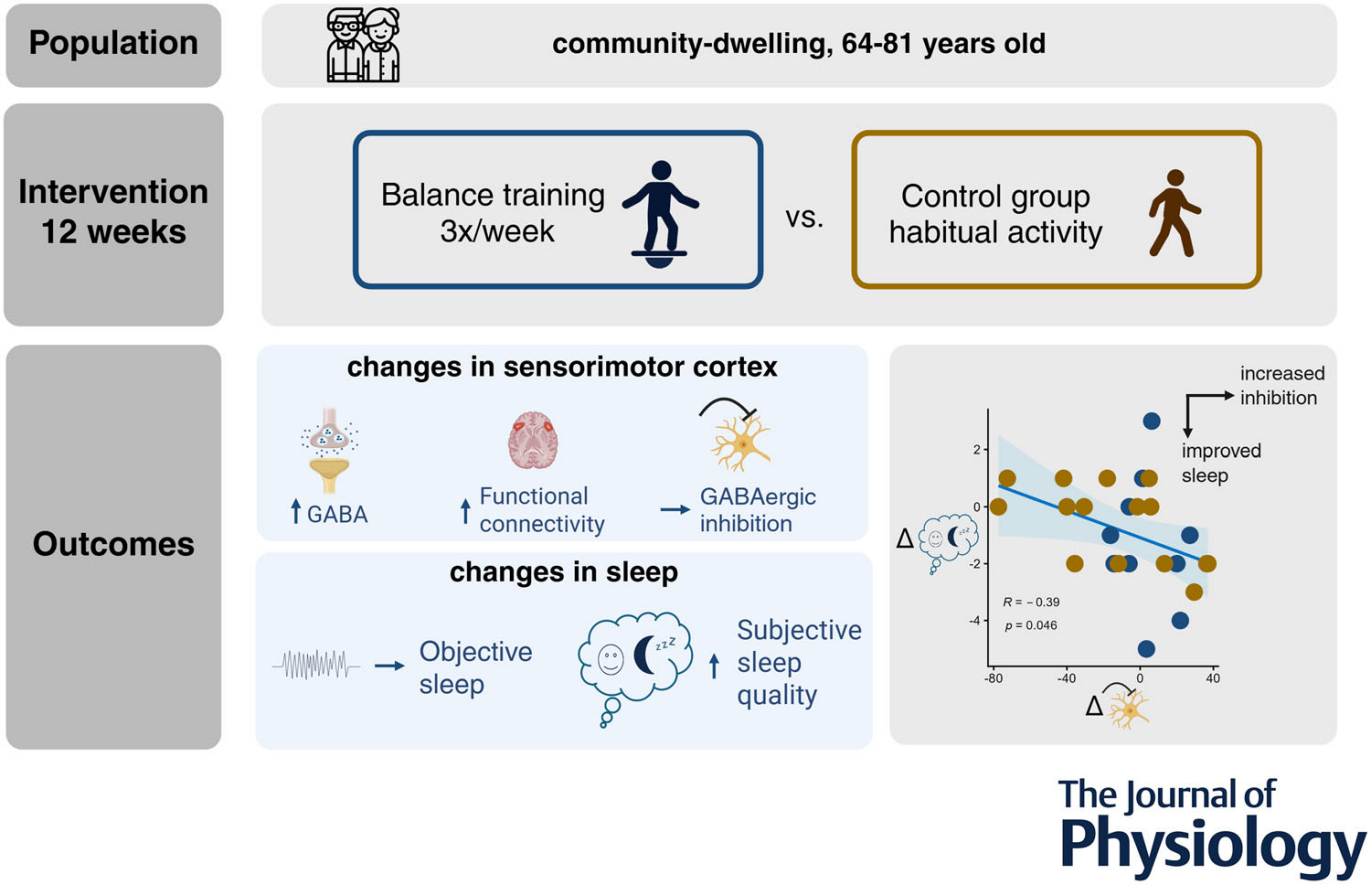

**Key points:**

- Sleep disturbances are common in adults over 60, and medications that enhance GABAergic inhibition often cause unwanted side effects.
- Previous research suggests that balance training can strengthen GABAergic inhibition in the sensorimotor cortex.
- In this study, three months of balance training improved subjective sleep quality in older adults, while no changes were observed in objective sleep measures.
- These improvements may be driven by enhanced GABAergic inhibition in the sensorimotor cortex, as reflected by increased functional connectivity and intracortical inhibition, two functional markers of GABAergic activity.
- These findings highlight balance training as an accessible and multi‐beneficial intervention to promote sleep health in older adults.

## Graphical abstract

**Abstract figure legend** Sleep disturbances are common in older adults, highlighting the need for accessible interventions to improve sleep quality. Eighteen older adults per group (balance training and control) were assessed before and after 12 weeks. The balance group increased GABA levels (magnetic resonance spectroscopy) and functional connectivity (functional magnetic resonance imaging) in the sensorimotor cortex and reported improved subjective sleep quality (Pittsburgh Sleep Quality Index). Changes in GABAergic inhibition (short‐interval intracortical inhibition) during sleep were positively associated with changes in subjective sleep quality. Balance training improved subjective sleep quality in healthy older adults, possibly by improving the functional use of GABAergic inhibition. Created in BioRender (https://BioRender.com/qwikfu3).

## Introduction

Sleep difficulties are a common concern among older adults, affecting nearly half of the individuals over the age of 60 (Canever et al., 2024). Poor sleep quality is associated with numerous adverse health outcomes, including an augmented risk of depression, cardiovascular disease and premature mortality (Del Brutto et al., 2024; Hoevenaar‐Blom et al., 2011; O'Leary et al., 2017). Sleep quality can be assessed using both subjective and objective methods. The gold standard for objective sleep assessment is polysomnography (PSG), which records multiple physiological parameters such as brain and muscle activity to determine time spent awake and in the different sleep stages, giving an overview of the sleep architecture (Iber et al., 2007). Subjective sleep quality is commonly measured using the clinically relevant Pittsburgh Sleep Quality Index (PSQI; Buysse et al., 1989). Notably, subjective sleep quality has been found to have a great impact on quality of life and is a stronger predictor of global cognitive functioning than objective sleep measures (Kudrnacova & Kudrnac, 2023; Lin et al., 2024). Moreover, subjective complaints of poor sleep are central to diagnosing insomnia, a frequent sleep disorder in older adults (Schneider et al., 2025). These considerations highlight the need for effective and accessible interventions to improve subjective sleep quality in ageing populations.

Primary pharmacological treatments against sleep disturbances target the γ‐aminobutyric acid (GABA) system, enhancing GABA_A_‐receptor activity to suppress wakefulness and promote sleep (Oishi et al., 2023; Patel et al., 2018; Saper & Fuller, 2017). GABA is an important player in sleep regulation and the main inhibitory neurotransmitter in the central nervous system. In a recent review, Taube & Lauber (2024) proposed a two‐layer model of cortical GABAergic inhibition: The first layer, referring to cortical GABA levels, reflects the brain's overall inhibitory capacity, which can be assessed by magnetic resonance spectroscopy (MRS). The second layer represents the ability to use the available GABA neurotransmitters and modulate GABAergic inhibition in a functionally relevant manner. This can be evaluated in the motor cortex by measuring short‐interval intracortical inhibition (SICI) via transcranial magnetic stimulation (TMS). However, it remains unclear how intervention‐induced increases in inhibitory capacity (GABA levels) and functional use of inhibition (SICI) relate to changes in subjective sleep quality in older adults.

Both layers of GABAergic inhibition have been shown to decline with age (Gao et al., 2013; Hermans et al., 2018; Marneweck et al., 2011; Papegaaij et al., 2014; Porges et al., 2021) and this age‐related decline makes the GABAergic system a particularly noteworthy target in older adults experiencing sleep problems. However, current pharmacological treatments targeting the GABAergic system are associated with adverse effects, including cognitive impairment, loss of motor coordination and increased fall risk (Berry et al., 2013). This highlights the need for safer, non‐pharmacological alternatives treating poor sleep quality in older adults.

Sleep regulation involves inhibitory mechanisms distributed across multiple brain regions. While earlier research has predominantly focused on subcortical GABAergic mechanisms (Oishi et al., 2023), growing evidence supports a complementary role for cortical top‐down processes in homeostatic sleep regulation and highlights the importance of motor suppression during sleep (Krone et al., 2017; Liu & Dan, 2019). Supporting the involvement of the sensorimotor cortex, a subset of sleep‐active GABAergic interneurons found in the motor cortex has been linked to deeper and more continuous sleep in rodents (Gerashchenko et al., 2008; Morairty et al., 2013). Similarly, studies in humans have demonstrated increased intracortical inhibition (i.e. SICI) in the primary motor cortex during sleep compared with wakefulness (Avesani et al., 2008; Salih et al., 2005). Furthermore, recent brain connectivity research reveals a dynamic interplay among various brain networks across sleep–wake stages, with sensorimotor regions showing more persistent activity during sleep than during wakefulness (Tarun et al., 2021). Clinical findings further underscore the relevance of the sensorimotor cortex in sleep regulation. For instance, impaired sensorimotor cortex activation patterns have been observed in insomnia patients following stroke and decreased functional connectivity between the left and right sensorimotor cortices has been reported in middle‐aged and older adults with insomnia (Chen et al., 2024; Wang et al., 2022). Together, these findings suggest that the sensorimotor network may represent a promising target for sleep enhancement, particularly through movement‐based interventions.

Until recently, there was a lack of longitudinal evidence demonstrating that non‐pharmacological interventions could enhance GABAergic inhibition in humans. But recent studies have shown that several weeks of balance training elevate GABA levels in the sensorimotor cortex (i.e. capacity for inhibition) and increase SICI (i.e. the ability to modulate inhibition) in both young and older adults (Kuhn et al., 2024; Liu et al., 2024; Mouthon & Taube, 2019; Taube et al., 2020). These findings suggest that balance training enhances both the capacity for inhibition (i.e. GABA levels) and the functional use of the available GABA (i.e. SICI). It has been assumed that the increase in inhibition may be an important factor to reduce age‐related cortical overactivation, a phenomenon characterized by increased and less distinct brain activity during motor and cognitive tasks (Kuhn et al., 2024; Reuter‐Lorenz & Cappell, 2008; Taube & Lauber, 2025). This overactivation may impair the suppression of unwanted movements and negatively affect sleep quality. Taken together, the enhancement of the GABAergic system and the reduction of cortical overactivation suggest that balance training has the potential to serve as an effective, exercise‐based intervention for improving sleep quality.

Interestingly, GABA regulates not only activity within the sensorimotor cortex but also communication between brain hemispheres, as GABA has been linked to functional connectivity strength within the resting‐state sensorimotor network (Blanco‐Hinojo et al., 2021; Fingelkurts et al., 2004; Stagg et al., 2014). For example, Blanco‐Hinojo and colleagues (Blanco‐Hinojo et al., 2021) demonstrated that enhancing GABA_A_‐receptor responsiveness with a benzodiazepine increased functional coupling between the left and right sensorimotor cortices. They suggest that GABA plays a key role in synchronizing (bilateral) brain areas involved in coordinated motor (in)activity. Within‐network functional connectivity also declines with age, contributing to neural dedifferentiation (i.e. reduced neural selectivity) and diminished sensorimotor performance (Cassady et al., 2019; Chan et al., 2014; Huang et al., 2015). Similarly, insomnia patients show reduced functional connectivity between bilateral sensorimotor cortices compared with healthy controls (Chen et al., 2024). However, recent evidence suggests that non‐pharmacological interventions such as balance training and Tai Chi, an intervention that includes balance exercises, restore functional connectivity within this network (Cerna et al., 2024; Chen et al., 2024; Liu et al., 2024). These findings suggest that balance training‐induced enhancements in the GABAergic system may also help to counteract age‐related connectivity decline to improve sleep quality.

In this study, we examined whether balance training improves sleep quality in older adults and how these changes are related to the enhancement of the GABAergic system observed after balance interventions. Based on previous balance intervention studies, which demonstrated a positive influence on the GABAergic system in the sensorimotor cortex, we hypothesized that balance training could be a valuable, non‐pharmacological alternative for addressing sleep difficulties in older adults.

In line with our hypothesis, we demonstrate improvements in subjective sleep quality after three months of balance training. Furthermore, participants demonstrated significantly enhanced GABA levels (MRS measurement) as well as stronger functional connectivity between left and right sensorimotor cortices (functional magnetic resonance imaging (fMRI) measurement) after training. Finally, greater training‐induced up‐regulation of intracortical inhibition during sleeping (TMS measurement) was correlated with more pronounced improvements in sleep quality. Thus, we propose balance training to present a safe and effective alternative or at least a complement to the currently prescribed sleep medication with serious adverse side effects.

## Methods

### Ethical approval

The study was completed in accordance with the *Declaration of Helsinki*, except for pre‐registration in a publicly accessible database. All procedures were approved by the local ethics committee (Commission cantonale d’éthique de la recherche sur l’être humain (CER‐VD); ID: 2021‐00378). All participants provided written informed consent before testing.

### Participants

Forty volunteers aged 64 to 81 years were recruited for the study and equally divided into a balance training group and a control group. An *a priori* power analysis was performed using G*Power 3.1 (Faul et al., 2009) for a repeated‐measures ANOVA with *k* = 2 time points and two groups (group × time interaction). Assuming a small‐to‐medium effect size for healthy older adults (Cohen's *F* = 0.20), α = 0.05, power = 0.80, and correlation among repeated measures *r* = 0.68 (Knutson et al., 2006), the required sample size was *N* = 17 participants per group (total *N* = 34). With an estimated drop‐out rate of 15%, we recruited 20 participants per group. Participants were recruited through newspaper advertisements, followed by an information session to provide participants with more detailed information about the study procedures, screen for exclusion criteria, and to confirm availability for the required time commitment. Participants received appropriate compensation in accordance with the guidelines of the ethics commission. All participants were free from any known neurological and orthopaedic diseases or injuries and contraindications to magnetic resonance imaging (MRI) and TMS measurements.

Two participants from both groups dropped out before the POST tests due to injury, sickness or difficult family situations. Consequently, 18 participants in the balance group (10 females, 71.2 ± 4.1 years old) and in the control group (10 females, 69.6 ± 4.6 years old) were included in the analysis.

### Study design

**Figure 1:**
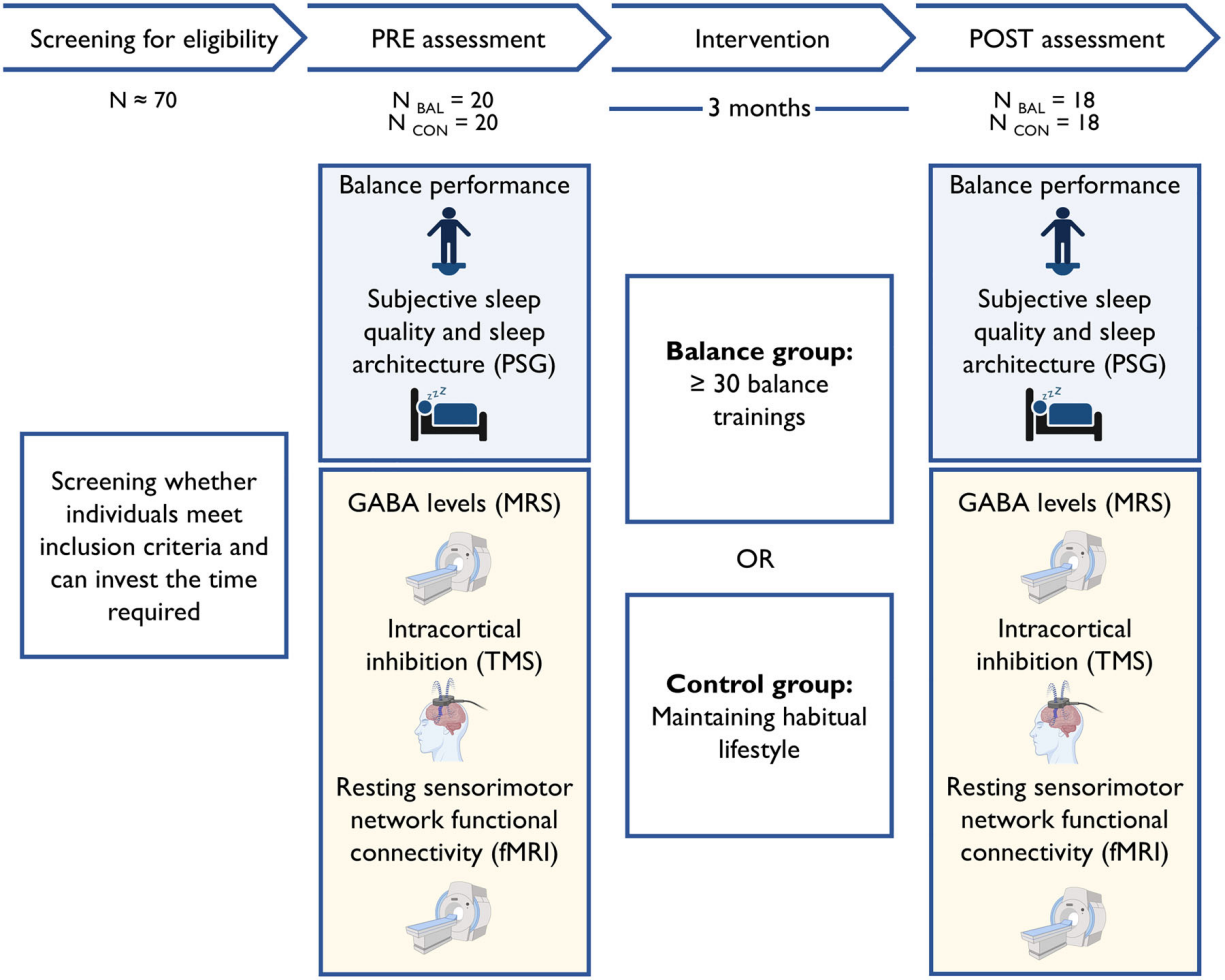
Study design Illustration following the study process from left to right with behavioural measurements in blue boxes and neural measurements in yellow boxes. fMRI, functional magnetic resonance imaging; MRS, magnetic resonance spectroscopy; PSG, polysomnography; TMS, transcranial magnetic stimulation. Created in BioRender (https://BioRender.com/htk4k8m).

The study design included a 12‐week intervention period, preceded by baseline (PRE) and followed by post‐intervention (POST) measurements (see Fig. 1). PRE and POST tests assessed balance performance, subjective and objective sleep quality, GABA levels in the left sensorimotor cortex by means of MRS, intracortical inhibition during a balance task and during sleep with a paired‐pulse TMS protocol, and resting‐state sensorimotor network functional connectivity using fMRI. Participants were randomly assigned to either the balance training group (BAL) or the control group (CON). Participants assigned to the control group were offered participation in a balance training programme after completing the study and received a booklet at study end with instructions for balance exercises to do at home.

### Intervention period

Participants in the balance group engaged in three supervised group training sessions per week for 12 weeks, totalling at least 30 sessions. The balance intervention was planned according to the meta‐analysis of Lesinski and colleagues (Lesinski et al., 2015) who showed that 11–12 weeks of balance training with a frequency of three sessions per week and with a duration of 31–45 min had the largest effects on balance performance in adults aged 65 years and older.

In our study, each session lasted 1 h and included a warm‐up, a main part of 45 min with different balance exercises, and a cool‐down. For example, participants practised standing on one leg on mobile and uneven surfaces, standing on narrow surfaces or standing with both legs on wobble boards aiming to stabilize the board in a horizontal position. Exercise difficulty progressively increased and, whenever possible, the exercises were organized in a playful manner. The exercises could be adapted to be easier with additional support or to be more difficult by reducing the base of support or adding a secondary task (e.g. catching and throwing a ball to a partner). A more detailed description of the balance training plan is provided in the Appendix.

Meanwhile, participants in the control group were instructed to maintain their lifestyle during the 12 weeks between the PRE and POST tests. To monitor potential baseline differences in habitual physical activity between the groups or changes in the control group throughout the study, participants reported exercise frequency (sessions per week), weekly exercise duration and activity type(s) at PRE and POST.

### Balance performance assessment

To assess balance performance, participants stood with both feet and hands akimbo on a wobble board. The wobble boards have a rounded bottom to create an unstable surface, and the participants were instructed to remain as stable as possible for 20 s. The wobble boards were placed on a force plate (508 × 464 mm; OR 6–7 force platform; Advanced Mechanical Technology Inc., Watertown, MA, USA) to record the centre of pressure movements. The force plate signals were sampled at a frequency of 4 kHz and amplified 4000 times (GEN 5, Advanced Mechanical Technology Inc.). We used four different difficulty levels, each with a progressively smaller base of support. Initially, participants had time to find a stable position and perform a familiarization trial. Then, they had two 20 s attempts at the easiest level. If they completed at least one of the two attempts without holding onto the security rail, they progressed to the next level, where they also had a familiarization trial before two recorded attempts. To assess balance performance, the sway area on the most difficult level completed at PRE was analysed. The same level was analysed for the POST measurement. Data were analysed using MATLAB (Version 2021b, The MathWorks, Inc.). The sway area was calculated as the smallest ellipse covering 95% of the centre of pressure data points using the following formula:

(1) $$\begin{array}{ccc} Area & = & \pi \times 2.4478\\ & \times & \sqrt{eigenvalues\;of\;the\;covariance\;matrix\;of\;COP} \end{array}$$

Smaller sway areas indicate better balance performance. The sway area has been used in previous studies and provides a measure of the extent of postural sway (Low et al., 2017). The mean sway area in cm^2^ of the two attempts on the highest wobble board level achieved at PRE was calculated for performance assessment at PRE and POST.

### PSQI to assess subjective sleep quality

The PSQI was used to assess subjective sleep quality over the four weeks preceding the completion of the questionnaire (Buysse et al., 1989). The validated, clinically relevant and widely used questionnaire consists of 19 questions that evaluate seven components, namely the perceived sleep quality, sleep latency, sleep duration, sleep efficiency, sleep disturbances, use of sleep medication and daytime dysfunction. The sum of these components yields a total score ranging from 0 to 21 points, with lower scores indicating better sleep quality. Healthy sleepers usually have a maximum of 5 points; bad sleepers reach a score between 6 and 10; and individuals with chronic sleep disturbances typically reach scores over 10 points. One person was excluded from the analysis of subjective sleep quality due to health complaints during the POST measurement period that interfered with their perceived sleep quality. PSQI data were square root transformed before statistical analyses to ensure normal distribution of residuals.

### PSG to assess objective sleep architecture

We assessed objective sleep architecture with mobile PSG devices at the participant's home (SOMNOtouch RESP, SOMNOmedics, Randersacker, Germany). Participants had one adaptation night, followed by one sleep recording before and one after the three‐month period (Wick et al., 2024). Six single gold cup electroencephalographic (EEG) electrodes were positioned according to the international 10–20 system (Fpz, Cz, Fz, Pz, M1, M2) (Chatrian et al., 1985). Electromyographic (EMG) and electrooculographic (EOG) electrodes were attached following the American Academy of Sleep Medicine (AASM) recommendations (Iber et al., 2007). The recorded data were processed using BrainVision Analyzer 2.2 (Brain‐Products, Ing., GmbH). EEG recordings were referenced to the average of the two mastoids and filtered according to AASM guidelines (Iber et al., 2007). The data were segmented from ‘lights off’ to ‘lights on’. We employed the deep learning‐based sleep staging algorithm U‐Sleep for sleep scoring (Perslev et al., 2021). Each 30 s epoch was classified as wake, N1 sleep, N2 sleep, N3 sleep or rapid‐eye‐movement (REM) sleep and the scoring was verified by an experienced sleep researcher. The total sleep duration, sleep efficiency, sleep onset latency, percentage in each sleep stage and percentage of wakefulness after sleep onset were calculated to describe sleep architecture.

### MRS to assess cortical GABA levels

Both MR measurements were performed on a 7‐T/68 cm MR scanner (Magnetom, Siemens Medical Solutions, Erlangen, Germany) equipped with 80 mT/m gradients and with a single‐channel quadrature transmitter and a 32‐channel receiver coil (Nova Medical Inc. MA, USA). A high‐resolution MP2RAGE (Marques et al., 2010) sequence was used for MRS voxel placement (voxel size = 0.6 × 0.6 × 0.6 mm^3^, TR/TE = 6000/4.94 ms, TI1/TI2 = 800/2700 ms, slice thickness = 0.6 mm, field of view = 192 × 192 × 154 mm^3^, acquisition time = 10 min 8 s).

MRS was used to quantify GABA concentrations in the left sensorimotor cortex using an edited single‐voxel spectroscopy acquired by the MEGA‐sSPECIAL (TR/TE = 4000/80 ms) sequence (Lim & Xin, 2022). Acquisition parameters included: average number = 128, number of datapoints = 2048, spectral width = 4000 Hz, volume of interest = 30 × 20 × 20 mm^3^, acquisition time = 8 min 32 s. To suppress co‐edited macromolecule signals, the sequence employed a broadband asymmetric adiabatic inversion pulse with 500 Hz of inversion bandwidth applied at 1.9 ppm (edit‐on) and highly selective Gaussian pulse with 98 Hz of inversion bandwidth applied at 1.7 ppm (edit‐off) to suppress coedited macromolecule signals. A dielectric pad was positioned near the sensorimotor cortex to improve the transmit field efficiency (Teeuwisse et al., 2012). The MRS voxel was placed in the left precentral gyrus based on our SICI measurement during sleep in the right first dorsal interosseus (FDI) muscle, and on findings from our previous fMRI study, which assessed brain activation during observation and motor imagery of balance tasks (Taube et al., 2015).

MR spectra were averaged after frequency drift and phase correction using FID‐A (Simpson et al., 2017) and analysed by LCModel (Provencher, 2001) for quantification. MEGA difference spectra were fitted with a simulated basis set of five metabolites: GABA, glutamate (Glu), glutamine (Gln), *N*‐acetylaspartic acid (NAA), and *N*‐acetylaspartylglutamate (NAAG). The spectral range for LCModel analysis was set from 1.8 to 4.2 ppm except the nuisance signal in the range between 2.2 and 2.8 ppm.

Absolute metabolite concentrations were referenced to the unsuppressed water signal. To account for inter‐individual differences in tissue composition, all metabolite concentrations were corrected based on voxel tissue segmentation using SPM12 (https://www.fil.ion.ucl.ac.uk/spm/software/spm12/) and transverse (T_2_) relaxation time and tissue‐specific water concentration values (Dhamala et al., 2019). Four individuals were excluded from analysis of GABA levels because of insufficient data quality.

### Electromyography

To record motor evoked potentials (MEPs) in the right tibialis anterior (TA) muscle during balancing and in the FDI muscle during sleep, the overlying skin was prepared by shaving, abrasion and disinfection. Two surface electrodes (Ag/AgCl, Ambu Blue Sensor N, Ballerup, Denmark) were placed over the muscle belly and connected to a transmitter (Myon aktos, Myon AG, Schwarzenberg, Switzerland). The signal was amplified by a factor of 1000 and wirelessly transmitted to the receiver station of the aktos system. The EMG data were recorded at a sampling frequency of 4 kHz.

### Short interval intracortical inhibition

SICI was assessed in the TA while participants were balancing on a challenging wobble board and in the FDI while participants were falling asleep during an afternoon nap. The TA was selected based on prior research demonstrating that age‐related alterations in intracortical inhibition are more prominent in this muscle compared with the soleus and the FDI was chosen because its lower motor threshold permits less intense stimulation during sleep (Papegaaij et al., 2016; Wassermann et al., 1992). We applied a paired‐pulse TMS protocol with a figure‐of‐eight coil (D‐B80, Magventure A/S, Farum, Denmark) connected to a MagPro X100 stimulator equipped with the MagOption system (MagVenture A/S, Farum, Denmark). The optimal positions over the left primary motor cortex (M1) for eliciting MEPs in the right TA or in the right FDI were determined while the participant was sitting on a chair. These so‐called hotspots were marked directly on the scalp. Afterwards, the motor thresholds (MTs) were determined as the lowest stimulation intensity that evoked an MEP of 0.05 mV in at least three out of five consecutive trials in standing position for the TA or in awake supine position for the FDI (Rossini et al., 2015).

Stimulation intensity of the single‐pulse stimulation was set at 120% MT. Stimulation intensities of the paired‐pulse stimulations were set at 70% MT for the conditioning pulse and 120% MT for the test pulse. The conditioning pulse and test pulse were separated by 2 ms to assess GABA_A_‐receptor activity (Abbruzzese & Trompetto, 2002; Di Lazzaro et al., 2007). The protocol was individually adapted if there was no inhibition after 20 stimulations in standing position or in awake lying position. The same relative intensities were used for the POST measurements but adapted to the redetermined MT. Single‐ and paired‐pulse stimulations were applied in alternating order with an interstimulus pause of 4 s for SICI while balancing, or of 6 s for SICI while sleeping.

#### Set‐up while balancing

While the participant was balancing on the wobble board, the TMS coil was fixed over the determined hotspot with a custom‐built helmet attached to the participant's head (for a figure showing the set‐up see Kuhn et al. (2024)). Two sets of 20 stimulations were applied while the participant balanced on the most challenging wobble board they successfully completed at PRE. Between the two sets, the participants could rest for approximately 1 min.

#### Set‐up during afternoon nap

SICI during sleep was assessed during an afternoon nap, which allowed controlled, feasible acquisition during the wake‐to‐sleep transition. For offline sleep scoring of the afternoon nap, participants were equipped with electrodes for EEG, EMG and EOG measurements. Eight single gold cup electrodes were positioned according to the international 10–20 system (Fpz, F3, F4, Fz, M1, M2, O1, O2) (Chatrian et al., 1985). EMG and EOG electrodes were attached following the AASM recommendations (Iber et al., 2007). The recorded data were processed using BrainVision Analyzer 2.2 (Brain‐Products, Ing., GmbH). EEG recordings were referenced to the contralateral mastoid and filtered according to AASM guidelines (Iber et al., 2007). TMS artefacts were removed through automatic blink detection over F3, followed by automatic ocular correction with independent component analysis and pulse artefact correction. The data were segmented from ‘lights off’ to ‘lights on’. We only used the right electrodes for sleep scoring and employed the deep learning‐based sleep staging algorithm U‐Sleep scoring (Perslev et al., 2021) in addition to a sleep scorer who visually classified the epochs. Each 30 s epoch was classified as wake, N1 sleep, N2 sleep, N3 sleep or REM sleep. In case of disagreement between the sleep scorer and the algorithm‐based scoring, a second expert scorer was consulted. N1 sleep is the light sleep phase between being awake and in stable sleep and the MT in this stage is not yet significantly higher compared with lying awake (Avesani et al., 2008). Stimulations during N1 were exported for SICI calculation. SICI assessment during N1 enabled us to determine the MT while the participant was lying awake and to then deliver automated stimulations at pre‐calculated intensities and at a regular rhythm, thereby minimizing disturbance during the sleep opportunity. Thus, SICI measurement during N1 sleep allowed us to measure functionally relevant modulation of inhibition during sleep, without having to interrupt the rhythmic stimulations to reassess the motor threshold throughout the afternoon nap.

To verify whether the TMS was still in place throughout the nap, we attached three reflective markers on the TMS coil and three reflective markers on the participant's head. Throughout the afternoon nap, the positions of the markers were recorded with Motive motion capture software version 1.7.2 and five OptiTrack motion capture cameras (Prime 17W, NaturalPoint Inc., Corvallis, OR). The tracking data were exported and analysed using MATLAB (Version 2021b, The MathWorks, Inc.). Data were excluded if the stimulation point deviated more than 7 mm from the determined hotspot, representing a conservative cutoff relative to prior evidence showing that conditioning pulses within approximately 8 mm of the hotspot contribute to SICI (Nieminen et al., 2019).

#### SICI analyses

Recorded EMG data were analysed using MATLAB (Version 2021b, The MathWorks, Inc.). The raw EMG data were band‐pass filtered (100–1000 Hz), and the peak‐to‐peak amplitudes (mV) were calculated between 35 and 80 ms after the stimulation. Background EMG activity (bEMG) was calculated as the root mean square of the EMG signal in the time window of 50 ms prior to the TMS stimulation. MEPs with a bEMG more than three times the median absolute deviation from the median bEMG were excluded from data analysis (Leys et al., 2013). SICI values were calculated as the percentage difference between conditioned paired‐pulse and unconditioned single‐pulse MEPs using the formula 100 − (paired‐pulse/single‐pulse × 100) (Kuhn et al., 2017; Mouthon & Taube, 2019). One person did not complete the SICI while balancing task, as this person was feeling unwell after the MT determination at PRE. We transformed the SICI while balancing data with Yeo‐Johnson transformation to reach normally distributed residuals, as the data included some negative values. Regarding SICI during sleep, six participants did not fall asleep at both time points, two participants were excluded from analysis because they moved their head more than 7 mm away from the hotspot before falling asleep, and two participants had to be excluded because of clearly negative SICI values (−25% or lower). We were able to assess SICI during sleep in 11 participants in the balance group and in 15 participants in the control group. Over the 26 included participants, we analysed 2664 stimulations (48 ± 37 stimulations per participant).

### Resting‐state sensorimotor network functional connectivity

Resting‐state fMRI images were acquired using a 2D multi‐slice gradient‐echo echoplanar imaging sequence (TR = 1550 ms, TE = 26 ms, field of view = 198 × 198 mm^2^, in‐plane resolution = 1.3 × 1.3 mm^2^, slice thickness = 1.3 mm, 100 slices, GRAPPA factor = 3, acquisition time = 6 min 52 s) (Moeller et al., 2010).

Resting‐state fMRI data were processed using a custom pipeline. First, the initial five time points of functional data were removed to ensure magnetic field stabilization. Functional and structural brain images were skull‐stripped using SynthStrip. The skull‐stripped functional images were then corrected for head motion using MCFLIRT (Jenkinson et al., 2002) implemented in FSL (https://fsl.fmrib.ox.ac.uk/fsl/fslwiki/FSL). Next, susceptibility distortion correction was performed using SynBOLD‐Disco (Yu et al., 2023). In this pipeline, a ‘synthetic, undistorted’ BOLD image that matches the geometry of structural T1w images and the T2* contrast was generated using a pre‐trained neural network. This image was then used as reference for FSL TOPUP for distortion correction (Andersson et al., 2003). The distortion‐corrected images were then transformed to Montreal Neurological Institute (MNI)‐152 2 mm space by first registering functional image to structural image with epi_reg functionality in FSL, followed by registering from structural space to MNI space using antsRegistrationSynQuick functionality implemented in Advanced Normalized Tools (https://github.com/ANTsX/ANTs). The transformed images were smoothed using a Gaussian kernel of full width at half‐maximum of 4 mm. Next, the images in standard space were further processed with ICA‐AROMA (Pruim et al., 2015), an independent component analysis (ICA) based method for removing motion‐related artefacts. After this step, white matter and cerebrospinal fluid signals were regressed from the images. Finally, a high‐pass filter of 0.01 Hz was applied to remove low‐frequency drifts.

The processed fMRI images were used as inputs to a standard group ICA followed by a dual‐regression procedure to extract subject‐specific canonical resting‐state networks (RSNs) (Stagg et al., 2014). In this step, the functional data from all subjects and all sessions were first concatenated to create a single 4D dataset. Group‐ICA with 25 components was then performed on this dataset and RSNs of the sensorimotor network were identified using spatial correlations against previously defined maps (Yeo et al., 2011). The number of independent components was chosen based on previous resting‐state functional connectivity studies (Smith et al., 2014; Stagg et al., 2014). Subject‐specific RSN maps were derived using dual regression (Nickerson et al., 2017). This process involved using all 25 components to perform spatial regression against each separate fMRI dataset and then using the resulting normalized time‐course matrices to perform a temporal regression to estimate subject‐specific maps that reflect the participant specific strength of functional connectivity. In these subject‐specific spatial maps, voxel values represent the strength of association with the group ICA of interest. The resulting participant specific RSN map was then masked by the group‐mean RSN map and the mean value within the network of interest was extracted for each subject, used as a measure of the strength of functional connectivity within the RSN. The ventral subdivision of the sensorimotor network was selected as the network of interest for further analysis, as it contains neurons corresponding to the FDI muscle targeted by the SICI measurement during sleep.

### Statistical analysis

Statistical analyses were conducted using RStudio version 4.4.1 (https://www.r‐project.org/). Descriptive statistics were expressed as means (M) with standard deviations (SD). Analyses were considered significant when the *P* value was smaller than α = 0.05. We compared weekly exercise frequency and duration at baseline between balance and control group with unpaired *t* tests and the changes from PRE to POST in the control group with paired *t* tests. Comparisons of changes over time between groups were assessed by two‐way repeated‐measures ANOVA. Assumptions were assessed with normal q–q plots of model residuals, Levene's test to test for similar variances across groups, and Bartlett's test to assess sphericity. In case of significant interactions, changes within the two groups were assessed with paired *t* tests and *P* values were adjusted using the Bonferroni correction for two comparisons. All estimates of the interaction between group × time were reported with the effect size partial eta squared (*η^2^*). The *η^2^* values were interpreted as negligible (<0.01), small (0.01–0.059), moderate (0.06–0.139) and large (≥ 0.14) according to Cohen (Cohen, 2013). Changes over time per group were reported using relative or absolute changes and the standardized effect size Cohen's *d*. Cohen's *d* values were interpreted as negligible (<0.2), small (0.2–0.5), moderate (0.5–0.8) or large (≥0.8) effects (Cohen, 2013). Further, we conducted a multiple linear regression analysis to examine how changes in the proposed underlying mechanisms predicted changes in subjective sleep quality with the following formula and *z*‐transformed parameters to allow for direct comparison of effect sizes:

(2) $$\begin{array}{ccc} \Delta \mathrm{PSQI} & = & b_{0}+b_{1}*\Delta \mathrm{GABA}\_\mathrm{levels}\\ & & +\;b_{2}*\Delta \mathrm{functional}\_\mathrm{connectivity}\\ & & +\;b_{3}*\Delta \mathrm{SICI}\_\mathrm{balance}\\ & & +\;b_{4}*\mathrm{PRE}\_\mathrm{PSQI}+\varepsilon \end{array}$$

Model assumptions were evaluated using the Durbin–Watson test for residual autocorrelation and variance inflation factors (VIF) to assess multicollinearity among predictors (VIF < 5). For the modulation of intracortical inhibition we included SICI while balancing in the model, because there were too many missing data points for SICI while sleeping. Instead, we analysed the Pearson correlation coefficient between changes in PSQI scores and changes in SICI during sleep, to assess the functional specificity of SICI during sleep. To assess how the change in balance performance was associated with the change in subjective sleep quality, we calculated the Pearson correlation coefficient between changes in sway area and changes in PSQI within the balance group. We further wanted to verify the specific functional significance of SICI during balance performance from previous literature (Kuhn et al., 2024; Mouthon & Taube, 2019; Taube et al., 2020), so we calculated the Pearson correlation coefficients between changes in SICI and changes in balance performance. Assumptions for correlation analyses were verified with q–q plots for normal distribution of the difference scores of each included parameter and with screening for outliers more than three SDs away from the mean. Where outliers were detected, we assessed correlations with and without outliers to verify the result.

#### Exploratory analyses

In addition to the primary hypotheses and associated analyses that were defined *a priori* based on previous literature and our study objectives, we performed the following additional analysis. As the effect of ΔGABA levels on ΔPSQI was negative, we wanted to test whether our data support an interpretation in the context of the model suggested by Lauber & Taube (2024). Therefore, we calculated the Pearson correlation coefficient between baseline GABA levels and the change in GABA levels.

### Training specificity – resistance training control group

The primary comparisons relevant to the hypotheses of this study focused on the BAL and CON group. We added a resistance training group (RT) which served as an active control to assess specificity of balance training effects relative to general physical activity.

#### Participants

Twenty volunteers aged 65 to 79 years were recruited through newspaper advertisements and an information session after first mention of interest. All participants were free of any known neurological and orthopaedic diseases or injuries and contraindications to MRI and TMS measurements. Participants received appropriate compensation in accordance with the guidelines of the ethics commission. Two participants dropped out before POST tests due to detected diseases during the study intervention. Consequently, 18 participants (7 females, 70.11 ± 4.00 years old) were included in the analysis.

#### Resistance training intervention

Similar to the balance training intervention, the resistance training was conducted in groups. Participants attended supervised sessions three times a week, totalling at least 30 training sessions of 45 min each. Each session included a warm‐up, the main part and a cool‐down. The warm‐up and cool‐down together taking approximately 15 min and the main part 30 min. The main part of the session consisted of four exercises targeting the lower extremities. Each session included one exercise for ankle dorsiflexion, ankle plantarflexion, knee flexion and knee extension. The intensity of the resistance training progressively increased through adjustments in the number of repetitions, sets and external load, as well as the inclusion of explosive exercises to provide optimal stimuli (Fragala et al., 2019). The most relevant exercise for this study was ankle dorsiflexion, while the other exercises were added to make the training more beneficial and attractive for the subjects. Ankle dorsiflexion was trained against the resistance of elastic bands. Participants engaged in three weeks of traditional training, during which the number of sets increased from one to three sets of 10–15 repetitions and the resistance band strength was increased. Over the following nine‐week period, the training programme comprised alternating sessions of explosive training (2–3 × 6–10 repetitions, contraction as fast as possible – hold shortly – slow excentric movement back) and traditional training (2–3 × 8–12 repetitions, slow concentric and excentric movement). The rest interval between sets was 1–2 min. The participants added weight cuffs on their foot, increasing the weight by 0.5 kg every week. The resistance was reduced by 1 kg for the explosive executions.

#### Explosive strength performance assessment

The strength assessment was carried out in a seated position on an isokinetic machine (Cybex Norm, HUMAC, CA, USA). The right leg of the participant was placed on the foot pedal of the machine. To achieve the most valid power transmission possible, the participants were equipped with customized shoes directly attached to the foot pedal, additionally attached with Velcro straps. Participants executed isometric ankle dorsiflexion in a neutral zero position and were verbally encouraged to maximize peak rate of torque development (RTD) by fast dorsiflexion. The participants performed two familiarization trials prior to the measurement, which consisted of five trials with at least 15 s of rest between each trial (Maffiuletti et al., 2016). The peak RTD was identified as the steepest point of the RTD force curve using a 20 ms moving window. Valid trials were averaged for PRE to POST comparison. Trials failing to meet requirements (i.e. countermovement or upper body movements) were excluded from the analysis. We had to delete data from four participants, because the participants changed their execution strategy from PRE to POST. Furthermore, we had to delete three trials from two different participants, all other individuals had five valid trials.

#### Statistical analysis

We compared parameters assessed at baseline and post‐intervention with paired *t* tests.

## Results

Self‐reported exercise frequency (*t*(38) = 0.38, *P* = 0.703, *d* = 0.12 [–0.50; 0.74]) and weekly exercise duration (*t*(38) = −0.36, *P* = 0.719, *d* = −0.11 [−0.73; 0.51]) did not differ between groups at baseline. Furthermore, the control group showed no change in self‐reported exercise frequency from PRE to POST (*t*(17) = 0.00, *P* = 0.999, *d* < 0.01 [−0.46; 0.46]) and no change in weekly exercise duration (*t*(17) = −0.62, *P* = 0.542, *d* = −0.15 [−0.61; 0.32]). Reported types of sports also remained unchanged over time. These self‐reported measures suggest that the control group maintained their habitual exercise behaviour over the intervention period, supporting comparison of the balance intervention against a stable habitual‐lifestyle condition.

### Behavioural changes

**Figure 2:**
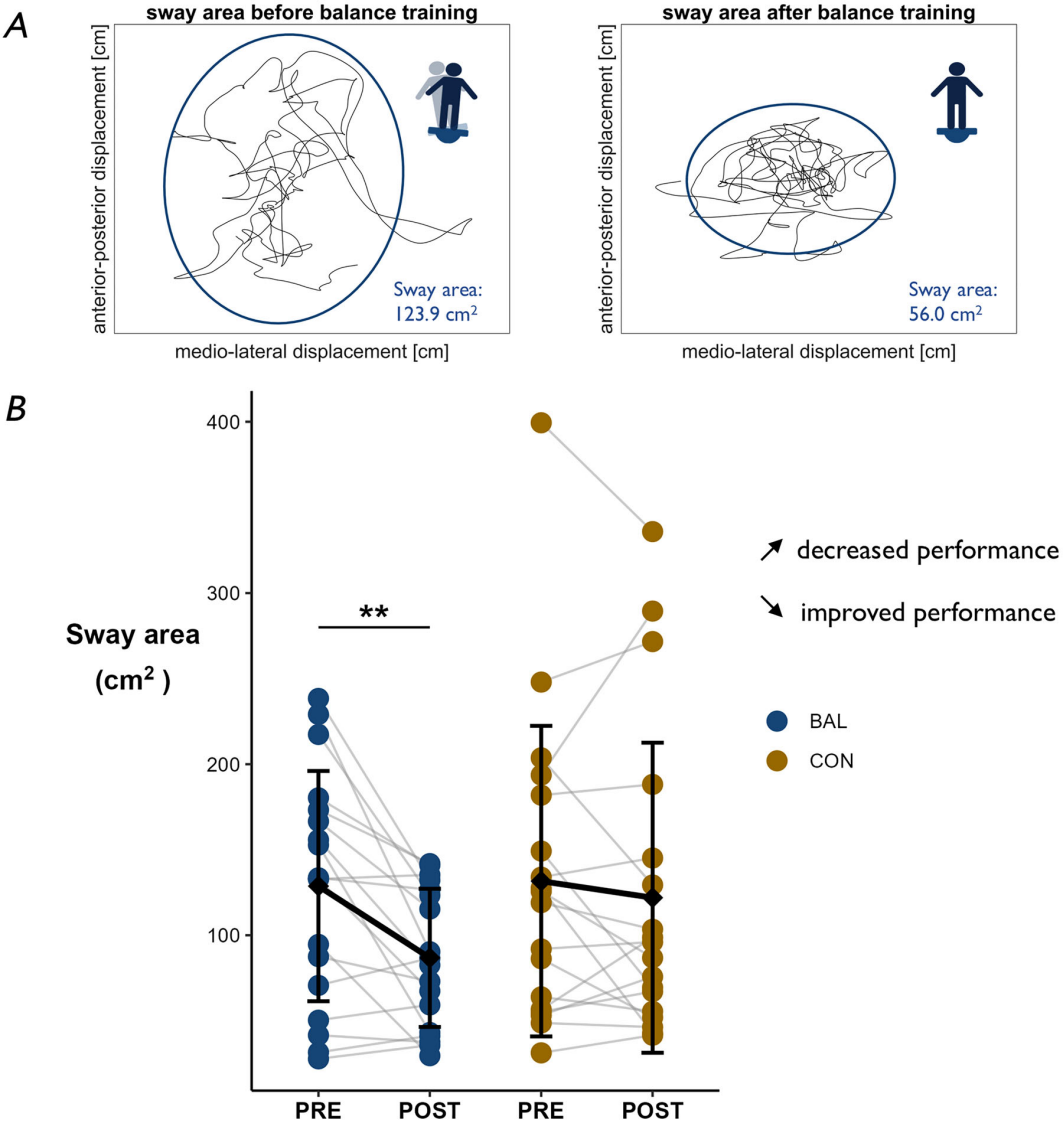
Balance performance assessed by sway area on the wobble board *A*, data of one person in the balance training group showing the movement of the centre of pressure in black and the sway area covering 95% of the centre of pressure data points in blue. The best attempt while balancing on the same wobble board at PRE (Left) and at POST (Right) is shown. *B*, coloured dots show individual data points of participants, mean and standard deviation (SD) per group and time are presented in black. The balance group in blue (*n* = 18) showed a significant decrease in sway area. The mean sway area of the control group in yellow (*n* = 18) did not change significantly. ** *P*_bonF_ = 0.00364 of paired *t* test in change over time after significant group × time interaction.

To confirm whether the balance training programme was sufficient to enhance balance performance after three months of training in older adults, we assessed the sway area in cm^2^ while participants were balancing on a wobble board for 20 s at PRE and at POST (Fig. 2*A*). Balance performance showed a significant group × time interaction by ANOVA (*F*(1, 34) = 4.17, *P* = 0.0490, η^2^ = 0.01; Fig. 2*B*). The balance group showed a mean decrease of 41.94 (SD = 48.24) cm^2^ in sway area (*t*(17) = −3.69, *P*_bonferroni_ = 0.00364, *d* = −0.87 [−1.41; −0.31]), while the control group did not show any change after three months (Δ = −9.67, SD = 46.59, *t*(17) = −0.88, *P*_bonferroni_ = 0.782, *d* = −0.21 [−0.67; 0.26]). At POST, mean sway area was 35.18 cm^2^ smaller in the balance group (86.78 cm^2^) than in the control group (121.96 cm^2^); however, this between‐group difference did not reach statistical significance. Together, these results indicate that balance performance improved over time in the balance training group relative to the control group.

**Figure 3:**
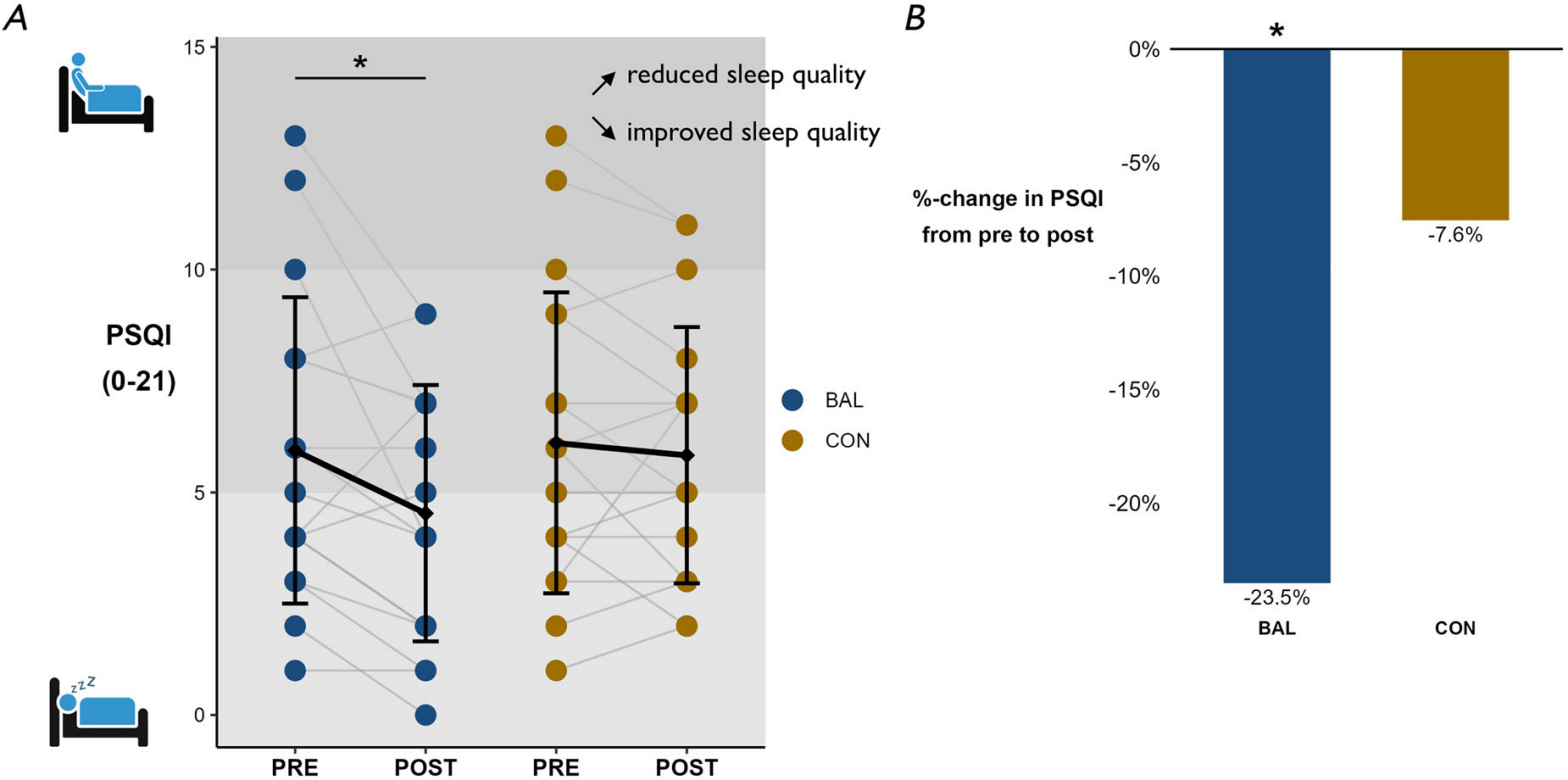
Subjective sleep quality assessed with the PSQI questionnaire *A*, dots show the individual data points of participants, mean and SD per group and time are presented in black. The balance group in blue (*n* = 17) showed a significant improvement in PSQI score. The score of the control group in yellow (*n* = 18) did not change significantly. *B*, bars show the percentage change from PRE to POST. * *P*_bonF_ = 0.0199 paired *t* test in change over time after significant group × time interaction. Colour coding in *A*: Healthy sleepers (0–5 score points), bad sleepers (6–10 score points), chronic sleep disturbances (over 10 points); PSQI, Pittsburgh Sleep Quality Index. Created in BioRender (https://BioRender.com/htk4k8m).

To assess the effects of balance training on subjective sleep quality, we employed the global score of the PSQI questionnaire, which distributes individuals into healthy sleepers (0–5 points), bad sleepers (6–10 points) and persons with chronic sleep disturbances (11–21 points) (Buysse et al., 1989). The improvements in balance performance were accompanied by changes in subjective sleep quality (Fig. 3*A*), which showed a significant group × time interaction (*F*(1, 33) = 4.67, *P* = 0.0381, *η^2^* = 0.01). After the balance intervention, subjective sleep quality was improved by a mean of 1.41 (SD = 2.21) score points (*t*(16) = −2.92, *P*_bonferroni_ = 0.0199, *d* = −0.71 [−1.23; −0.17]), indicating a 23.8% decrease (Fig. 3*B*). More specifically, the mean score of the balance group dropped from the bad‐sleepers category before the training to the healthy‐sleepers category after the training (Fig. 3*A*). Moreover, no more individuals were in the category of chronic sleep disturbances after three months of balance training. In contrast, there was no significant change after three months in the control group (Δ = 0.28, SD = 1.71, *t*(17) = −0.36, *P*_bonferroni_ = 0.999, *d* = −0.09 [−0.55; 0.38]). The change in subjective sleep quality within the balance group showed a trend to a positive correlation with the change in balance performance (*r*(15) = 0.47, *P* = 0.0555). In summary, participants improved not only their balance performance but also enhanced their subjective sleep quality after three months of balance training.

We also investigated the effect of balance training on objective sleep parameters, such as sleep efficiency, time to fall asleep and the proportion of the night in deep sleep. However, we did not see any significant changes from PRE to POST in either of the groups (Table 1).

**Table 1:** Objective sleep data

	Balance group			Control group		
Parameter	PRE Mean ± SD	POST Mean ± SD	Paired *t* test uncorrected *n* = 15	PRE Mean ± SD	POST Mean ± SD	Paired *t* test uncorrected *n* = 17
**TIB (min)**	463.3 ± 56.1	455.5 ± 79.4	*t* = 0.52, *P* = 0.609, *d* = 0.14 [−0.38; 0.64]	487.0 ± 64.6	479.5 ± 63.6	*t = *1.36, *P = *0.194, *d = *0.33 [−0.16; 0.81]
**SOL (min)**	12.5 ± 8.6	16.1 ± 10.0	*t* = −1.58, *P* = 0.135, *d* = −0.41 [−0.93; 0.13]	28.2 ± 31.1	17.4 ± 11.2	*t* = 1.83, *P* = 0.0855, *d* = 0.44 [−0.06; 0.94]
**WASO (%)**	11.6 ± 7.7	14.4 ± 9.0	*t* = −1.05, *P* = 0.314, *d* = −0.27 [−0.78; 0.25]	11.2 ± 8.4	12.0 ± 8.8	*t* = −0.32, *P* = 0.750, *d* = −0.08 [−0.55; 0.40]
**N1 (%)**	5.2 ± 0.6	5.9 ± 0.5	*t* = −1.10, *P* = 0.288, *d* = −0.28 [−0.80; 0.24]	5.5 ± 2.3	5.2 ± 2.4	*t* = 0.62, *P* = 0.543, *d = *0.1 [−0.33; 0.63]
**N2 (%)**	54.1 ± 6.7	52.5 ± 7.8	*t* = 0.77, *P* = 0.455, *d = *0.20 [−0.32; 0.71]	53.5 ± 8.1	51.9 ± 8.3	*t = *0.81, *P = *0.432, *d* = 0.20 [−0.29; 0.67]
**SWS (%)**	12.6 ± 7.4	11.8 ± 6.0	*t = *1.20, *P* = 0.249, *d* = 0.31 [−0.21; 0.82]	12.9 ± 4.8	13.4 ± 6.1	*t* = −0.42, *P* = 0.678, *d* = −0.10 [−0.58; 0.38]
**REM (%)**	16.6 ± 2.2	15.4 ± 4.5	*t* = 0.88, *P* = 0.396, *d = *0.23 [−0.29; 0.74]	16.9 ± 6.7	17.5 ± 6.8	*t* = −0.60, *P* = 0.555, *d* = −0.15 [−0.62; 0.33]
**TST (min)**	389.9 ± 41.3	366.5 ± 71.2	*t* = 1.35, *P* = 0.198, *d = *0.35 [−0.18; 0.87]	395.3 ± 59.3	391.7 ± 39.7	*t* = 0.29, *P* = 0.776, *d* = 0.0 [−0.41; 0.54]
**SE (%)**	84.6 ± 7.1	80.5 ± 8.6	*t = *1.52, *P* = 0.150, *d* = 0.39 [−0.14; 0.91]	81.6 ± 10.2	82.4 ± 8.2	*t* = −0.30, *P = *0.767, *d* = −0.07 [−0.55; 0.40]

*Note*: Objective sleep quality did not change from PRE to POST in the balance nor in the control group.

Abbreviations: N1, non‐rapid eye movement sleep stage 1; N2, non‐rapid eye movement sleep stage 2; REM, rapid eye movement sleep; SE, sleep efficiency (total sleep time / time in bed); SOL, sleep onset latency; SWS, slow‐wave sleep; TIB, time in bed; TST, total sleep time; WASO, wake after sleep onset.

### Brain changes

#### Biochemical changes assessed with MRS

**Figure 4:**
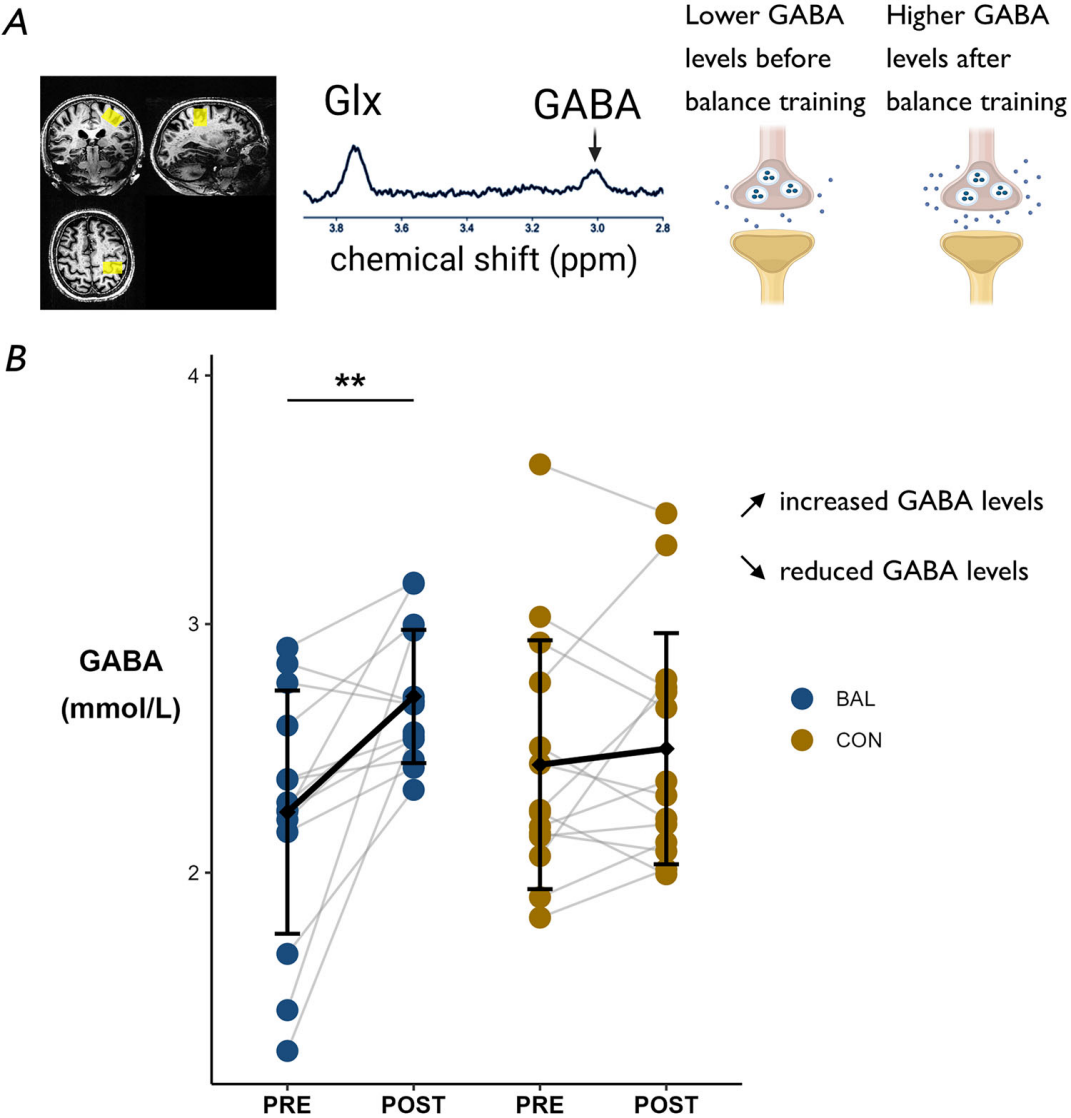
GABA levels in the sensorimotor cortex *A*, illustration of the MRS voxel in the sensorimotor cortex (Left). Illustration of GABA concentration determined by single‐voxel MRS with an exemplary spectrum of a measurement sequence MEGA‐sSPECIAL (Middle). Illustration of MRS measured concentration of the extra‐synaptic neurotransmitter GABA (Right). *B*, dots show the individual data points of participants, mean and SD per group and time are presented in black. The balance group in blue (*n* = 14) showed a mean increase in GABA levels, the GABA levels of the control group in yellow (*n* = 14) did not change significantly. ** *P*_bonF_ = 0.00678 for paired *t* test in change over time after significant group × time interaction. Glx, Glutamate + Glutamine; MRS, magnetic resonance spectroscopy; ppm, parts per million. Created in BioRender (https://BioRender.com/htk4k8m).

To better understand *how* balance training positively affected sleep quality, we looked at changes in the sensorimotor cortex as this area was proposed to be important to suppress motor activity during sleep and possibly relevant changes after balance training have been observed in this area (Chen et al., 2024; Kuhn et al., 2024; Liu & Dan, 2019). As the GABAergic system is known to be involved in sleep regulation (Saper & Fuller, 2017), we assessed changes in GABA concentration (i.e. capacity for inhibition) using MRS (Fig. 4*A*). This analysis revealed a significant group × time interaction in GABA levels (*F*(1, 26) = 6.45, *P = *0.0175, *η^2^* = 0.05; Fig. 4*B*). The balance group showed a mean increase of 0.47 (SD = 0.49) mmol/L in GABA concentration after the training intervention (*t*(13) = 3.58, *P*_bonferroni_ = 0.00678, *d* = 0.96 [0.31; 1.58]). Exploratory analyses showed that greater increases in GABA concentration after balance training were associated with lower baseline GABA levels (*r*(12) = −0.85, *P* < 0.001). The control group did not show a significant change in GABA levels after three months (Δ = 0.06, SD = 0.33, *t*(13) = 0.72, *P*_bonferroni_ = 0.968, *d = *0.19 [−0.34; 0.72]). Thus, three months of balance training led to enhanced GABA levels (i.e. capacity for inhibition) in the sensorimotor cortex and participants with lower baseline levels showed a bigger increase in GABA compared to participants with higher baseline values.

#### Changes in intracortical inhibition assessed with TMS

**Figure 5:**
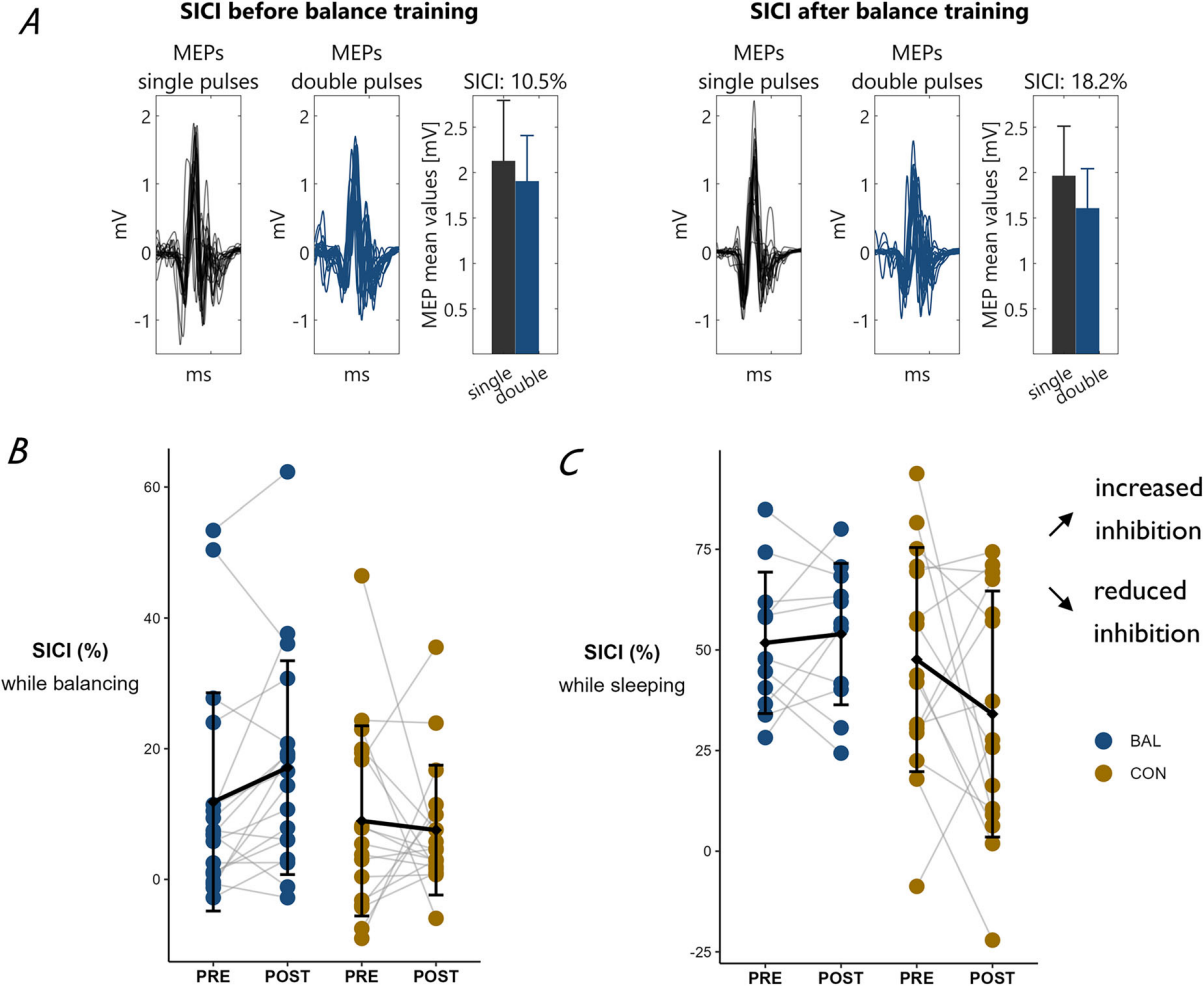
Intracortical inhibition assessed while balancing and while sleeping *A*, graphical presentation of motor evoked potentials by transcranial magnetic stimulations of one participant while balancing on the difficult wobble board before the balance training intervention (Left) and after the intervention (Right). Single pulses: motor evoked potentials after single pulse stimulations. Double pulses: motor evoked potentials after double pulse stimulations. SICI: The ratio of the mean of all peak‐to‐peak amplitudes after single (black) and double (blue) pulse stimulation represents the intracortical inhibition induced by the conditioning pulse. The bar heights represent the mean amplitudes, and the error bars represent the SD. *B*, change in SICI while balancing on the wobble board is shown. Coloured dots represent data points of participants, mean and SD per group and time are presented in black. Balance group in blue (*n* = 18) and control group in yellow (*n* = 17). *C*, change in SICI during sleep for balance group in blue (*n* = 11) and control group in yellow (*n* = 15) is shown. MEP, motoer evoked potential; SICI, short‐interval intracortical inhibition.

To investigate whether the observed increased capacity for inhibition was accompanied by an enhanced ability to task specifically modulate inhibition, we assessed SICI while balancing on a wobble board (see Fig. 5*A*) and while sleeping. SICI did not show significant group × time interaction during balance nor during sleep (balance: *F*(1, 33) = 0.31, *P = *0.584, *η^2^* < 0.01; sleep: *F*(1,24) = 1.81, *P* = 0.191, *η^2^* = 0.03). Nonetheless, there was a 44.2% increase in the balance training group and a 15.7% decrease in the control group in intracortical inhibition during balancing (Fig. 5*B*). Furthermore, participants showed a 4.2% increase after balance training and a 28.4% decrease in the control group in intracortical inhibition during sleep (Fig. 5*C*).

**Figure 6:**
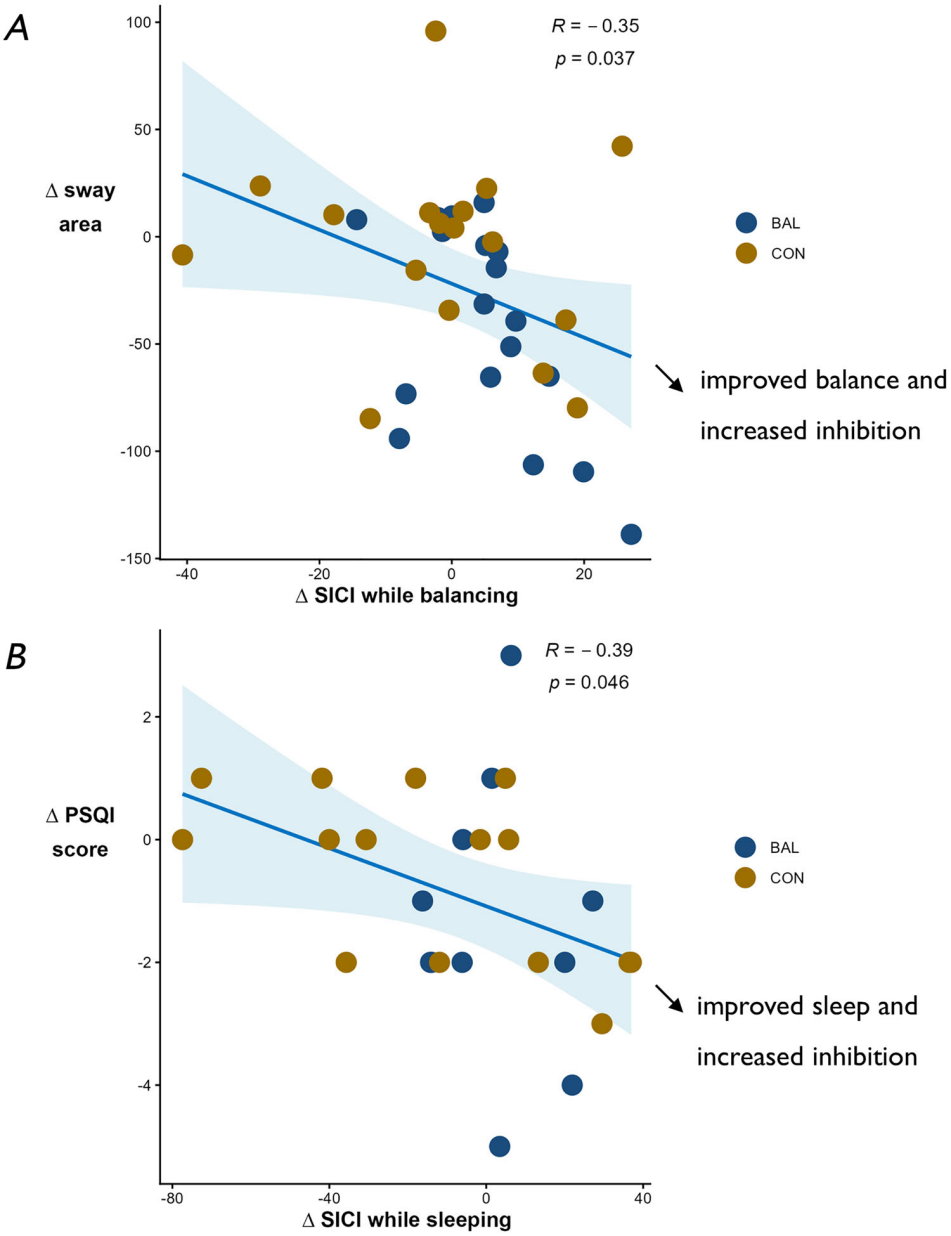
Correlation analyses show functional relevance of SICI measurements The scatterplots show correlations between modulation of intracortical inhibition and behavioural parameters including both groups. Higher SICI values represent increased inhibition. *A*, larger increases in SICI while balancing are associated with greater improvements in balance performance (*n* = 35). *B*, larger increases in SICI while sleeping are associated with greater improvements in subjective sleep quality (*n* = 26). SICI, short‐interval intracortical inhibition; PSQI, Pittsburgh Sleep Quality Index.

To evaluate the functional relevance of SICI changes measured in different conditions, we calculated the correlations between changes in SICI and the corresponding behavioural changes. Participants with larger increases in inhibition (i.e. higher SICI) while balancing showed a greater improvement in balance performance (*r*(33) = −.35, *P* = 0.0367; Fig. 6*A*). Accordingly, participants with larger increases in inhibition (i.e. higher SICI) during sleep showed greater improvements in subjective sleep quality (*r*(24) = −.39, *P* = 0.0460; Fig. 6*B*). These results demonstrate that an enhancement in intracortical inhibition is not only associated with improvements in the trained balance task but also with modulation of inhibition during a different task: falling asleep, where suppression of motor activity is also considered important.

#### Changes in resting‐state functional connectivity assessed with functional magnetic resonance imaging (fMRI)

**Figure 7:**
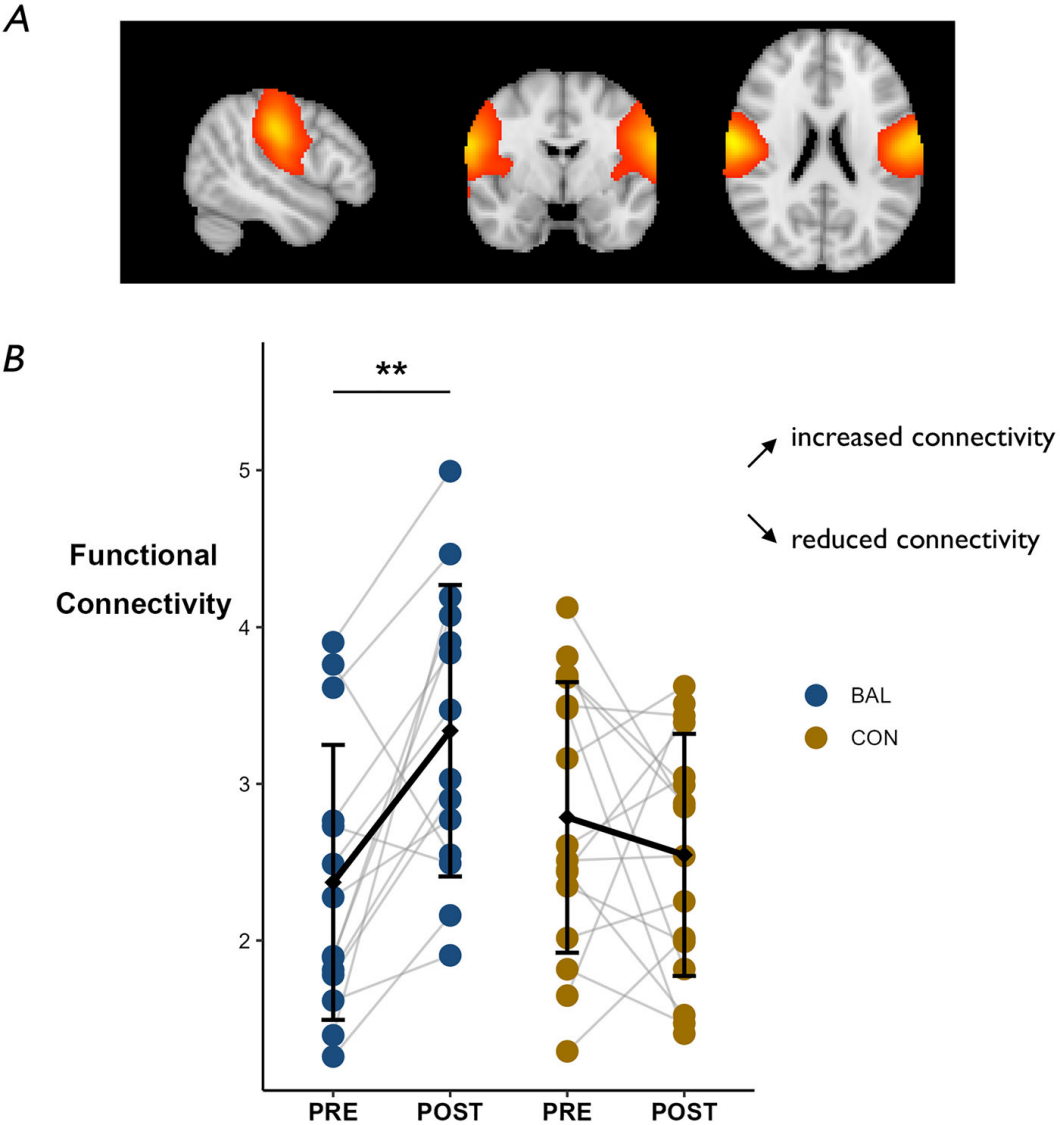
Resting‐state sensorimotor functional connectivity *A*, location of the ventral sensorimotor network detected using group‐ICA on the dataset consisting of all scans and all subjects. Red colours represent higher connectivity strength. *B*, coloured dots represent data points of participants, means and SD per group and time are presented in black. The balance group in blue (*n* = 14) showed a mean increase in functional connectivity. The connectivity strength of the control group in yellow (*n* = 16) did not change significantly. * *P*_bonF_ = 0.00608 for paired *t* test in change over time after significant group × time interaction.

The last neural parameter that has been measured and was hypothesized to play a role in the improvement of sleep quality was resting‐state sensorimotor functional connectivity (Fig. 7*A*). Functional connectivity within the sensorimotor network showed a significant group × time interaction (*F*(1, 28) = 10.34, *P =* 0.00327*, η^2^ = *0.12; Fig. 7*B*). After balance training, participants showed an increase of 0.97 (SD = 1.00) in connectivity strength (*t*(13) = 3.63, *P*_bonferroni_ = 0.00608, *d* = 0.97 [0.32; 1.60]), while the control group did not show a significant change (Δ = −0.24, SD = 1.05, *t*(15) = −0.91, *P*_bonferroni_ = 0.752, *d* = −0.23 [−0.72; 0.27]).

### Regression analyses

**Figure 8:**
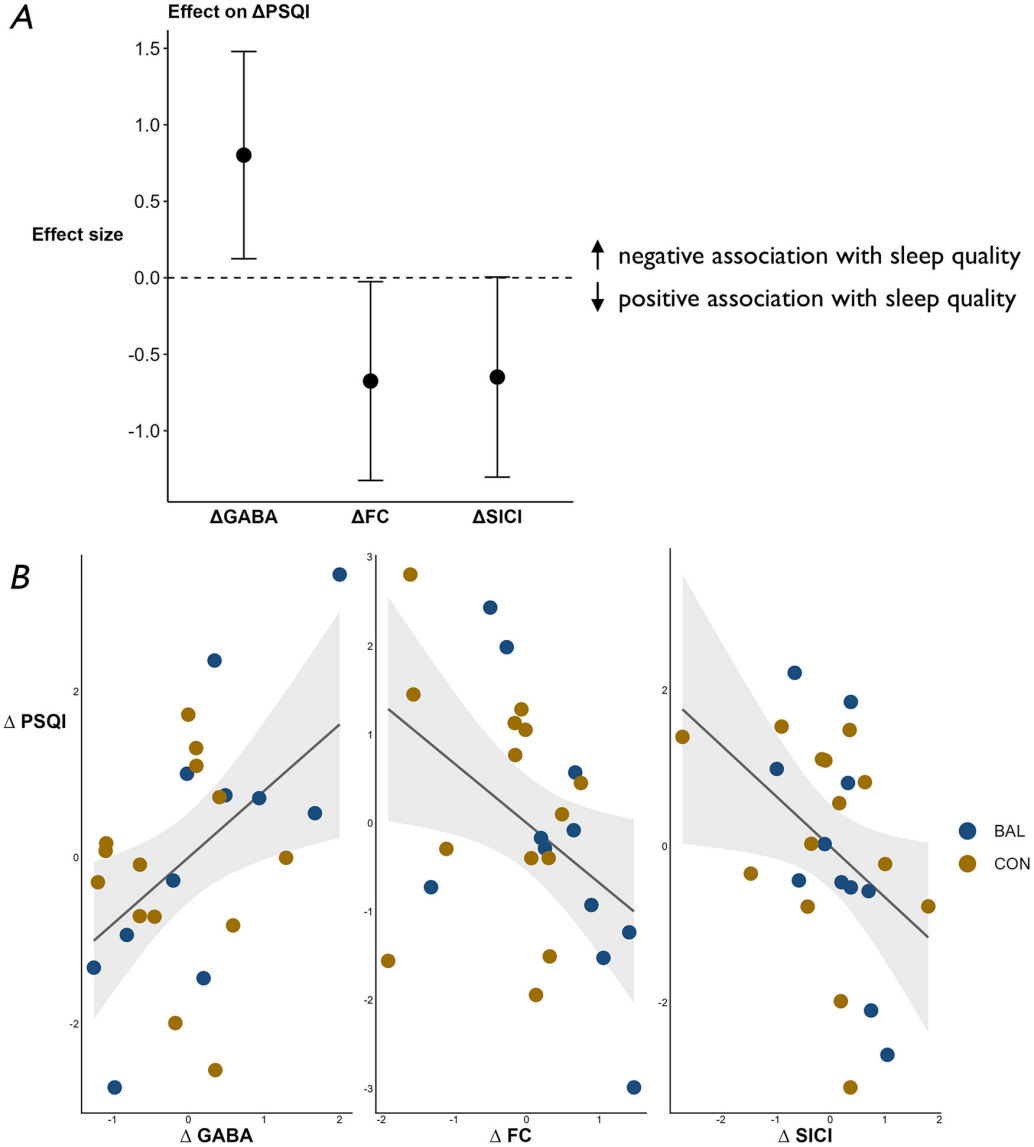
Regression analysis *A*, standardized regression coefficients (β) predicting change in subjective sleep quality (ΔPSQI) are shown. Estimated standardized effect sizes for each predictor: change in GABA levels (ΔGABA), change in functional connectivity (ΔFC) and change in SICI while balancing (ΔSICI) from the multiple linear regression model (ΔPSQI ∼ ΔGABA + ΔFC + ΔSICI + PRE_PSQI). Bars represent 95% confidence intervals around each β estimate. The horizontal dashed line at zero denotes no effect. *B*, partial regression plots illustrating the associations between each predictor and change in sleep quality after controlling for the other variables in the model. The regression line with 95% confidence interval reflects the slope of each predictor as estimated in the multiple linear regression model. Individual data points are coloured by group (BAL in blue, CON in yellow). Higher ΔPSQI values indicate a worsening of sleep quality.

To assess the association between changes in neural parameters and changes in subjective sleep quality, we performed a multiple linear regression analysis, including baseline PSQI values as a covariate. Diagnostic tests for the multiple linear regression model indicated no violation of model assumptions (Durbin–Watson *P* = 0.704; VIFs = 1.120–1.333). The regression model was statistically significant (*F*(4, 20) = 4.82, *P* = 0.00693) explaining 49% of the variance in sleep quality change (*R* ^2^ = 0.49, adjusted *R* ^2^ = 0.39). The change in GABA levels (i.e. capacity for inhibition) was a significant negative predictor (β* = *0.80, SD = 0.32, *P* = 0.0227), indicating that larger increases in GABA levels were associated with smaller improvements in subjective sleep quality. Change in functional connectivity was a significant positive predictor (β* = *−0.68, SD = 0.31, *P* = 0.0425), implying that larger increases in functional connectivity were linked to greater improvements in sleep quality. The prediction strength of change in SICI (i.e. ability to modulate intracortical inhibition) did not reach significance (β* = *−0.65, SD = 0.31, *P* = 0.0516) but showed a trend towards larger increases in inhibition being associated with larger improvements in sleep quality (Fig. 8). We controlled baseline PSQI levels which did not reach significance (β* = *−0.68, SD = 0.34, *P* = 0.0569), but individuals with poorer sleep quality at baseline tended to show larger improvements in sleep quality.

### Training specificity – resistance training control group

The RT group showed increased explosive strength after training, while the GABAergic system and subjective sleep quality remained unchanged (Table 2), indicating that these effects were specific to balance training rather than general physical activity.

**Table 2:** Paired *t* tests of parameters before *versus* after strength training

Parameter	Mean ± SD PRE	Mean ± SD POST	*t* value	*P* value	Cohen's *d*
**RTD**	163.04 ± 84.90	184.15 ± 74.96	*t*(13) = −2.24	0.0428	−0.60 [−1.16; −0.02]
**PSQI score**	5.78 ± 4.29	5.22 ± 3.69	*t*(17) = 0.57	0.577	0.13 [−0.33; 0.60]
**GABA levels in left motor cortex**	2.11 ± 0.92	2.33 ± 0.44	*t*(13) = −0.79	0.446	−0.21 [−0.74; 0.32]
**SICI**	2.77 ± 10.93	5.75 ± 11.70	*t*(17) = −0.90	0.383	−0.21 [−0.68; 0.26]
**Sensorimotor functional connectivity**	2.80 ± 0.88	3.15 ± 1.06	*t*(15) = −1.10	0.289	−0.27 [−0.77; 0.23]

*Note*: The strength training intervention improved explosive strength, but none of the neural and subjective sleep parameters changed after the intervention.

Abbreviations: PSQI, Pittsburgh Sleep Quality Index; RTD, rate of torque development; SICI, short‐interval intracortical inhibition while balancing assessed in tibialis anterior muscle.

## Discussion

The present study contributes to the search for effective non‐pharmacological strategies to manage sleep problems in older individuals, a population particularly vulnerable to sleep disturbances and the adverse effects of pharmacological treatments. The balance intervention targeted the sensorimotor cortex, a region that has rarely been investigated in sleep studies but that appears to play a role in top‐down sleep regulation and is crucial for motor inhibition during sleep (Krone et al., 2017; Liu & Dan, 2019). The results show that three months of balance training improves subjective sleep quality and is associated with changes in sensorimotor inhibition in the opposite direction to alterations commonly seen in ageing. In line with our hypothesis, GABA levels (MRS measurement) were increased after balance training (Fig. 4*B*) and greater up‐regulation of intracortical inhibition (TMS measurement) was correlated with better sleep quality (Fig. 6*B*). Unexpectedly, greater increases in GABA levels were associated with smaller improvements in sleep quality (Fig. 8). In contrast, greater increases in functional connectivity (fMRI measurement) were linked to greater improvements in sleep quality (Fig. 8). Our findings indicate that balance training may be an effective, non‐pharmacological intervention for improving subjective sleep quality by modulating GABAergic inhibition and restoring functional connectivity within the sensorimotor cortex. Importantly, these effects are specific for balance training and cannot be generalized to physical activity *per se* as we did not see the same changes after three months of resistance training (see Table 2; no adaptations in GABA levels, intracortical inhibition, functional connectivity nor subjective sleep quality after resistance training).

This study provides the first evidence that balance training has the potential to improve subjective sleep quality in older adults (Fig. 3). While studies have demonstrated that various forms of moderate physical activity have beneficial effects on subjective sleep quality in the general population (Chen et al., 2024; Nguyen & Kruse, 2012; Zhou et al., 2024), our study demonstrates the specific benefits of balance training for older adults. Given that improved subjective sleep is associated with enhanced global cognitive function and overall quality of life (Kudrnacova & Kudrnac, 2023; Lin et al., 2024), these findings highlight the potential of balance‐focused interventions as a low‐cost, accessible strategy to support healthy ageing. If we look at the sleep quality of our sample, the baseline score of 6.0 falls within the category of poor sleep quality (Buysse et al., 1989). Yet, this score is relatively better compared with previous studies with older adults that reported higher scores (e.g. Hariprasad et al., 2013; Li et al., 2004; Shum et al., 2014). Nevertheless, the 23.8% reduction in PSQI score observed in this study is greater than the reductions seen after a 6‐month (three training sessions per week) intervention of Tai Chi or a 6‐month daily yoga intervention (Hariprasad et al., 2013; Li et al., 2004). Both Tai Chi and yoga are currently considered to be among the most effective physical activity interventions for subjective sleep quality enhancement (Zhou et al., 2024). The present findings suggest that the balance elements incorporated into Tai Chi and yoga might be a relevant component for the observed sleep improvements after these interventions.

The good sleep quality observed in our sample of older adults was also reflected in their objective sleep architecture (see Table 1). For example, participants in the balance group showed higher sleep efficiency (total sleep time/total time in bed; 84.6% *vs*. 82%), shorter sleep onset latency (12.5 min *vs*. 8 min), and a greater proportion of deep sleep (12.6% *vs*. 9%) compared with mean values reported for the general population of around 71 years of age (Ohayon et al., 2004; Scullin & Bliwise, 2015). These high baseline levels of both subjective and objective sleep parameters may have limited the potential for further improvements, thereby partly explaining the absence of significant changes following the balance training. Furthermore, objective measures of sleep architecture often show minimal or no significant impairments in individuals with insomnia, despite their pronounced subjective sleep complaints (Schneider et al., 2025). Consequently, most interventions find only minimal or no changes in objective sleep measures. For instance, Chen and colleagues (Chen et al., 2024) reported no changes in sleep architecture following a 12‐week combined Tai Chi and strength training programme in both healthy individuals and insomnia patients, while observing improvements in subjective sleep quality. It remains unclear whether sleep architecture could be changed with a longer intervention period.

In line with our hypothesis, balance training increased GABA concentration in the sensorimotor cortex (Fig. 4*B*). GABA levels in the sensorimotor cortex are considered regionally specific and should not be assumed to reflect changes in other brain areas (Greenhouse et al., 2016). However, this specific increase might be important to suppress the activity in the motor system and in brain networks encompassing the sensorimotor cortex when falling asleep (Tarun et al., 2021). Surprisingly, the regression analysis revealed a negative effect of increases in GABA on subjective sleep quality meaning that subjects who had the smallest increase in GABA benefited the most from positive changes in subjective sleep quality (Fig. 8). Although these adaptations seem contradictory at first, one possible explanation is consistent with the two‐layer model of inhibition introduced by Taube & Lauber (2024). Based on this model, the increase in GABA levels may indeed enhance the capacity for inhibition, whereas improvements in sleep may depend more strongly on the functional modulation of inhibition. In the current study, we assessed the modulation of inhibition using SICI while subjects performed a balance task and while they fell asleep during an afternoon nap (Fig. 5). Consistent with previous findings, higher SICI (i.e. higher intracortical inhibition) during balance tasks was positively correlated with better balance performance (Kuhn et al., 2024) (Fig. 6; Mouthon & Taube, 2019*A*). The present study additionally revealed a significant correlation between changes in intracortical inhibition during sleep and improvements in sleep quality, providing new insights into the relationship between GABAergic inhibition and sleep quality (Fig. 6*B*). Specifically, participants who showed the greatest upregulation of intracortical inhibition during sleep exhibited the most substantial improvements in subjective sleep scores from PRE to POST.

In this study, SICI was assessed during an afternoon nap to capture intracortical inhibition around sleep onset. Although sleep onset occurs under different conditions during daytime naps *versus* nocturnal sleep, given a more sleep‐favourable circadian phase, higher homeostatic pressure and potentially a lower arousal threshold in the evening, available evidence indicates that SICI is relatively stable across time of day and levels of sleepiness under non‐sleep‐deprived conditions (De Gennaro et al., 2007; Doeltgen & Ridding, 2010; Manganotti et al., 2001). Moreover, studies assessing SICI across sleep stages report broadly comparable stage‐dependent modulation across nocturnal sleep and daytime sleep, with stronger inhibition observed during deeper NREM sleep relative to lighter sleep stages and wake (Avesani et al., 2008; Salih et al., 2005). Taken together, these findings support the use of nap‐based sleep onset SICI as a reasonable proxy for inhibitory mechanisms relevant to nocturnal sleep onset.

Contrary to expectations, there was no significant increase in SICI in the balance group compared with the control group during balancing nor during sleep (Fig. 5). Unfortunately, only 26 participants were able to sleep during the afternoon nap and keep head movement within acceptable limits for TMS during both PRE and POST measurements. Thus, the number of data points is limited for SICI during sleep. Although this analysis was planned *a priori* and directly addressed our primary hypothesis, the reduced number of participants for whom SICI could be reliably obtained during sleep limits statistical power. Consequently, these findings should be interpreted cautiously and considered suggestive rather than conclusive, pending replication in larger samples. Concerning the measurements during the balance task, there is substantial literature showing significant increases in SICI during a balance task after balance training (Kuhn et al., 2024; Mouthon & Taube, 2019; Taube et al., 2020). In line with this, the participants in our study undergoing balance training demonstrated a 44% increase in inhibition while the control group showed a decline of about 16%. The absence of a significant change in SICI might therefore be explained by the high variability of inhibition in the current sample. Moreover, it is well established that older adults typically require more time to adapt to training interventions. In the study by Kuhn and colleagues (Kuhn et al., 2024), the most pronounced changes in SICI were observed only after six months of balance training. Therefore, the three‐month training period used in the present study may have been insufficient to elicit consistent upregulation of SICI across all participants of the training group. It is plausible that, as an initial step, participants first need to increase overall GABA levels to enhance their capacity for inhibition. Then, as a second and potentially longer process, they learn to utilize this increased inhibitory capacity in a functionally effective way. This may help explain the negative association observed between GABA levels and PSQI scores. Participants with lower GABA levels at baseline and bigger increases from PRE to POST may not have had sufficient time for functional adaptation. This interpretation is supported by the finding that individuals with lower initial GABA levels exhibited greater increases in GABA over the training period. A longer intervention, such as six months, might consequently have been more effective in consistently enhancing intracortical inhibition in older adults. Nonetheless, the observed correlation between individual changes in inhibition during light sleep and subjective sleep quality (Fig. 6*B*) may imply a modulatory role for SICI (e.g. intracortical inhibition) during sleep in driving the observed effects on subjective sleep quality.

After balance training, the strength of functional connectivity within the sensorimotor network was significantly increased (Fig. 7*B*). Very often, age‐related increases in functional connectivity across hemispheres are assumed to represent a compensatory neural mechanism or are interpreted as a sign of neural dedifferentiation (Cabeza, 2002; Heise et al., 2022). However, the more intense communication of left and right sensorimotor cortices in the present study might be caused by the need to coordinate the left and right body side simultaneously while balancing. As an illustration, playing instruments that require independent motor actions of the left and right hand, such as stringed instruments, fosters interhemispheric inhibition and therefore supports lateralized brain activity, whereas instruments requiring cooperative actions of both hands, such as the piano, foster interhemispheric communication (Vollmann et al., 2014). This increased collaboration of left and right sensorimotor cortices may also help to suppress motor activity simultaneously on both sides to fall asleep. In line with this, Blanco‐Hinojo and colleagues (Blanco‐Hinojo et al., 2021) state that functional coupling of brain areas is not only important for states of activity but also when inhibiting the brain in order to sleep. In their study they demonstrate that alprazolam, a benzodiazepine drug binding to specific sites on GABA_A_ receptors and thereby increasing the receptor's responsiveness to GABA, increases functional coupling of the sensorimotor cortices. At the same time, alprazolam significantly reduces subjective arousal compared with placebo. In our study, increased functional connectivity significantly predicted an improvement in subjective sleep quality (Fig. 8). The results of a study investigating the effects of a combined Tai Chi and strength training intervention on sleeping behaviour in insomnia patients point in the same direction (Chen et al., 2024). After training, insomnia patients reported better sleep and demonstrated increased functional connectivity between left and right sensorimotor cortices. In their discussion, the authors describe their finding of reduced connectivity in insomnia patients compared with healthy controls as contradictory to the widely accepted hyperarousal model of insomnia (Dressle & Riemann, 2023; Riemann et al., 2010). However, we argue that brain dedifferentiation and hyperarousal may both contribute to the decreased efficiency in sleep–wake regulation in older adults. Brain dedifferentiation reflects less coordinated activity and inefficient communication between brain regions within the same network. As a result, older adults show reduced distinctiveness in neural representations, which may derive from reduced inhibition (Cuypers et al., 2020; Taube & Lauber, 2025). In this context, the observed increase of sensorimotor network functional connectivity may be another effect of enhanced local GABA levels, helping to modulate activity simultaneously within both hemispheres, in line with the above‐mentioned study with benzodiazepine medication (Blanco‐Hinojo et al., 2021). Together with increased cortical GABAergic inhibition, enhanced sensorimotor network functional connectivity may therefore be important for efficient bilateral suppression of motor activity during sleep (Liu & Dan, 2019). Thus, our findings suggest that stronger functional connectivity within the sensorimotor network reflects enhanced neural specialization, which may contribute to more efficient sleep–wake regulation in older adults.

Our result of improved subjective sleep quality adds to the list of health benefits associated with balance training for older adults. Previous research has shown that balance training reduces the risk of falls and decreases the fear of falling, improves cognitive abilities including memory and spatial cognition and elevates quality of life in various domains such as well‐being, psychological status and social interaction (Duque et al., 2013; Madureira et al., 2010; Rogge et al., 2017; Sherrington et al., 2011). Together with the observed improvement in subjective sleep quality, balance training is a valuable practice to integrate into the everyday lives of older adults.

The present study is not only important with respect to the beneficial behavioural consequences on sleep behaviour after balance training, but it also adds to the understanding of the underlying mechanisms responsible for the improvement in sleep quality. Furthermore, our study contributes to better understanding the role of the sensorimotor cortex as a part of the sleep network, responsible for suppressing motor activity during sleep. Understanding the specific neural adaptations induced by balance training and distinguishing them from those elicited by other forms of physical activity (for example, resistance training; see Table 2) allows for the development of more targeted and population‐specific interventions.

This study shows improved subjective sleep quality after three months of balance training in older adults, alongside changes in inhibitory markers and sensorimotor network coupling. However, there are certain limitations which need to be taken into consideration. (1) A longer training intervention may be necessary for individuals with low inhibitory capacity to increase their GABA levels and learn to use them effectively, as reflected in increased SICI. (2) We could not measure GABA levels in the hypothalamus or thalamus, both being a vital part of the GABAergic inhibition for sleep initiation and maintenance (Oishi et al., 2023; Saper & Fuller, 2017). With novel (e.g. whole brain MRS) methodology this would be an interesting addition to the current analysis of mechanisms, to assess whether the change in sensorimotor GABA levels also increases the GABA levels in other brain areas involved in sleep regulation. (3) Furthermore, the ability to modulate GABAergic inhibition (i.e. SICI) can non‐invasively only be assessed in the primary motor cortex by means of paired‐pulse TMS. While GABAergic inhibition in the motor cortex has been shown to be modulated during sleep (Avesani et al., 2008; Salih et al., 2005), a measure of subcortical GABAergic inhibition is technically not possible, so we cannot exclude subcortical GABAergic contributions to the improved subjective sleep quality. (4) The paired‐pulse TMS acquisition and analysis were not explicitly blinded to group allocation. However, as the outcomes were objective neurophysiological measures obtained using predefined stimulation parameters, the absence of blinding is unlikely to have influenced the results. (5) Furthermore, the control group was passive and not supervised during the intervention period. However, self‐reported exercise frequency, duration and activity type remained stable from PRE to POST. Additionally, a separate strength‐training comparison group did not show similar changes, suggesting that the observed effects are unlikely to be attributed to non‐specific engagement alone.

Future research should investigate balance training as a potential intervention for clinical populations with impaired GABAergic inhibition and disrupted within‐network functional connectivity. Such impairments are not only characteristic of healthy ageing but are also prevalent in many pathological conditions (Taube & Lauber, 2024). For example, reduced inhibitory activity has been linked to pain perception, depression and neurodegenerative disorders like mild cognitive impairment or Alzheimer's disease (Bi et al., 2020; Jodoin et al., 2020; Luscher & Fuchs, 2015), and conditions like multiple sclerosis and Parkinson's disease are associated with impaired sensorimotor functional connectivity (Caspers et al., 2021; Rocca et al., 2018).

In conclusion, three months of balance training in older adults led to improved subjective sleep quality going along with increased GABA levels in the sensorimotor cortex and enhanced functional coupling of left and right sensorimotor cortices. We assume that these changes indicate recovery of sensorimotor brain coupling to bilaterally coordinate motor activity during balancing but also motor suppression during sleep. Further, we propose that balance training could serve as an effective and safe alternative to pharmacological treatments for positively modulating the GABAergic system. This finding is particularly relevant for older adults, as they often experience sleep problems and typically show impaired within‐network connectivity and overactivation based on impairments in GABAergic inhibition.

## Additional information

## Competing interests

The authors declare no competing interests.

## Author contributions

Conceptualization: W.T., L.X., B.L. Methodology: all authors. Investigation: SE, S.S., X.L. Formal analysis: S.S., X.L. Visualization: S.S., X.L. Writing – original draft: S.S. Writing – review & editing: all authors. All authors have approved the final version of the manuscript and agreed to be accountable for all aspects of the work. All persons designated as authors qualify for authorship, and all those who qualify for authorship are listed.

## Funding

This research was funded by the Swiss National Science Foundation (SNSF), grant number 32003B_197687.

## Generative AI statement

During the preparation of this work, the authors used Microsoft 365 Copilot (GPT‐5 chat model) to assist in refining the manuscript by improving flow and readability within paragraphs. No original content was generated by GAI and all GAI‐assisted text was reviewed and revised by the authors to ensure accuracy and clarity.

## Supporting information


Peer Review History

## Data Availability

The dataset analysed and the R script used for analysis are available online in Open Science Framework (OSF) at https://osf.io/t75vc/.

## Detailed balance training plan

**Table:**

Exercises: week 1	Organization	Time
**Lesson 1: Monday – Tuesday**		
Presentation round: first name, profession, hobby, qualifying adjective	*In plenum*	10'
Warm up: ankle – knee – hip Short game: 1 – 2 – 3 (2 = touch the knee, 3 = raise the thumb)	*In plenum*	10'
‐ Move the weight forward then backward: pay attention to the core ‐ Rise on your toes, then lower on your heels	*In plenum*	5'
Introduction of positions: one pushes the other a little 1) Natural position, each as he feels 2) Legs apart but at the same height 3) Feet tight 4) Tandem feet 5) What is the best position: 3/4 6) Turn on themselves: take the 3/4 position	By two	10'
Core stability introduction: Lying on the back, core engaged: the other raises the legs Standing up, core engaged, let yourself fall back: the other one catches you	By two	5'
**Lesson 2: Wednesday**		
Throw a ball to one of the people by saying their first name, the person with the ball introduces themselves: first name, profession, hobby, qualifying adjective	*In plenum*	10'
Warm up: ankle – knee – hip Mirror	*In plenum*	10'
1) Natural position, each as he feels 2) Legs apart but at the same height 3) Feet tight 4) Tandem feet 5) What is the best position: 3/4 During these positions: turn the arms then turn the upper body (first without the head, then with the head)	*In plenum*	10'
Introduction to the one‐footed position 1) On one foot without moving 2) On one foot and the other forward and backward 3) Plane 4) Little ball	*In plenum*	5'
Little fight in all positions seen		10'
**Lesson 3: Thursday – Friday**		
Tandem position: Throw the ball to one of the people by saying their first name, the person with the ball introduces themselves: first name, profession, hobby, qualifying adjective	*In plenum*	10'
Warm‐up: ankle – knee – hip Resume positions on one foot ½ turn	*In plenum*	10'
− In pairs: eyes closed: one press turn to the left or right, two presses stop and stand on the foot on the side of the pressures − Turn the person around (eyes closed), stop then the person tries to walk straight ahead (eyes open) − ½ turn	By two	10'
Natural position, each as he feels Legs apart but at the same height Feet tight Tandem feet What is the best position: 3/4 On one foot All positions, eyes closed.	*In plenum*	10'
Little fight on one foot If there is still time, a little fight on the different ones with your eyes closed (ATTENTION) only one that moves	*In plenum*	5'
Exercises: week 2	Organization	Time
**Lesson 4: Monday – Tuesday**		
Throw a ball to one of the people by saying their first name, the person with the ball introduces themselves: first name, profession, hobby, qualifying adjective – possibly on one foot or in tandem	*In plenum*	10'
Standard warm‐up +: ° Go down to the knees then come back up and jump ° Giant steps (lunge) ° One throws a tennis ball and the other tries to catch it with a cone (open side): movement ° Review position on one foot	*In plenum*	10'
− One advances in the room with his eyes closed and the other guides him only with taps on the shoulder − Turn the person around then stand on one foot	By two	10'
− Face to face: try to hold the ball between the knees of the partners, one the right leg, the other the left then hold the ball between the feet, then between the feet of both partners	By two	10'
Two people hold a rope and the third person climbs over it on one foot Small jump over the rope and land on one foot On one foot make a ¼ turn then ½ turn Hopscotch game	*In plenum* or group	15'
− Little fight on one foot with theraband	By two	5'
**Lesson 5: Wednesday**		
Throw a ball to one of the people by saying their first name, the person with the ball introduces themselves: first name, profession, hobby, qualifying adjective – possibly on one foot or in tandem	*In plenum*	10'
Standard warm‐up +: − Pantin (shift leg and arm) − On one foot and rotate the other around the body − Make the plane then the little ball	*In plenum*	10'
Warm‐up sequence: leaf – stone – scissors (or chifoumi) − The one who loses runs and the other does squats during the time (on one foot)	By two	10'
One stands on two feet, eyes open at first, the partner taps on the shoulder and the person must make a quarter turn to the side of the tap. Once you understand the exercise, same thing but with your eyes closed. One stands on two feet, eyes open at first and holds the rope, the other tries to unbalance him ATTENTION: not a fight only a person who moves − Eyes closed, two feet − Eyes closed, feet together − Eyes closed, tandem (if possible otherwise only hold in tandem with eyes closed)	By two	10'
Face to face: try to hold the ball between the knees of the partners, one the right leg, the other the left then hold the ball between the feet, then between the feet of both partners	By two	5'
Reflex: one holds the ball up then lets it go, the other with his hands behind his back must try to catch it Variations: change the positions of the feet (one foot, tandem, etc.)	By two	5'
**Lesson 6: Thursday – Friday**		
Throw a ball to one of the people by saying their first name, the person with the ball introduces themselves: first name, profession, hobby, qualifying adjective – possibly on one foot or in tandem	*In plenum*	10'
Standard warm‐up +: ° Make the puppet (shift legs and arms) ° Mirror with me ° Leg grab: trying to touch the other's thigh ° Theraband: take both ends of a theraband in your hands and place one foot on the loop then push down ° Theraband: hold one side in one hand and the other under the foot then with the remaining foot push to the side	*In plenum*	15'
Make a hedge with the therabands and everyone passes over them, each time standing on one foot for 2 seconds Little jump over the rope as quickly as possible and when I clap my hands, get on one foot Hopscotch Pass the theraband (folded in 3 or 4) under the foot	*In plenum*	15'
Reflex plus U‐turn: one throws the ball, the other closes his eyes: the one who throws the ball says great then throws the other opens his eyes and must catch the ball same thing except that instead of closing his eyes, he turns his back and has to stand on top	By two	10'
Exercises: week 3	Organization	Time
**Lesson 7: Monday – Tuesday**		
Zip game – Zap **Rules**: the players are arranged in a circle, around the leader (a facilitator at the start of the game). The leader points to a player by saying ‘Zip!’. The designated player must, as quickly as possible, give the first name of his neighbour on the right. Then the neighbour on the right must clap their hands and stand on one foot, which creates a cascade around the circle until the player designated at the base is also on one foot. If the leader says ‘Zap!’, he must give the first name of his neighbour on the left and the waterfall goes to the left. When the leader says ‘Zip Zap!’, all the players change places and form a circle again, making sure that they no longer have the same neighbours. If a player makes a mistake or does not stand on one foot, he takes the place of the leader until another player makes a mistake.	*In plenum*	10–15’
Warm‐up: standard +: 1 2 3 Sun	*In plenum*	10'
1. In pairs: stand on one foot then throw the hoop and catch it with your hands 2. In pairs: one throws the small hoop and the other must catch it with their foot 3. Eyes open: in line on one foot: pass the hoop with your foot 4. Hold the small hoop and pass your leg through it then take your foot out 5. Place the hoop on the foot then bring the foot back while keeping the hoop hanging ⇨ Increased difficulty: on a balance cushion	In pairs and in groups	20'
A column: all on one foot: then pass a ball to each other from the sides from the front, the last one, once the ball is in hand, runs ahead and starts again. If there are enough people, two teams then in the form of a race and otherwise a column and in the form of training	*In plenum*	5'
Final game: orbit (stand in a circle and hold hands then pass a large hoop around the circle.		5'
**Lesson 8: Wednesday – Thursday**		
Standard warm‐up +: − With a ball: make an 8 between the legs − Standing: raise your leg and pass the ball underneath − On one foot: eyes open and closed head tilted back − Move quickly on a line and when I clap my hands, stand on one foot − In pairs: not chased and throwing a ball at the same time	*In plenum*	15'
Exercise repetition: throw the hoop then catch with your foot Getting started: in the form of running: in line: passing the hoop with your foot	Two teams	10'
1) Stand on one foot, pick up a ball (small ‐ large ‐ heavy, etc.) according to each person's possibilities and bring it above the head, still on one foot 2) Standing on one foot, bring the free leg towards you, place the ball on the foot and keep it without dropping it 3) Standing on one foot, place the free leg at 90°, then make circles with the ball around the thigh (ball in the hands). 4) 5 people each hold a small hoop in their hands and form a line: the others go through the hoops, putting one foot in each time. Or else 1) Back to back, on one leg, pass a ball over your head 2) Back to back, on one leg (or two if too difficult), pass the ball over your head, then between your legs		
3) Back to back, on one leg (or in tandem?), pass a ball to the sides 4) Five people each hold a small hoop in their hands and form a line: the others go through the hoops, putting one foot in each time.		
One of the team stands at the bottom ready to catch the hoop with their foot. The next person runs, throws the hoop which the first must catch with their foot then takes their place. The first one gets in line, etc. The team that catches the most hoops wins.	Two teams	
Exercises: week 4	Organization	Time
**Lesson 9: Tuesday – Wednesday**		
Standard warm‐up + In pairs: on one foot: foot against foot: try to unbalance the other (gently) Little fight on one foot with the therabands	*In plenum* By two	10'
Stand facing your partner, placing a long necklace (hooked on the rings) halfway between the two of you. Bend your legs slightly, hands on your thighs. From this starting position, a third partner will give you key words (for example: head). You will then have to touch your head with both hands. If the keyword is ‘knees’, then you will need to touch your knees with both hands. As long as your partner tells you which body parts to touch, you will have to touch them. On the other hand, from the moment he says the word ‘long necklace’, you will have to be the quickest to grab the long necklace hooked to the rings. => to do on one foot	By three	10'
**Unstable surface: 16 stack and large stack** 1) Stand on one foot then close your eyes ⇨ Leg catcher ⇨ Then repeat the exercise of standing on one foot then closing your eyes 2) In the form of a race: passing the small hoop with your foot on the large mat 3) Work the tandem position on a large mat or 16 mat: rotate the arms + rotate the upper body 4) Little fight on one foot with theraband Additional difficulties: close your eyes	*In plenum*	20'
**Balance in motion: bench introduction** − Walk on the bench right side up (just for introduction) ***Upside down benches*** − To walk − Walk, stop in the middle and stand on one foot, then continue − Walk, stop in the middle and make a complete turn	*In plenum*: take the time	10‐15'
Final: sheathing circle: in a circle numbered 1 and 2: the body well stretched and sheathed, the 1s tilt forward and the 2s backwards all at the same time.	*In plenum*	5‐10'
**Lesson 10: Thursday – Friday**		
Standard warm‐up + 1 – 2 – 3 small goldfish (or 1 – 2 – 3 sun) ⇨ 2nd round stop on one foot ⇨ 3rd round stop on one foot, eyes closed	*In plenum*	10'
**Unstable surface: 16 stack and large stack** **1)** Get on one foot: upper body rotation **2)** On one foot: raise the leg, ¼ rotation, straighten the leg and bring it behind **3)** Plane => small ball Additional difficulties: close your eyes	*In plenum*	10'
**Upside down benches** **1)** Stand on one foot along the length of the bench **2)** Get on one foot: upper body rotation **3)** On one foot: raise the leg, ¼ rotation, straighten the leg and bring it behind **4)** Plane => small ball **5)** Stand on the bench (perpendicular to the surface), if possible on one foot **6)** In pairs: back to back on a bench: pass the ball from above	*In plenum*	20'
Two benches upside down: face to face (1.5 m) Position yourself on the bench (on two feet) perpendicular to the surface, one part on one bench, the other on the other: quick pass with a ball ⇨ Additional difficulty: add a smaller ball	*In plenum*	10'
End game: day/night	*In plenum*	5'
Exercises: 5	Organization	Time
**Lesson 11: Monday – Tuesday**		
Standard warm‐up +Contacting swissball − Tilt the pelvis forwards and backwards then to the sides, then rotation − Move forward while sitting on the swissball (small jump) − Sit: raise one foot then the other − In pairs back to back with a swissball in between: go down and up (squats)	*In plenum*	15'
Game: Modified burnt ball: the running team starts, those in the middle run to the bench upside down (one of the two sides) and all stand on it, when everyone is on it (without falling), the person running must be on a base otherwise they get burned.	*In plenum*	10'
**Unstable surfaces**Large mat – mat of 16 − Run in place then at the stop sign get on one foot − Stand on one foot and tilt your head back − Walk with your eyes closed on the big carpets (in pairs)	*In plenum*	10'
**Upside down benches**: ‐ Walk on the bench over obstacles (some participants hold a stick and adjust the height) ‐ Step on the bench and in the middle, squat ⇨ Additional difficulties: teapot ‐ No giants ‐ In pairs: face to face: try to hold a small ball between the foot then between the knees ‐ In pairs: small fights on two feet ⇨ Additional difficulties: on one foot (if too great a difference in level between two, one on one foot and the other on both.	*In plenum*	20'
Final challenge: try to cross each other on the bench upside down	By two	5'
**Lesson 12: Wednesday**		
Standard warm‐up + ‐ Sitting on the swissball: one throws the small hoop the other must catch it with one foot sitting on the swissball (the one throwing is standing) ‐ Swissball: try to sit without your feet on the ground	*In plenum*	15'
**From the bench upside down**: ‐ Jump on a 16 mat with balanced landing (stable) ‐ Same with ¼ ‐ ½ or 1 full turn ‐ Same thing with landing on one foot ‐ Same thing with eyes closed (on two feet and if too easy on one foot)	*In plenum*	15'
**Upside down benches**:‐ From a top of a box: jump onto the bench upside down‐ Two benches facing each other: each on a bench: badminton ‐ Moving in a shuffled step on the bench	*In plenum*	15'
Mikado: two upside down benches: at the end a box with two mikados ‐ Go to the bench and take a mikado, get off the bench, run around a pole and return to the starting line. When the first has got off the box, the second can leave. The team with the most sticks wins.	*In plenum*	10'
**Lesson 13: Thursday – Friday**		
Standard warm‐up + sit on a swissball without your feet	By two	15'
Stand facing your partner, placing a long necklace (hooked on the rings) halfway between the two of you. Bend your legs slightly, hands on your thighs. From this starting position, a third partner will give you key words (for example: head). You will then have to touch your head with both hands. If the keyword is ‘knees’, then you will need to touch your knees with both hands. As long as your partner tells you which body parts to touch, you will have to touch them. On the other hand, from the moment he says the word ‘long necklace’, you will have to be the quickest to grab the long necklace hooked to the rings. => to do on a bench then on one foot on the bench	By three	10'
Upside down bench ‐ Bench – box – bench – box – bench: be careful to secure the bench that goes up and down either with a top of the box or against the wall ⇨ Normal passage ⇨ Stop in the middle: make a half or full turn ⇨ Get on one foot	*In plenum*	15'
Upside down bench‐ One on the bench: the other on the ground throws him a ball but causes movements‐ Standing on the bench => Theraband: take both ends of a theraband in your hands and place one foot on the loop then push down => Theraband: hold one side in one hand and the other under the foot then with the remaining foot push to the side => Hold both ends of the theraband in your hands and pass your leg over them	By two	15'
Finale: little fight on the benches with therabands	By two	10'
Exercises: 6	Organization	Time
**Lesson 15: Monday – Tuesday**		
Standard warm‐up + ‐ Pedalo ‐ Small in‐line rocking board (first offset then aligned) ‐ Swissball	*In plenum* / in small groups	15'
1 – 2 – 3 sun sitting on the swissball	*In plenum*	5‐10'
Unstable surface: 16 mat or large mat ‐ One on the unstable surface must catch with their foot the small hoop thrown by the other	By two	5'
Upside down bench‐ Start by simply walking on it, stop in the middle, stand on one foot, go down‐ Walk while passing yourself with a small ball‐ Walk with your leg raised and pass the ball‐ Same thing as on the unstable surface: one throws the small hoop, the other must catch it with their foot‐ Online passage of the small hoop from one foot to the other (if works well in running form) => repeat before once on the ground	*In plenum*	15'
Two benches one behind the other: participants pass over the two benches upside down at the end of the benches, they must take a hoop and throw it at one of the three stakes (1 stake = 1 point / 2nd = 2 points / 3rd = 3 points)	By team	10'
Swissball: sitting and lying on your stomach		10'
**Lesson 16: Wednesday**		
Standard warm‐up + ‐ Pedalo ‐ Mexican hat – roll – rolabola ‐ Swiss ball	*In plenum*	15'
Estafette: two teams: 3 poles – half of the group on one side, the other on the other side ‐ Run to the middle pole, turn around 4 times then continue to the next pole to hand over to the next one	By teams	10'
Upside‐down bench on the ground in front of the rings, bar fixed on the rings: sitting on the bar, start from a box and land on an upside‐down bench: ‐ Direction of length in relation to the rings: advance to the end of the bench ‐ Direction of width (vertical) in relation to the rings Try without holding on to the rings	In two groups Alternation with the next block	10'
Benches backwards: ‐ Passage on the benches, eyes closed ‐ Several benches upside‐down position parallel: go over the series of benches, stopping on each bench (spread the benches apart to increase the difficulty) ‐ Rocker (bench on top of box)	In two groups Alternation with the front block	10'
Swissball on your knees		10'
Handkerchief: in a circle all on one foot (with one then with two handkerchiefs)	*In plenum*	5'
**Lesson 17: Thursday – Friday**		
Standard warm‐up + ‐ Pedalo ‐ Mexican hat – roll – rolabola ‐ Swissball ‐ Introduction to small carpets (getting pulled)		20'
Tail catcher: jumper hung in training		10'
1) Benches attached to the wall bars: stand on two small carpets and descend (firstly, descend seated) 2) Flying carpet (top of box with basketball underneath)	Two groups Alternating 1 and 2	10'
Reverse benches: reflex exercise ‐ In pairs: one lets go of the stick (with one or two), the other must catch it with their hands behind their back. ‐ In pairs: one closes their eyes, the other says great then throws a small (soft) ball		10'
Upside down bench ‐ Half turn with feet apart ‐ Not chased ‐ Face to face on the bench, palm to palm, a complete turn and we join palm to palm		10'
Two parallel benches, each team on a bench in a column: passing the ball from above, then from the side. The ball starts in front and finishes behind, the last one gets off the bench and runs to stand in front. First team to finish wins	By team	5'
Exercises: 7	Organization	Time
**Lesson 18: Monday – Tuesday**		
Standard warm‐up + Swissball strengthening ‐ On one foot, roll the swissball around you ‐ In pairs back to back, wedge the swissball between the two backs then lower into the knees => for those who manage to sit on the ground and get up	*In plenum*	20'
Handkerchief: those in the circle stand on one foot	*In plenum*	10'
Swissball: ‐ Sat ⇨ Sit on the swissball then put your feet on a volleyball or basketball ⇨ In threes: sit on the swissball then place your feet on the swissball of the person in front (whose feet are on the ground). The third person for belaying. ⇨ Facing each other: passing with a ball (without putting your feet on the ground) ⇨ Small fights with theraband: one with his feet on the ground and not the other ⇨ Then the two without their feet on the ground	By two or three	25'
Swissball: bench ‐ Walk on the upside‐down bench, holding the swissball at arm's length above your head ‐ Stand on one foot and place the swissball to the right then to the left ‐ Same as the warm‐up: back to back, swissball stuck between the two backs and down into the knees	*In plenum*	10'
‐ Lying down: let go of one hand then the other then both ‐ Kneeling		10'
Final challenge: WARNING: difficult, only those who want, others can continue seated	*In plenum*	5'
**Lesson 19: Wednesday**		
Standard warm‐up + Balance mat on one leg	*In plenum*	10'
Game: tic tac toe (hoop + cone)	By team	10'
Bench backwards:‐ Hold a small ball between the knees then between the feet‐ Standing on the bench => Theraband: take both ends of a theraband in your hands and place one foot on the loop then push down => Theraband: hold one side in one hand and the other under the foot then with the remaining foot push to the side => Hold both ends of the theraband in your hands and pass your leg over them‐ Walk on the upside‐down bench, holding the swissball at arm's length above your head‐ Stand on one foot and place the swissball to the right then to the left‐ Same as the warm‐up: back to back, swissball stuck between the two backs and down into the knees	By two *In plenum*	20'
‐ First sit on the bar in the middle of the rings in balance, if stable swing slightly ‐ Same thing but swing harder (put big stacks under)	*In plenum*	10'
‐ Badminton on the benches		10'
Orbit on the benches in racing form	By team	5'
**Lesson 20: Thursday – Friday**		
Warm‐up + ‐ Mexican hat ‐ Rolabola ‐ Pedalo ‐ Swissball	*In plenum*	10'
Upside‐down bench ‐ Spin sumo on the bench ‐ Not chased ‐ In pairs: one throws the ball and the other catches it ⇨ First with the hands then with the foot ‐ In pairs: one throws a hoop and the other tries to catch it with their foot ‐ Jump from the top of the box onto the bench	*In plenum*	10'
Estafette memory: run over a large mat, then on the ground before getting on an upside‐down bench and turning over two memory cards (if family I take, otherwise I leave)	As a team	10'
Swing from the rings and land on a balance cushion or a 16 mat or a large mat: on two feet then on one foot	*In plenum*	10'
Challenge: make a circle all seated on the swissball and put their feet on the ball of the one in front (no one must have their feet on the ground)	*In plenum*	10'
Exercises: 8	Organization	Time
**Lesson 21: Monday –Tuesday**		
Standard warm‐up + in pairs: passing a ball to each other while moving in a shuffle (one leads, the other follows) + Swissball: sit on the ball and move forward making small hops + Swissball: sit on it	*In plenum*	20'
Game: Horse racing: two teams: one circle inside and the other outside: the inside team stands on one foot passes a volleyball meanwhile, the team on the outside runs around the circle three times! The team that has completed the most turns with the balloons is the winner! (ATTENTION: part of the warm‐up, so easy running not sprinting)	By team	10'
Training: Take the different elements of Wednesday's course over the two days and train them **Monday: 1 – 6 ‐ 7** **Tuesday: 3 – 5 – 8**	Per station: 7' per station per group	20'
Challenge: make a circle all seated on the swissball and put their feet on the ball of the one in front (no one must have their feet on the ground) Monday: just in twos or threes Tuesday: a full circle (two teams): one team performs, the other team safeguards	By team (plenum)	10'
**Lesson 22: Wednesday – Thursday evening**		
Standard warm‐up + tandem position: turn the upper body + position on one foot: turn the upper body		10'
Scrabble game: the team stays around a ‘table’ and one after the other runs to look for a letter (one letter at a time). Meanwhile the rest of the team tries to make as many words as possible.		10'
Course: 1) Start: rings with bar in the middle landing on the bench upside down, lengthwise – walk along the entire bench 2) Slalom between the poles 3) Bench – box – bench attached to the wall bars – box – two benches attached to the wall bars large mats underneath (walk on it – go down like skiing) 4) Swissball: sit on the ball and move forward making small hops 5) Reverse bench: move forward and the other passes with the ball 6) Pedalo 7) Go over three boxes (not at the top and quite close) then jump onto a large carpet 8) Passing hurdles: while walking and always take a break on one foot between two	The entire route is done in pairs	30'
Game: reflex circle		5'
**Lesson 23: Thursday morning – Friday**		
Standard warm‐up + Reflex game: ‐ One stands behind him, the other throws and says great when he throws the little ball, the first must catch the little ball ‐ One stands on one foot, eyes closed, the other throws and says great as he throws the small ball, the first must catch the ball	*In plenum*	15'
Estafette: two teams: passage on a bench, bench fixed to the box (not too stiff), on the box pieces of paper with numbers. Everyone must run on the course to turn a number, remember the number, at the end the whole team calculates the sum of all the numbers. Once the sum is done, the team shouts out the number.	By team Prepare the numbers on small pieces of paper	10'
Benches backwards: ‐ One sits on the bench and tries to return the volleyball thrown by the second with his foot. ‐ Badminton on the benches (facing each other) both lie on the bench ‐ One stands on two feet (if too easy then on one foot), eyes closed, the other throws and says top as he throws the little ball, the first must catch the ball ‐ Passing the small hoop over the foot	*In plenum*	20'
Race: two teams or more if many people in a column on an upside‐down bench ‐ Passing the ball from above from the front, the last one comes down from the bench running in front, gets back on the bench and passes the ball again (all the others must take a step back to make room): the first team whose members are all went ahead and won	By team	10'
Exercises: week 9	Organization	Time
**Lesson 24: Monday and Tuesday**		
Standard warm‐up + small rolled green mat: on one foot (eyes open/closed) – tandem position: turn the upper body		10'
Upside‐down benches ‐ One on the ground throws a small ball and on the bench the other must catch it with a cone ‐ One on the ground throws a volleyball and the other on the bench must throw it with his foot ‐ Badminton	By three	20'
Scrabble: upside down bench + table box (one after class looks for a letter, the others try to make as many points as possible with words (no first name or city name, etc.)	3–4 teams	5'
Stand facing your partner, placing a long necklace (hooked on the rings) halfway between the two of you. Bend your legs slightly, hands on your thighs. From this starting position, a third partner will give you keywords (for example: head). You will then have to touch your head with both hands. If the keyword is ‘knees’, then you will need to touch your knees with both hands. As long as your partner tells you which body parts to touch, you will have to touch them. On the other hand, from the moment he says the word ‘long necklace’, you will have to be the quickest to grab the long necklace hooked to the rings. => on the benches	By 3	10'
Bar on the rings: one side of the bench in the place fixed on the bar: up‐down	By group on the ring slopes	10'
**Lesson 25: Wednesday and Friday (Thursday: ascent)**		
Standard warm‐up+ swissball:‐ On one foot + tandem position: place the ball on R then on L‐ On one foot + tandem position: turn‐ Sitting position => lift one foot then the other => put one foot on the other knee => without feet	Take some time with the swissballs	20'
Bench on unstable surface ‐ Bar on the rings: one side of the bench in the place fixed on the bar: up‐down ‐ Bench upside down on one side on a small mat ‐ Bench upside down on a 16 mat ⇨ Cross the benches, make a complete turn, stand on one foot	*In plenum* (three alternating groups) = 7' per station	25'
Game: Pass the ball from above from the front, the last course in front and passes the ball again and so on: in the form of a race	*In plenum*	10'
If you still have time, take up swissball for a while but take it easy	*In plenum*	5'
Exercises: 10	Organization	Time
**Lesson 26: Monday and Wednesday**		
Standard warm‐up + Large rug: ⇨ Run in place then at my signal get on one foot ⇨ Thigh catcher ⇨ medicine ball: hold on to it (WARNING in pairs: especially when going up)	*In plenum*	20'
Warm‐up: 1 – 2 – 3 sun: stand on one foot without moving	*In plenum*	10'
To begin with: ‐ Bar on the rings: one side of the bench in the place fix on the bar: go up‐down: put them at ease ‐ In the form of a competition (NOT THE SPEED THAT COUNTS BUT THE NUMBER OF BALLS IN THE BOX AND THE NUMBER OF PINS AT THE BOTTOM) ⇨ Two teams: short race on a short stretch, climb on the bench, aim with a ball in a box (without the top) then go back down (WARNING: the goal is not to go fast but to aim well without holding on to the ropes of the rings) the team having put the most balls in the box wins (2–3 balls per person depending on the number of participants) ⇨ Bench upside down on a mat of 16 (or only one side above): shot with a volleyball on 3 or 4 pins on a box and add the number of pins reversed (if no pins you can put on ball on a cone)	*In plenum* then by team	20'
Reflex circle	*In plenum* (or two groups	5'
**Lesson 27: Tuesday and Thursday**		
Standard warm‐up + Mexican hat + Rolabola + Pedal boat + Balance mat	*In plenum*	15'
Seated balance (gain) ‐ Sitting on a swissball, feet either on a bottom of the box, then on a medicine ball then on a volleyball/basketball then on another swissball ‐ Bench attached to the wall bars: descents seated on a carpet without touching with your feet (optional: at the same time aim at the stakes with small hoops) ‐ Bench suspended from the rings in the right place: sit on one and the other moves	*In plenum* (depending on the number of participants three alternating groups, 7' per station)	25'
Game: tail catch	*In plenum*	5'
Balance (repetitions) ‐ Bar on the rings: one side of the bench in the place fixed on the bar: up‐down ‐ Bench upside down on one side on a small mat ‐ Bench upside down on a 16 mat ⇨ Cross the benches, make a complete turn, stand on one foot	*In plenum*	15'
**Lesson 28: Friday**		
Warming up + in a circle, pass each other with a volleyball as quickly as possible (in groups if too many participants), always staying in motion + same thing on one foot + passing the ball with the foot (football)	*In plenum*	15'
Game: two teams or three ‐ Bar on the rings: one side of the bench in the place fixed on the bar: up‐down ⇨ At the end of each bench, a box with a mikado on it	By team	10'
Bar on the rings: one side of the bench at the place fixed on the bar: go up‐down (put the rings low enough) ⇨ When you reach the top, get on one foot ⇨ Arrived at the top, on two feet, eyes closed	*In plenum*	15'
Bar on the rings: fixed bench upside down on top ⇨ Go up go down ⇨ Arrived at the top, stand on one foot (to see if possible) If many participants: install a bench upside down on two small mats at each end	*In plenum*	15'
Reflex circle if time	*In plenum*	5'
Exercises: week 11	Organization	Time
**Lesson 27: Tuesday and Friday**		
Standard warm‐up + Mexican hat + Small board that rocks		15'
Bar on the rings: fixed bench upside down on top ⇨ Go up go down ⇨ Arrived at the top, stand on one foot (to see if possible) (if too easy you can put a small mat under the side which is on the ground and it moves even more)	*In plenum*	20'
Three positions: ‐ Bench upside down on one side on a small mat ‐ Bench upside down on a 16 mat ⇨ Cross the benches, make a complete turn, stand on one foot ‐ Reck bar (fixed at the lowest): walk on it, stand on one foot	7 min per shift	20'
Game: day / night		5'
**Lesson 28: Wednesday and Thursday**		
Standard warm‐up		5'
1. Box top on basketballs 2. Bench hang on the rings (right side up): go up to where you feel comfortable then stand on the side and a third person moves the side of the rings 3. Reverse bench on sticks: move back and forth 4. Bench upside down on casters: slalom between poles 5. Empty the carpet carts: one standing then the other moving in all directions	7' per post	40'
In a relaxed way to have fun + one throws a small ball the other catches with a cone (on the ground then on a bench upside down) + badminton (on the ground then on the benches)		15'
Exercises: 12	Organization	Time
**Lesson 29: Monday and Wednesday**		
Standard warm‐up + In pairs, one walks in a straight line on the ground, the other on the side holds a swissball and lightly pushes the one walking to destabilize him. ⇨ Also with eyes closed	*In plenum*	15'
Bar on the rings: fixed bench upside down on top ⇨ Go up go down ⇨ Arrived at the top, stand on one foot (to see if possible) (if too easy you can put a small mat under the side which is on the ground and it moves even more) **Those who are not busy: Mexican hat**	*In plenum*: make two tracks	15'
Tail catcher: to relax		5'
Upside down bench on sticks ‐ In the direction of the bench: two on top and each moves the bench (kind of a little fight) ATTENTION: put yourself between the sticks ‐ Position yourself perpendicular to the bench and another moves the bench (being at the bottom of the bench) ‐ One throws a volleyball and a small ball (always a little changed) and the other on the bench perpendicularly catches.	*In plenum*: make two or three tracks (depending on the number of sticks available) If there is not enough bench, do not hesitate to do the same thing but on a bench that does not move (for those who are not sure).	20'
On the bench: small fight in the form of a game so relax with swissball (each holds a swissball or both hold one) then try to unbalance (you have to try on the ground first if each takes a swissball so that they understand that you have to go gently)	By two + two who secure	5'
**Lesson 30: Tuesday and Friday**		
Standard warm‐up + on bench upside down 1st task: count to 3, repeat faster and faster 2nd task: raise the left hand in place of the 2 3rd task: touch your right knee with your left hand 4th task return to 1st task		15'
Bench on casters: ‐ On two feet: move right – left ‐ On one foot: quietly move forward Carpet cart: ‐ On two feet: move ⇨ Then on one foot **Those who are not busy: Mexican hat**	By alternating group	20'
Game: the circle: one person in the middle: the others try to change places by making a discreet sign and the one in the middle must try to take the place in the circle		10'
Bench upside down on a small mat (on one or both sides) ⇨ Cross the benches, make a complete turn, stand on one foot Upside down bench on two small mats ⇨ To walk on ⇨ Stop in the middle stand on one foot ⇨ Stop in the middle and stand perpendicular ⇨ One on the bench and the other on the ground passes to him (without moving)	By alternating group	20'
Exercises: 13	Organization	Time
**Lesson 31: Monday and Wednesday**		
Standard warm‐up + passage on the benches with counting backwards + Mexican hat + board		20'
Game: catch tail by team (blue against red for example)		
Bench upside down on a small mat (on one or both sides) ⇨ Cross the benches, make a complete turn, stand on one foot Bench upside down on two small mats (one on each side) ⇨ To walk on ⇨ Stop in the middle stand on one foot ⇨ Giant step ⇨ Airplane – small ball ⇨ Moving in chased steps ⇨ Stop in the middle and stand perpendicular ⇨ One on the bench and the other on the ground passes him with a volleyball (without moving)	By alternating group	25'
Little theraband fight on the moving benches		10'
**Lesson 32: Tuesday and Thursday**		
Standard warm‐up + giant steps + throw a volleyball: important to look for lateral movement (not chased) + one throws the ball and the other has his back turned: the first says up and throws the ball and the second turns around and catches the ball + Mexican hat		20'
One closes his eyes and the other turns it on itself (4–5 times) then stops it: the other must: ‐ Walk straight ‐ The moment he stops it, open your eyes and get on one foot		10'
Bench upside down on two small mats (one on each side) or one side: ‐ Not chased ‐ Throw a small ball and the other catches it with an upside‐down cone ‐ Obstacle crossing (holding a hurdle bar)	By alternating group	25'
Jump from the top of a box onto an upside‐down bench or from the mats (but the mat is soft and therefore more difficult) and stabilize on the band		
Game: day/night		
**Lesson 33: Friday**		
Standard warm‐up + on one foot: pick up balls of different sizes and weights then bring them back above your head + Mexican hat		25'
Game: day/night with ball (PLEASE NOTE: this is still a warm‐up) ⇨ Each one a small ball: if you say day, day throw the ball to night and run away and night catches the ball and tries to catch day and vice versa.		10'
On the moving bench: either a small mat underneath on one side, or on both sides: Estafette per team (two teams): Run one end, cross a bench which is on two small mats, slalom between pole, bench box bench, return passage on a large mat and give the relay to the next one.	By alternating group	10'
On the moving bench ‐ Theraband: take both ends of a theraband in your hands and place one foot on the loop then push down ‐ Theraband: hold one side in one hand and the other under the foot then with the remaining foot push to the side ‐ Hold both ends of the theraband in your hands and pass your leg over them		15'
Passing the small hoop on the bench (without it moving) in the form of a race		5'
Exercises: 14	Organization	Time
**Lesson 34: Monday and Wednesday**		
Standard warm‐up + Mexican hat + boards	Groups for Mexican hats and planchettes	15'
One closes his eyes and the other turns it on itself (4–5 times) then stops it: the other must: ‐ Walk straight ‐ The moment he stops it, open your eyes and get on one foot	By 2	10'
Upside down benches on a small mat (on one side) ‐ Walk normal ‐ Make a complete turn in the middle of the bench ‐ Get on one foot	One walking on the bench, one on each side to provide security just in case, one on the mat towards the end of the bench to stabilize if the bench moves too much => ideally groups of four	20'
Estafette with benches upside down on a small carpet on one side. 1–2 m behind the carpet, place a box without the top‐ Cross the bench with a volleyball in your hands, at the end of the bench try to throw the ball into the box. Inside = 1 pt Outside = 0 pt	By team Not a race, what counts is the number of points scored. For example, do two passes per person. Belay as in the previous exercise.	15'
**Lesson 35: Tuesday and Thursday**		
Standard warm‐up + plane + small ball + reflex circle (in a circle, everyone has a stick, say ‘left’ or ‘right’ and everyone moves to the right side to grab the stick that is there)	*In plenum*	15'
Three upside‐down benches in a line with box top between two (continuous line that participants can do without getting off) ‐ Cross normal ‐ Stop in the middle of each bench o Stand on one foot o Make the little ball o Make the plane ‐ Cross counting backwards	One person walking on the bench and one on each side to provide security just in case	20'
Bench upside down on two small mats (one on each side) ‐ Cross normal ‐ Obstacle crossing (holding a hurdle bar) ‐ Moving in chased steps ‐ Moving in a shuffled step, in the middle throw a volleyball into a box (put a box without the top not too far from the bench)	One walking on the bench, one on each side to provide security just in case, one towards one end of the bench to stabilize if the bench moves too much	20'
Small theraband fight on benches in reverse: two people on the bench, they hold the theraband together (the middle with both hands, and the other the two ends), try to destabilize the other by pulling / moving the theraband	People on the sides to provide insurance just in case	10'
