# Supplementary material for: Improved subjective sleep quality in older adults by enhancing the GABAergic system in the sensorimotor cortex
Source: J Physiol. 2026 Jul 5;604(15):6506–46. doi: 10.1113/JP290164 (PMC13423130; doi:10.1113/JP290164)
Supplement: Supplementary file 1 — Peer Review History [file TJP-604-6506-s001.pdf]

The Journal of Physiology

Improved Subjective Sleep Quality in Older Adults by Enhancing the GABAergic System in the Sensorimotor Cortex

Selin Scherrer, Sven Egger, Xinyu Liu, Anna Zoé Wick, Björn Rasch, Lijing Xin, Benedikt Lauber, and Wolfgang Taube
DOI: 10.1113/JP290164

Corresponding author(s): Selin Scherrer (selin.scherrer@unifr.ch)

The following individual(s) involved in review of this submission have agreed to reveal their identity: Emmet McNickle (Referee #1)

Review Timeline:

Submission Date:	24-Sep-2025
Editorial Decision:	08-Apr-2026
Revision Received:	15-May-2026
Editorial Decision:	03-Jun-2026
Revision Received:	03-Jun-2026
Editorial Decision:	08-Jun-2026
Revision Received:	08-Jun-2026
Accepted:	15-Jun-2026

Senior Editor: Richard Carson

Reviewing Editor: Kathy Ruddy

Transaction Report:

(Note: With the exception of the correction of typographical or spelling errors that could be a source of ambiguity, letters and reports are not edited. Depending on transfer agreements, referee reports obtained elsewhere may or may not be included in this compilation. Referee reports are anonymous unless the Referee chooses to sign their reports.)

Re: JP-RP-2025-290164 "**Improved sleep in older adults by enhancing the GABAergic system in the sensorimotor cortex**" by Selin Scherrer, Sven Egger, Xinyu Liu, Anna Zoé Wick, Björn Rasch, Lijing Xin, Benedikt Lauber, and Wolfgang Taube

Dear Dr Scherrer,

Thank you for submitting your manuscript to The Journal of Physiology. It has been assessed by a Reviewing Editor and by 2 expert referees and we are pleased to tell you that it is potentially acceptable for publication following satisfactory major revision.

Please advise your co-authors of this decision as soon as possible.

The referee reports are copied at the end of this email.

Please address all the points raised and incorporate all requested revisions or explain in your Response to Referees why a change has not been made. We hope you will find the comments helpful and that you will be able to return your revised manuscript within 2 months. If your article is NOT for a Special Issue, you may have 9 months to revise. If you require an extension, please contact journal staff: jp@physoc.org. Please note that this letter does not constitute a guarantee for acceptance of your revised manuscript.

Your revised manuscript should be submitted online using the link in your Author Tasks: Link Not Available. This link is accessible via your account as Corresponding Author; it is not available to your co-authors. If this presents a problem, please contact journal staff (jp@physoc.org). Image files from the previous version are retained on the system. Please ensure you replace or remove any files that are being revised.

If you do not wish to submit a revised version of your manuscript, you must inform our journal staff (jp@physoc.org) or reply to this email to request withdrawal. Please note that a manuscript must be formally withdrawn from the peer review process at one journal before it may be submitted to another journal.

TRANSPARENT PEER REVIEW POLICY: To improve the transparency of its peer review process, The Journal of Physiology publishes online as supporting information the peer review history of all articles accepted for publication. Readers will have access to decision letters, including Editors' comments and referee reports, for each version of the manuscript, as well as any author responses to peer review comments. Referees can decide whether or not they wish to be named on the peer review history document.

ABSTRACT FIGURES: Authors are expected to use The Journal's premium BioRender account to create/redraw their Abstract Figures. Information on how to access this account is here: https://physoc.onlinelibrary.wiley.com/journal/14697793/biorender-access.

This will enable Authors to create and download high-resolution figures. If authors have used the free BioRender service, they can use the instructions provided in the link above to download a high-resolution version suitable for publication.

The link provided should only be used for the purposes of this submission. Authors will be charged for figures created on this account if they are not related to this manuscript submission.

LANGUAGE EDITING AND SUPPORT FOR PUBLICATION: If you would like help with English language editing, or other article preparation support, Wiley Editing Services offers expert help, including English Language Editing, as well as translation, manuscript formatting, and figure formatting at www.wileyauthors.com/eeo/preparation. You can also find resources for Preparing Your Article for general guidance about writing and preparing your manuscript at www.wileyauthors.com/eeo/prepresources.

RIGOUR AND REPRODUCIBILITY: We recommend authors consult The Journal's Rigour and Reproducibility requirements to ensure that their manuscript is suitable for publication: https://physoc.onlinelibrary.wiley.com/pb-assets/hub-assets/physoc/documents/TJP-Rigour-and-Reproducibility-Requirements.pdf

REVISION CHECKLIST:

Check that your Methods section conforms to journal policy: https://jp.msubmit.net/cgi-bin/main.plex?form_type=display_requirements#methods.

Check that data presented conforms to the statistics policy: https://jp.msubmit.net/cgi-bin/main.plex?form_type=display_requirements#statistics.

Upload a full Response to Referees file. To create your 'Response to Referees': copy all the reports, including any comments from the Senior and Reviewing Editors, into a Microsoft Word, or similar, file and respond to each point, using font or background colour to distinguish comments and responses and upload as the required file type.

Please upload two versions of your manuscript text: one with all relevant changes highlighted and one clean version with no changes tracked. The manuscript file should include all tables and figure legends, but each figure/graph should be uploaded as separate, high-resolution files.

You may also upload:

- 'Potential Cover Art' for consideration as the issue's cover image
- Appropriate Supporting Information (Video, audio or data set: see https://jp.msubmit.net/cgi-bin/main.plex?form_type=display_requirements#supp).

We look forward to receiving your revised submission.

If you have any queries, please reply to this email and we will be pleased to advise.

Yours sincerely,

Richard Carson
Senior Editor
The Journal of Physiology

REQUIRED ITEMS

- Author photo and profile. First or joint first authors are asked to provide a short biography (no more than 100 words for one author or 150 words in total for joint first authors) and a portrait photograph. These should be uploaded and clearly labelled together in a Word document with the revised version of the manuscript. See Information for Authors for further details.

- You must start the Methods section with a paragraph headed Ethical Approval. If experiments were conducted on humans, confirmation that informed consent was obtained, preferably in writing needs to be stated. For further information, see: https://physoc.onlinelibrary.wiley.com/hub/human-experiments.

- Please upload separate high-quality figure files via the submission form.

- Papers must comply with the Statistics Policy: https://jp.msubmit.net/cgi-bin/main.plex?form_type=display_requirements#statistics.

In summary:
* The most appropriate summary statistic (e.g. mean or median and standard deviation) must be used. Standard Error of the Mean (SEM) alone is not permitted.

- Please include an Abstract Figure file and an Abstract Figure legend. An appropriate figure legend, which should not exceed 150 words in length, should be included in the main manuscript file. The Abstract Figure is a piece of artwork designed to give readers an immediate understanding of the research and should summarise the main conclusions. If possible, the image should be easily 'readable' from left to right or top to bottom. It should show the physiological relevance of the manuscript so readers can assess the importance and content of its findings. Abstract Figures should not merely recapitulate other figures in the manuscript. Please try to keep the diagram as simple as possible and without superfluous information that may distract from the main conclusion(s). Abstract Figures must be provided by authors no later than the revised manuscript stage and should be uploaded as a separate file during online submission labelled as File Type 'Abstract Figure'. Please also ensure that you include the figure legend in the main article file. All Abstract Figures should be created using BioRender. Authors should use The Journal's premium BioRender account to export high-resolution images. Details on how to use and access the premium account are included as part of this email.

EDITOR COMMENTS

Reviewing Editor:

Please provide clarification to the reviewers on the points raised regarding methodological details and the validity of the statistical approach in a revised manuscript.

Senior Editor:

It is recognise that this is an ambitious study motivated by clearly stated hypotheses.

Before further consideration can be given to the submission however, the editors would wish to be assured that the stated outcomes remain apparent when the statistical analysis procedures are revised in line with the recommendations that have been made.

In addition to the specific points raised by the reviewers, it seems to me notable that steps do not appear to have been taken to verify the distributional properties (i.e., with respect to normality) of the various dependent measures. Although, with respect to certain measures, the authors note "square root transformed before statistical analyses to ensure normal distribution of residuals", the distribution of the residuals (or of other measures) does not appear to have been reported.

These matters are of particular importance given the use of parametric tests, such as Pearson's correlation.

In this vein also, please note that ratio measures - such as those used to characterise levels of SICI do not typically follow a normal distribution (see PMID: 39234878).

With respect to the analysis of MEPs, it is noted that "MEPs with a bEMG more than three times the median absolute deviation (MAD) from the median bEMG were excluded from data analysis (Leys et al., 2013). This does not provide an adequate or appropriate means of dealing with instances in which the post-synaptic excitability of the spinal motoneuron pool (as reflected in the background EMG) may have had an instrumental bearing on the magnitude of the MEPs. Specifically, the method employed does not deal with circumstances in which the background EMG is elevated, but rather only with instances in which there were large deviations from the typical level of background EMG exhibited by a given participant. At the very least, it should be demonstrated that the levels of background EMG were sufficiently small that the influence of this factor on MEP amplitude might reasonably be deemed to be small, i.e., relative to other factors.

In this regard, the appearance of an exclusion method in a prior publication (e.g., Leys et al., 2013) provides no assurance in respect of the adequacy of that method.

REFEREE COMMENTS

Referee #1:

Overall evaluation and impact

This is a high-quality and original study. It combines a well-controlled behavioural intervention (balance training) with multimodal neuroimaging and neurophysiology in an older adult cohort to probe the GABAergic inhibitory system and its role in sleep regulation. The work identifies the sensorimotor cortex as potentially modifiable for sleep health in older adults, an area rarely targeted by sleep research and of clear translational relevance for non-pharmacological treatment of age-related sleep disturbance. The topic fits well with the Journal of Physiology's scope.

Strengths

The authors innovatively integrated behavioural, neurochemical (7 T MRS), neurophysiological (TMS), and fMRI measures. There was a clear focus on an important and under-addressed physiological mechanism (age-related decline in GABAergic inhibition). The manuscript includes a rigorous description of intervention, imaging, and TMS methodology, with open sharing of data and analysis code on OFS which is very encouraging. The results are of practical clinical relevance as a potential safe alternative to pharmacological GABA agonists for older adults.

Limitations and requested clarifications

- Unexpected GABA/sleep association. The finding of greater GABA increases linked to smaller PSQI improvement runs opposite to what might have been expected prior to the study. The "capacity, then functional use" explanation is described in line with the same group's recent review paper on two-layered GABA function in sleep, so in a sense is not implausible but does appear post-hoc. Please make this explicit in the abstract (and in the introduction and discussion). In particular, in the abstract states that 'regression analyses indicated that changes in GABA levels and in functional connectivity significantly predicted improvements in subjective sleep quality'. This should be reworded to ensure the counterintuitive direction of the correlation between GABA and sleep is clear and does not mislead the reader.

- Pre-registration and analytical transparency. While data and code are openly shared, I was slightly disappointed to see that hypotheses were not preregistered. Please state this plainly and indicate which analyses were planned vs exploratory.

- The group by time interactions for SICI were not significant; n for SICI during sleep was small. I would suggest therefore that these results are presented as suggestive/exploratory. The authors do acknowledge the limited sample size and possible under-powering due to a few participants who they couldn't get TMS measures from while asleep.

- Methods.

-Clarify participant recruitment/compensation.

-State whether paired-pulse TMS acquisition and analysis was blinded in relation to whether participants were in the control or the balance training group, or note as a limitation.

-Discuss nap vs night-time SICI generalisability and the rationale for measuring during N1 as opposed to another stage.

-Briefly explain the 7 mm coil drift tolerance and whether many epochs were excluded because of movement.

-There was a strength-training control condition..? I can see the supplementary results for it but not the supplementary method section - would like to know more (separate group? N?) as it is an interesting control.

-Were any measures taken of activities in the main 'habitual activity' control group?

- Discussion. Line 580: specifies how "balance training improves subjective sleep quality by at least partly reversing age-related changes in the sensorimotor cortex". I think we can say that it improves subjective sleep quality, but in the absence of longitudinal components it goes beyond the data to say that it "reverses age-related changes".

Referee #2:

In the current study, the authors compared the effect of a 3-month balance training on sleep quality, with objective and subjective markers. They found a positive effect of this intervention on balance performance and subjective sleep quality. These changes were associated with modulations of some inhibitory mechanisms, across all participants.

To note that this is an ambitious study with strong hypotheses and multiple state-of-the-art techniques to probe inhibitory markers within the sensorimotor and motor system.

While the intervention showed evidence of beneficial effects on balance, subjective sleep quality and specific brain markers, the statistical design of simple and multiple regressions and their interpretations should be revised to fully support the current data.

Please find below my general comments and minor remarks

General remarks:

The main concern relates to the statistical analyses and their interpretation.

- First, the authors found an interaction between group and time for balance performance (sway area). What was the difference between groups at post-test?

- The simple and multiple regressions should be revised: Including both control and experimental participants in a regression analysis aimed at explaining training-related improvements may introduce substantial confounding, as performance changes are inherently structured by group assignment. A more appropriate approach would involve restricting the analysis to the experimental group or explicitly modelling group-by-parameter interactions.

- l.411: "Furthermore, we analysed the Pearson correlation coefficient between changes in PSQI scores and changes in SICI during sleep initiation".

What is « sleep initiation »? Weren't the participants asleep?

- l.414: "To evaluate whether the increase in GABA levels was associated with baseline GABA levels, we calculated the Pearson correlation coefficient between baseline GABA levels and the change in GABA levels".

What was the purpose of such analysis? How does this relate to the main hypothesis? (age, sleep, balance training)?

- Could the authors provide more details about the regression analysis, such as Durbin-Watson test (autocorrelation in the residuals), and VIF scores (absence of multicollinearity)?

Minor remarks

- To fit the results of the current study, I would suggest changing the title of the paper, as "Improved Subjective sleep quality in older adults by enhancing the GABAergic system in the sensorimotor cortex".

- The control group did not perform any other task and was not supervised. This might be mentioned it in the limitation section?

- Performing TMS during sleep poses a real challenge. Could the authors briefly explain how they ensured that the participants were still asleep throughout the TMS session?

- l.539-540: "while the control group showed a non-significant decrease by 0.24 (SEM = 0.26) after three months of habitual living". If the difference pre-post is not significant, the term "decrease" is not appropriate. I would suggest "while the control group did not show any change after three months of habitual living (delta = 0.24, SEM = 0.26 ; (t(15) = -0.91, pbonferroni = 541 .752, d = -0.23 [-0.72; 0.27])."

- l.618-619: "These high baseline levels may partly explain the absence of significant changes following the balance training. ». What are the baseline levels?

END OF COMMENTS

EDITOR COMMENTS

Reviewing Editor:

Please provide clarification to the reviewers on the points raised regarding methodological details and the validity of the statistical approach in a revised manuscript.

We thank the Reviewing Editor for the guidance. In the revised manuscript, we have clarified the methodological details and expanded the justification and reporting of the statistical analyses, as requested. These changes are detailed in our responses to the reviewers' comments below and are highlighted in the revised manuscript.

Furthermore, we have consulted the statistics and journal policy and have made some changes, including deletion of numbers from the abstract, reporting of standard deviation instead of standard error of the mean, reporting p-values to the third significant figure, adding an Ethical approval subsection at the start of the methods section, and we removed claims as to the primacy of our research.

Senior Editor:

It is recognise that this is an ambitious study motivated by clearly stated hypotheses.

Before further consideration can be given to the submission however, the editors would wish to be assured that the stated outcomes remain apparent when the statistical analysis procedures are revised in line with the recommendations that have been made.

We thank the Senior Editor for the constructive assessment of our study. In response to the concerns raised, we have revised the statistical analysis procedures in line with the recommendations provided. The principal outcomes and thus the conclusions of the study remain unchanged following these revisions. Full details of the revised analyses and their impact on the results are provided in response to the specific comments.

In addition to the specific points raised by the reviewers, it seems to me notable that steps do not appear to have been taken to verify the distributional properties (i.e., with respect to normality) of the various dependent measures. Although, with respect to certain measures, the authors note "square root transformed before statistical analyses to ensure normal distribution of residuals", the distribution of the residuals (or of other measures) does not appear to have been reported.

We thank you for raising this important point. In the revised manuscript, we have clarified the procedures used to assess distributional assumptions. Specifically, normality was evaluated at the level of model residuals rather than raw outcome measures, using visual inspection (Q-Q-plots). Furthermore, Levene's test assessing similar variances across groups and Bartlett's test assessing sphericity were performed. For measures that deviated considerably from normality, transformations were applied, and the resulting residual distributions were confirmed to be acceptable. These procedures and their outcomes are now explicitly described in the statistical analysis section.

The normality of the residuals for the balance parameter is borderline, so we calculated the repeated measures ANOVA additionally without the outlier and with a linear mixed effects model

for which the residuals were normally distributed. Both sensitivity analyses came to the same statistical conclusion. For PSQI and SICI while balancing, exclusion of outliers did not lead to improved distributional properties, and we transformed the data to meet the assumptions. For GABA, functional connectivity, and SICI during sleep the assumptions were sufficiently met.

Our changes in the methods section read as follows:

[P. 16, L. 423] "Comparisons of changes over time between groups were assessed by two-way repeated-measures analysis of variance (ANOVA). Assumptions were assessed with normal q-q plots of model residuals, Levene's test to test for similar variances across groups, and Bartlett's test to assess sphericity."

[P. 16, L. 440] "Model assumptions were evaluated using the Durbin-Watson test for residual autocorrelation and variance inflation factors (VIF) to assess multicollinearity among predictors (VIF < 5). "

These matters are of particular importance given the use of parametric tests, such as Pearson's correlation.

Furthermore, we assessed normal distribution of change values of the parameters included in correlation analysis with q-q-plots and assessed whether the data included any outliers more than three standard deviations away of the mean. If so, we calculated the correlation with and without outlier(s) and compared the results. This was the case for the correlation between SICI while balancing and balance performance and we came to the same statistical conclusion with and without inclusion of outliers. So, we reported the results with all data included.

The corresponding addition to the methods section:

[P. 17, L. 451] "Assumptions for correlation analyses were verified with q-q-plots for normal distribution of the difference scores of each included parameter and with screening for outliers more than three SD away from the mean. Where outliers were detected, we assessed correlation with and without outliers to verify the result."

In this vein also, please note that ratio measures - such as those used to characterise levels of SICI do not typically follow a normal distribution (see PMID: 39234878).

Thank you for sharing this interesting approach. Indeed, the residuals of the SICI while balancing values were not normally distributed. However, we could not reach normality with the PCA approach suggested in the reference. Furthermore, as we had some negative values in our data, we could not do the established transformations like square root or logarithmic transformation. So, we applied Yeo-Johnson transformation, which led to normally distributed residuals.

With respect to the analysis of MEPs, it is noted that "MEPs with a bEMG more than three times the median absolute deviation (MAD) from the median bEMG were excluded from data analysis (Leys et al., 2013). This does not provide an adequate or appropriate means of dealing with instances in which the post-synaptic excitability of the spinal motoneuron pool (as reflected in the background EMG) may have had an instrumental bearing on the magnitude of the MEPs. Specifically, the method employed does not deal with circumstances in which the background EMG is elevated, but rather only with instances in which there were large deviations from the typical level of background EMG exhibited by a given participant. At the very least, it should be demonstrated that the levels of background EMG were sufficiently small that the influence of this factor on MEP amplitude might

reasonably be deemed to be small, i.e., relative to other factors.
In this regard, the appearance of an exclusion method in a prior publication (e.g., Leys et al., 2013) provides no assurance in respect of the adequacy of that method.

We thank the editor for this important comment on our SICI analysis. As we measured SICI during active balancing, we wanted to make sure that we analysed stimulations that occurred at similar muscle activation. We deleted the following number of MEPs because the background activity was not within 3x MAD from the median of all bEMGs of this participant and timepoint (see table). With a total number of 2880 stimulations (36 participants x 2 timepoints x 40 stimulations), we deleted only 5.7% of all stimulations.

	**Control group**	
Timepoint	Single-pulse MEP deleted	Paired-pulse MEP deleted
Pre	29	31
Post	20	11
	**Balance group**	
Timepoint	Single-pulse MEP deleted	Paired-pulse MEP deleted
Pre	14	20
Post	18	21

Comparing background activity for paired-pulse stimulations with that of single-pulse stimulations per participant and per timepoint after deletion of the outliers, all t-tests were non-significant.

In the balance group, mean bEMG did not differ between PRE and POST (PRE: M = 0.056, SD = 0.039; POST: M = 0.056, SD = 0.036; p-value t-test: .980), nor did the amplitude of the single-pulse stimulation (PRE: M = 1.794, SD = 1.071; POST: M = 1.808, SD = 0.835; p-value t-test: .932). In the control group, we did not see significant differences in bEMG between timepoints (PRE: M = 0.042, SD = 0.035; POST: M = 0.070, SD = 0.0604; p-value t-test: .114), nor in amplitude of the single-pulse stimulation (PRE: M = 1.636, SD = 0.657; POST: 1.902, SD = 0.915; p-value t-test: 0.325).

REFEREE COMMENTS

Referee #1:

Overall evaluation and impact

This is a high-quality and original study. It combines a well-controlled behavioural intervention (balance training) with multimodal neuroimaging and neurophysiology in an older adult cohort to probe the GABAergic inhibitory system and its role in sleep regulation. The work identifies the sensorimotor cortex as potentially modifiable for sleep health in older adults, an area rarely targeted by sleep research and of clear translational relevance for non-pharmacological treatment of age-related sleep disturbance. The topic fits well with the Journal of Physiology's scope.

Strengths

The authors innovatively integrated behavioural, neurochemical (7 T MRS), neurophysiological (TMS), and fMRI measures. There was a clear focus on an important and under-addressed physiological mechanism (age-related decline in GABAergic inhibition). The manuscript includes a rigorous description of intervention, imaging, and TMS methodology, with open sharing of data and analysis code on OFS which is very encouraging. The results are of practical clinical relevance as a potential safe alternative to pharmacological GABA agonists for older adults.

Limitations and requested clarifications

- Unexpected GABA/sleep association. The finding of greater GABA increases linked to smaller PSQI improvement runs opposite to what might have been expected prior to the study. The "capacity, then functional use" explanation is described in line with the same group's recent review paper on two-layered GABA function in sleep, so in a sense is not implausible but does appear post-hoc. Please make this explicit in the abstract (and in the introduction and discussion). In particular, in the abstract states that 'regression analyses indicated that changes in GABA levels and in functional connectivity significantly predicted improvements in subjective sleep quality'. This should be reworded to ensure the counterintuitive direction of the correlation between GABA and sleep is clear and does not mislead the reader.

Thank you for this important comment. We agree that the association between training-related increases in sensorimotor GABA and improvements in subjective sleep quality is counterintuitive and that the abstract should make the direction of this relationship explicit to avoid potential misinterpretation. We have therefore revised the abstract to state clearly that greater increases in functional connectivity were associated with larger improvements in subjective sleep quality, whereas greater increases in GABA levels were associated with smaller improvements. In addition, we have revised the introduction and discussion to make explicit that our “capacity vs function use” interpretation is post-hoc and interpretative, and we now frame it accordingly.

The negative relationship between ΔPSQI and ΔGABA is now explicitly stated in the abstract (the page and line numbers refer to the document with tracked changes):

[P. 3, L. 30] "Regression analyses indicated that greater increases in functional connectivity were associated with larger improvements in subjective sleep quality, whereas greater increases in GABA levels were associated with smaller improvements."

Introduction:

[P. 4, L. 71] "However, it remains unclear how intervention-induced increases in inhibitory capacity (GABA levels) and functional use of inhibition (SICI) relate to changes in subjective sleep quality in older adults."

Discussion:

[P. 28, L. 706] "Although these adaptations seem contradictory at first, one possible explanation is consistent with the two-layer model of inhibition introduced by Taube and Lauber (2024). Based on this model, the increase in GABA levels may indeed enhance the capacity for inhibition, whereas improvements in sleep may depend more strongly on the functional modulation of inhibition."

[P. 31, L. 823] "This study shows improved subjective sleep quality after three months of balance training in older adults, alongside changes in inhibitory markers and sensorimotor network coupling."

- Pre-registration and analytical transparency. While data and code are openly shared, I was slightly disappointed to see that hypotheses were not preregistered. Please state this plainly and indicate which analyses were planned vs exploratory.

We thank the reviewer for this important comment. The study was not preregistered, and this has now been stated explicitly in the Ethical approval paragraph of the methods section. To improve analytical transparency, we have clarified explicitly in the Statistical Analysis section which hypotheses were exploratory.

We additionally provide documentation that the primary hypotheses were formulated before data analysis, as they were described in our funding application (if wished so, we can send the whole document):

after balance and strength learning (post-test)

- H6: MRS-based GABA level will be enhanced in the motor learning groups (young and old) but not in the control groups
- H7: TMS-based GABA modulation will be enhanced in the motor learning groups (young and old) but not in the control groups during motor execution
- H8: Training-related improvements in modulatory capacity of intracortical inhibition are transferred to sleep resulting in better GABA modulation during sleep in the motor learning groups (young and old) but not in the control groups
- H9: Subjects of the balance learning groups (young and old) will sleep better after the training intervention than subjects of the control and strength training groups indicated by improved scores in the sleep questionnaires and the EEG-based parameters. It is hypothesized that especially 'sleep onset latency' is reduced due to better and more efficient modulation of GABA. The improved modulatory capacity of the GABAergic system is also assumed to reduce 'beta activity' and to enhance 'slow-wave sleep'.
- H10: In general, the training-induced changes both at the behavioral and the neurophysiological/-chemical level are thought to be more pronounced in the elderly, approaching the baseline level of the young subjects.
- H11: Subjects who participate in the balance training and demonstrate greater changes in the level and modulation of GABA will show more pronounced changes sleeping behavior

Missing pre-registration is now stated in the methods section:

[P. 7, L. 149] "The study was completed in accordance with the Declaration of Helsinki, except for pre-registration in a publicly accessible database."

We added the following clarification in the methods section:

[P. 17, L. 458] "Exploratory analyses

In addition to the primary hypotheses and associated analyses that were defined a priori based on previous literature and our study objectives, we performed the following additional analysis. As the effect of ΔGABA levels on ΔPSQI was negative, we wanted to test whether our data support an interpretation in the context of the model suggested by Lauber & Taube (2024). Therefore, we calculated the Pearson correlation coefficient between baseline GABA levels and the change in GABA levels."

- The group by time interactions for SICI were not significant; n for SICI during sleep was small. I would suggest therefore that these results are presented as suggestive/exploratory. The authors do

acknowledge the limited sample size and possible under-powering due to a few participants who they couldn't get TMS measures from while asleep.

We thank the reviewer for this thoughtful comment. We agree that the limited sample size for SICI during sleep reduces statistical power and warrants caution in interpretation. This is now more explicitly acknowledged in the discussion section. However, we would like to clarify that the group x time analysis and the Pearson correlation including SICI during sleep was planned a priori and directly addressed a central study hypothesis. Therefore, we have kept the analysis as hypothesis-driven rather than exploratory.

The change in the discussion section reads as follows:

[P. 29, L. 735] " Unfortunately, only 26 participants were able to sleep during the afternoon nap and keep head movement within acceptable limits for TMS during both PRE and POST measurements. Thus, the amount of data points is limited for SICI during sleep. Although this analysis was planned a priori and directly addressed our primary hypothesis, the reduced number of participants for whom SICI could be reliably obtained during sleep limits statistical power. Consequently, these findings should be interpreted cautiously and considered suggestive rather than conclusive, pending replication in larger samples."

- Methods.

-Clarify participant recruitment/compensation.

We thank the reviewer for this suggestion. Participants were recruited through newspaper advertisements and received a compensation of CHF 250.- in accordance with the guidelines of the ethics commission. The methods section has been updated to clarify participant recruitment and compensation:

[P. 7, L. 161] "Participants were recruited through newspaper advertisements, followed by an information session to provide participants with more detailed information about the study procedures, screen for exclusion criteria, and to confirm availability for the required time commitment. Participants received appropriate compensation in accordance with the guidelines of the ethics commission."

-State whether paired-pulse TMS acquisition and analysis was blinded in relation to whether participants were in the control or the balance training group, or note as a limitation.

We thank the reviewer for this important point. Paired-pulse TMS acquisition and subsequent analysis were not explicitly blinded to group allocation. However, the outcomes were objective measures using predefined stimulation parameters and therefore we can assume that the absence of blinding is unlikely to have influenced the results. This has now been mentioned as a limitation in the revised discussion:

[P. 32, L. 839] "(4) The paired-pulse TMS acquisition and analysis were not explicitly blinded to group allocation. However, as the outcomes were objective neurophysiological measures obtained using predefined stimulation parameters, the absence of blinding is unlikely to have influenced the results."

-Discuss nap vs night-time SICI generalisability and the rationale for measuring during N1 as opposed to another stage.

Thank you for this important suggestion. We have now expanded the discussion to address the generalisability of SICI assessed during an afternoon nap to nocturnal sleep, including potential influences of circadian phase, sleep pressure, and arousal threshold.

In addition, we have added further detail to the methods section to clarify the rationale for focusing on sleep onset rather than later sleep stages. Specifically, we now explain that analysing SICI during light sleep (N1) allowed determination of motor threshold in wakefulness with subsequent automated stimulation at pre-calculated intensities and minimisation of disturbance during the nap opportunity.

Methods:

[P. 13, L. 335] "SICI during sleep was assessed during an afternoon nap, which allowed controlled, feasible acquisition during the wake-to-sleep transition. "

[P. 13, L. 350] "N1 sleep is the light sleep phase between being awake and in stable sleep and the MT in this stage is not yet significantly higher compared to lying awake (Avesani et al., 2008). Stimulations during N1 were exported for SICI calculation. SICI assessment during N1 enabled us to determine the MT while the participant was lying awake and to then deliver automated stimulations at pre-calculated intensities and at a regular rhythm, thereby minimizing disturbance during the sleep opportunity. Thus, SICI measurement during N1 sleep allowed us to measure functionally relevant modulation of inhibition during sleep, without having to interrupt the rhythmic stimulations to reassess the motor threshold throughout the afternoon nap."

Discussion:

[P. 28, L. 721] "In this study, SICI was assessed during an afternoon nap to capture intracortical inhibition around sleep onset. Although sleep onset occurs under different conditions during daytime naps versus nocturnal sleep, given a more sleep favourable circadian phase, higher homeostatic pressure, and potentially a lower arousal threshold in the evening, available evidence indicates that SICI is relatively stable across time of day and levels of sleepiness under non-sleep-deprived conditions (Doeltgen & Ridding, 2010; De Gennaro et al., 2007; Manganotti et al., 2001). Moreover, studies assessing SICI across sleep stages report broadly comparable stage-dependent modulation across nocturnal sleep and daytime sleep, with stronger inhibition observed during deeper NREM sleep relative to lighter sleep stages and wake (Avesani et al., 2008; Salih et al., 2005). Taken together, these findings support the use of nap-based sleep onset SICI as a reasonable proxy for inhibitory mechanisms relevant to nocturnal sleep onset."

-Briefly explain the 7 mm coil drift tolerance and whether many epochs were excluded because of movement.

We thank for the comments about the SICI measurement during the afternoon nap. We added more information about the coil drift tolerance in the methods section and added the information about how many participants were excluded because of which reason and how many stimulations were included.

Methods:

[P. 14, L. 365] "Data were excluded if the stimulation point deviated more than 7 mm from the determined hotspot, representing a conservative cutoff relative to prior evidence showing that

conditioning pulses within approximately 8 mm of the hotspot contribute to SICI (Nieminen et al., 2019)."

[P. 14, L. 382] "Regarding SICI during sleep, six participants did not fall asleep at both time points, two participants were excluded from analysis because they moved their head more than 7 mm away from the hotspot before falling asleep, and two participants had to be excluded because of clearly negative SICI values (-25% or lower). We were able to assess SICI during sleep in 11 participants in the balance group and in 15 participants in the control group. Over the 26 included participants, we analysed 2664 stimulations (48 ± 37 stimulations per participant)."

-There was a strength-training control condition..? I can see the supplementary results for it but not the supplementary method section - would like to know more (separate group? N?) as it is an interesting control.

We thank the reviewer for noting this and for highlighting interest in the additional strength training control condition. A supplementary methods section describing this control condition was included; however, in the revised version we have expanded this section and reorganised the presentation for clarity. Specifically, we have added additional methodological details (including sample size and sample characteristics) and moved the explanatory text above the supplementary methods table. The strength training control condition is now fully described in the supplementary methods following the supplementary results.

Additional text in supplementary material:

[P. 3, L. 23] "Participants

Twenty volunteers aged 65 to 79 years were recruited through newspaper advertisements and an information session after first mention of interest. All participants were free of any known neurological and orthopaedic diseases or injuries and contraindications to MRI and TMS measurements. Participants received appropriate compensation in accordance with the guidelines of the ethics commission. Two participants dropped out before POST tests due to detected diseases during the study intervention. Consequently, 18 participants (7 females, 70.11 ± 4.00 years old) were included in the analysis."

-Were any measures taken of activities in the main 'habitual activity' control group?

Thank you for this important question. Activities in the habitual-activity control group were assessed via self-reported physical activity measures collected at both PRE and POST. Specifically, participants reported their weekly exercise frequency, weekly exercise duration, and type(s) of exercise. We have now added this information explicitly to the methods section (Intervention period, Statistical analysis) and reported the PRE-POST comparisons in the results, showing that the control group did not show evidence of changes in exercise frequency, duration, or activity type over the intervention period. We also updated the Limitations section to clarify that, while the control group was not supervised, these self-reported measures support the interpretation that they maintained their habitual exercise behaviour during the study period.

Methods:

[P. 9, L. 206] "To monitor potential baseline differences in habitual physical activity between the groups or changes in the control group throughout the study, participants reported exercise frequency (sessions per week), weekly exercise duration, and activity type(s) at PRE and POST."

[P. 16, L. 421] "We compared weekly exercise frequency and duration at baseline between balance and control group with unpaired t-tests and the changes from PRE to POST in the control group with paired t-tests."

Results:

[P. 17, L. 467] "Self-reported exercise frequency ($t(38) = 0.38$, $p = .703$, $d = 0.12$ [-0.50; 0.74]) and weekly exercise duration ($t(38) = -0.36$, $p = .719$, $d = -0.11$ [-0.73; 0.51]) did not differ between groups at baseline. Furthermore, the control group showed no change in self-reported exercise frequency from PRE to POST ($t(17) = 0.00$, $p = .999$, $d < 0.01$ [-0.46; 0.46]) and no change in weekly exercise duration ($t(17) = -0.62$, $p = .542$, $d = -0.15$ [-0.61; 0.32]). Reported types of sports also remained unchanged over time. These self-reported measures suggest that the control group maintained their habitual exercise behaviour over the intervention period, supporting comparison of the balance intervention against a stable habitual-lifestyle condition."

Discussion:

[P. 32, L. 842] "(5) Furthermore, the control group was passive and not supervised during the intervention period. However, self-reported exercise frequency, duration, and activity type remained stable from PRE to POST. Additionally, a separate strength-training comparison group did not show similar changes, suggesting that the observed effects are unlikely to be attributed to nonspecific engagement alone."

- Discussion. Line 580: specifies how "balance training improves subjective sleep quality by at least partly reversing age-related changes in the sensorimotor cortex". I think we can say that it improves subjective sleep quality, but in the absence of longitudinal components it goes beyond the data to say that it "reverses age-related changes".

We thank the reviewer for this important comment. We agree that, in the absence of a longitudinal aging design, our data do not permit a direct conclusion that balance training "reverses" age-related changes. Our intention was to interpret the observed changes considering existing literature. To avoid overinterpretation, we have revised the discussion to remove the reversal of aging effects. The text now states that balance training improves subjective sleep quality and is associated with changes in sensorimotor inhibition in opposite direction to alterations commonly seen in aging. We believe this wording more accurately reflects the strength and scope of the present data.

[P. 26, L. 646] "The results show that three months of balance training improves subjective sleep quality and is associated with changes in sensorimotor inhibition in opposite direction to alterations commonly seen in aging."

Referee #2:

In the current study, the authors compared the effect of a 3-month balance training on sleep quality, with objective and subjective markers. They found a positive effect of this intervention on balance

performance and subjective sleep quality. These changes were associated with modulations of some inhibitory mechanisms, across all participants.

To note that this is an ambitious study with strong hypotheses and multiple state-of-the-art techniques to probe inhibitory markers within the sensorimotor and motor system.

While the intervention showed evidence of beneficial effects on balance, subjective sleep quality and specific brain markers, the statistical design of simple and multiple regressions and their interpretations should be revised to fully support the current data.

We thank the reviewer for the careful and constructive assessment of our work. In response to the concerns regarding the statistical design and interpretation of regression analyses, we have revised the corresponding sections of the manuscript to improve analytical clarity and transparency, as detailed in our responses to the specific comments below.

Please find below my general comments and minor remarks

General remarks:

The main concern relates to the statistical analyses and their interpretation.

- First, the authors found an interaction between group and time for balance performance (sway area). What was the difference between groups at post-test?

We thank you for this comment. We now explicitly report the between-group comparison at post-test. Although the balance group showed a numerically lower sway area than the control group, this difference did not reach statistical significance. The results section has been revised accordingly, and the training effect is interpreted primarily on the significant group x time interaction.

Results (the page and line numbers refer to the document with tracked changes):

[P. 18, L. 484] "At POST, mean sway area was 35.18 cm^2 lower in the balance group (86.78 cm^2) than in the control group (121.96 cm^2); however, this between-group difference did not reach statistical significance. Together, these results indicate that balance performance improved over time in the balance training group relative to the control group."

- The simple and multiple regressions should be revised: Including both control and experimental participants in a regression analysis aimed at explaining training-related improvements may introduce substantial confounding, as performance changes are inherently structured by group assignment. A more appropriate approach would involve restricting the analysis to the experimental group or explicitly modelling group-by-parameter interactions.

Thank you for this comment. We performed the analyses with all participants because in previous longitudinal studies of our group (e.g. Ruffieux et al, 2017; Kuhn et al, 2024) and other groups (e.g. Zuppichini et al, 2024) comparing elderly participants over several weeks and months, aging per se can influence the neurophysiological parameters (e.g. decrease GABA and SICI). Thus, interventions (medication, physical training, etc.) often rather decelerate age-related decline than improve the

parameter at hand. Adding all subjects to the regression and correlation analyses has the advantage that it provides information not only about the effect of balance training and its capacity to decelerate or reverse the age-related decline but also if aging (as it can be seen in the control group) changes the variables we were interested in. With this approach, we assess training-related and age-related changes at the same time and can see if these changes in neurophysiological parameters are associated with the behavioural parameters of interest, e.g. subjective sleep quality.

In the figures of the simple correlations, we coloured the individual datapoints according to their group affiliation to show more clearly that we performed the correlation analysis with both groups (control and intervention). We also added group-coloured scatterplots showing the correlations of each predictor of the multiple regression model with the outcome variable when holding the other predictors steady.

Kuhn, Y.-Y., Egger, S., Bugnon, M., Lehmann, N., Taubert, M., Taube, W., 2024. Age-related decline in GABAergic intracortical inhibition can be counteracted by long-term learning of balance skills. J Physiol 602, 3737–3753. https://doi.org/10.1113/JP285706

Ruffieux, J., Mouthon, A., Keller, M., Walchli, M., Taube, W., 2017. Behavioral and neural adaptations in response to five weeks of balance training in older adults: a randomized controlled trial. J Negat Results Biomed 16, 11. https://doi.org/10.1186/s12952-017-0076-1

Zuppichini, M.D., Hamlin, A.M., Zhou, Q., Kim, E., Rajagopal, S., Beltz, A.M., Polk, T.A., 2024. GABA levels decline with age: A longitudinal study. Imaging Neuroscience 2, imag–2–00224. https://doi.org/10.1162/imag_a_00224

- l.411: "Furthermore, we analysed the Pearson correlation coefficient between changes in PSQI scores and changes in SICI during sleep initiation".
What is « sleep initiation »? Weren't the participants asleep?

We thank you for highlighting this ambiguity. By sleep initiation, we refer to the transition from wakefulness to sleep. N1 is the sleep phase between being awake and in stable sleep and therefore, part of this transition.

We acknowledge that the terminology in the original manuscript was unprecise and that the terms “sleep initiation” and “while sleeping” were used inconsistently to distinguish SICI assessed during the afternoon nap from SICI assessed during the balance task. To address this, we have revised the methods section to specify the sleep stage included in the SICI analyses more clearly. In addition, all instances of “during sleep initiation” or “while falling asleep” have been replaced with either “while sleeping” or “during sleep”.

- l.414: "To evaluate whether the increase in GABA levels was associated with baseline GABA levels, we calculated the Pearson correlation coefficient between baseline GABA levels and the change in GABA levels".
What was the purpose of such analysis? How does this relate to the main hypothesis? (age, sleep, balance training)?

We thank you for this question and agree that in the original manuscript the rationale for this analysis was not sufficiently clear. This correlation analysis was not intended to test the primary hypotheses of the study, but rather to support interpretation of the regression findings in the context of the model of Taube & Lauber (2024). We have now explicitly stated in the methods and results sections that this analysis was exploratory and not part of the a priori hypothesis-driven analyses.

Methods:

[P. 17, L. 458] "Exploratory analyses

In addition to the primary hypotheses and associated analyses that were defined a priori based on previous literature and our study objectives, we performed the following additional analysis. As the effect of ΔGABA levels on ΔPSQI was negative, we wanted to test whether our data support an interpretation in the context of the model suggested by Lauber & Taube (2024). Therefore, we calculated the Pearson correlation coefficient between baseline GABA levels and the change in GABA levels."

Results:

[P. 20, L. 540] "Exploratory analyses showed that greater increases in GABA concentration after balance training were associated with lower baseline GABA levels ($r(12) = -.85$, $p < .001$)."

- Could the authors provide more details about the regression analysis, such as Durbin-Watson test (autocorrelation in the residuals), and VIF scores (absence of multicollinearity)?

Thank you for this question. We added the information about diagnostic tests in the methods and in the results section:

Methods:

[P. 16, L. 440] "Model assumptions were evaluated using the Durbin-Watson test for residual autocorrelation and variance inflation factors (VIF) to assess multicollinearity among predictors (VIF < 5). "

Results:

[P. 24, L. 618] "Diagnostic tests for the multiple linear regression model indicated no violation of model assumptions (Durbin-Watson $p = .704$; VIFs = 1.120-1.333). "

Minor remarks

- To fit the results of the current study, I would suggest changing the title of the paper, as "Improved Subjective sleep quality in older adults by enhancing the GABAergic system in the sensorimotor cortex".

We thank you for this helpful suggestion and agree that the proposed title better reflects the main findings. The title of the manuscript has been changed accordingly.

- The control group did not perform any other task and was not supervised. This might be mentioned it in the limitation section?

We thank you for this suggestion. We agree that the passive and unsupervised nature of the control group represents a limitation. This point is now explicitly acknowledged in the Limitations section of the revised manuscript (limitation 5), where we noted that the control group did not perform an alternative task and was not supervised during the intervention period. We also added an analysis of self-reported physical activity levels at PRE and POST, showing that the control group did not show evidence of changes in exercise frequency, duration, or activity type over the intervention period.

Methods:

[P. 9, L. 206] "To monitor potential baseline differences in habitual physical activity between the groups or changes in the control group throughout the study, participants reported exercise frequency (sessions per week), weekly exercise duration, and activity type(s) at PRE and POST."

[P. 16, L. 421] "We compared weekly exercise frequency and duration at baseline between balance and control group with unpaired t-tests and the changes from PRE to POST in the control group with paired t-tests"

Results:

[P. 17, L. 467] "Self-reported exercise frequency ($t(38) = 0.38$, $p = .703$, $d = 0.12$ [-0.50; 0.74]) and weekly exercise duration ($t(38) = -0.36$, $p = .719$, $d = -0.11$ [-0.73; 0.51]) did not differ between groups at baseline. Furthermore, the control group showed no change in self-reported exercise frequency from PRE to POST ($t(17) = 0.00$, $p = .999$, $d < 0.01$ [-0.46; 0.46]) and no change in weekly exercise duration ($t(17) = -0.62$, $p = .542$, $d = -0.15$ [-0.61; 0.32]). Reported types of sports also remained unchanged over time. These self-reported measures suggest that the control group maintained their habitual exercise behaviour over the intervention period, supporting comparison of the balance intervention against a stable habitual-lifestyle condition."

Discussion:

[P. 32, L. 842] "(5) Furthermore, the control group was passive and not supervised during the intervention period. However, self-reported exercise frequency, duration, and activity type remained stable from PRE to POST. Additionally, a separate strength-training comparison group did not show similar changes, suggesting that the observed effects are unlikely to be attributed to nonspecific engagement alone."

- Performing TMS during sleep poses a real challenge. Could the authors briefly explain how they ensured that the participants were still asleep throughout the TMS session?

We thank you for raising this important point. Sleep-wake state was continuously monitored using EEG, EOG, and EMG during the nap. Sleep staging was performed offline and paired-pulse TMS data were analysed after attributing them to the specific sleep phase. This clarification has now been added to the methods section of the manuscript.

[P. 13, L. 335] "SICI during sleep was assessed during an afternoon nap, which allowed controlled, feasible acquisition during the wake-to-sleep transition. For offline sleep scoring of the afternoon nap, participants were equipped with electrodes for EEG, EMG, and EOG measurements. Eight single gold cup electrodes were positioned according to the international 10-20 system (Fpz, F3, F4, Fz, M1, M2, O1, O2) (Chatrian et al., 1985). EMG and EOG electrodes were attached following the AASM recommendations (Iber et al., 2007). The recorded data were processed using BrainVision Analyzer 2.2 (Brain-Products, Ing., GmbH). EEG recordings were referenced to the contralateral mastoid and filtered according to AASM guidelines (Iber et al., 2007). TMS artifacts were removed through automatic blink detection over F3, followed by automatic ocular correction with independent component analysis and pulse artifact correction. The data were segmented from “lights off” to “lights on”. We only used the right electrodes for sleep scoring and employed the deep learning-based sleep staging algorithm U-Sleep scoring (Perslev et al., 2021) in addition to a sleep scorer who visually classified the epochs. Each 30-second epoch was classified as wake, N1 sleep, N2 sleep, N3 sleep, or rapid-eye-movement (REM) sleep. In case of disagreement between the sleep scorer and

the algorithm-based scoring, a second expert scorer was consulted. N1 sleep is the light sleep phase between being awake and in stable sleep and the MT in this stage is not yet significantly higher compared to lying awake (Avesani et al., 2008). Stimulations during light sleep (N1) were exported for SICI calculation. SICI assessment during N1 enabled us to determine the MT while the participant was lying awake and to then deliver automated stimulations at pre-calculated intensities and at a regular rhythm, thereby minimizing disturbance during the sleep opportunity. Thus, SICI measurement during N1 sleep allowed us to measure functionally relevant modulation of inhibition during sleep, without having to interrupt the rhythmic stimulations to reassess the motor threshold throughout the afternoon nap."

- l.539-540: "while the control group showed a non-significant decrease by 0.24 (SEM = 0.26) after three months of habitual living". If the difference pre-post is not significant, the term "decrease" is not appropriate. I would suggest "while the control group did not show any change after three months of habitual living (delta = 0.24, SEM = 0.26 ; (t(15) = -0.91, pbonferroni = 541 .752, d = -0.23 [-0.72; 0.27])."

We thank you for this helpful suggestion. We agree that the wording was inappropriate given the non-significant pre-post difference. The text has been revised accordingly, and "decrease" has been replaced with neutral phrasing indicating no significant change, including the full statistical information as suggested. This modification has been applied consistently throughout the manuscript.

- l.618-619: "These high baseline levels may partly explain the absence of significant changes following the balance training. ». What are the baseline levels?

We thank you for pointing out that this sentence required clarification. By "high baseline levels", we refer to both subjective quality and objective sleep architecture parameters assessed at baseline. In the revised manuscript, we now explicitly state that participants exhibited for example higher baseline sleep efficiency, shorter sleep onset latency, and greater proportion of deep sleep compared to age-matched normative values. We also clarify that these favourable baseline characteristics may have limited the potential for further improvement, thereby partly explaining the absence of significant changes following the intervention:

[P. 27, L. 681] "The good sleep quality observed in our sample of older adults was also reflected in their objective sleep architecture characteristics (supplementary table S1). For example, participants in the balance group showed higher sleep efficiency (total sleep time/total time in bed; 84.6% vs. 82%), shorter sleep onset latency (12.5 min vs. 8 min), and a greater proportion of deep sleep (12.6% vs. 9%) compared with mean values reported for the general population of around 71 years of age (Ohayon et al., 2004; Scullin & Bliwise, 2015). These high baseline levels of both subjective and objective sleep parameters may have limited the potential for further improvements, thereby partly explaining the absence of significant changes following the balance training. "

END OF COMMENTS

Dear Dr Scherrer,

Re: JP-RP-2026-290164R1 "**Improved Subjective Sleep Quality in Older Adults by Enhancing the GABAergic System in the Sensorimotor Cortex**" by Selin Scherrer, Sven Egger, Xinyu Liu, Anna Zoé Wick, Björn Rasch, Lijing Xin, Benedikt Lauber, and Wolfgang Taube

Thank you for submitting your manuscript to The Journal of Physiology. It has been assessed by a Reviewing Editor and by 2 expert referees and we are pleased to tell you that it is acceptable for publication following satisfactory revision.

The referee reports are copied at the end of this email.

Please address all the points raised and incorporate all requested revisions or explain in your Response to Referees why a change has not been made. We hope you will find the comments helpful and that you will be able to return your revised manuscript within 4 weeks. If you require longer than this, please contact journal staff: jp@physoc.org.

Your revised manuscript should be submitted online using the link in your Author Tasks Link Not Available. This link is accessible via your account as Corresponding Author; it is not available to your co-authors. If this presents a problem, please contact journal staff (jp@physoc.org). Image files from the previous version are retained on the system. Please ensure you replace or remove any files that are being revised.

If you do not wish to submit a revised version of your manuscript, you must inform our journal staff (jp@physoc.org) or reply to this email to request withdrawal. Please note that a manuscript must be formally withdrawn from the peer review process at one journal before it may be submitted to another journal.

TRANSPARENT PEER REVIEW POLICY: To improve the transparency of its peer review process The Journal of Physiology publishes online, as supporting information, the peer review history of all articles accepted for publication. Readers will have access to decision letters, including Editors' comments and referee reports, for each version of the manuscript, as well as any author responses to peer review comments. Referees can decide whether or not they wish to be named on the peer review history document.

ABSTRACT FIGURES: Authors are expected to use The Journal's premium BioRender account to create/redraw their Abstract Figures. Information on how to access this account is here: https://physoc.onlinelibrary.wiley.com/journal/14697793/biorender-access.

This will enable Authors to create and download high-resolution figures. If authors have used the free BioRender service, they can use the instructions provided in the link above to download a high-resolution version suitable for publication.

The link provided should only be used for the purposes of this submission. Authors will be charged for figures created on this account if they are not related to this manuscript submission.

LANGUAGE EDITING AND SUPPORT FOR PUBLICATION: If you would like help with English language editing, or other article preparation support, Wiley Editing Services offers expert help, including English Language Editing, as well as translation, manuscript formatting, and figure formatting at www.wileyauthors.com/eeo/preparation. You can also find resources for Preparing Your Article for general guidance about writing and preparing your manuscript at www.wileyauthors.com/eeo/prepresources.

RIGOUR AND REPRODUCIBILITY: We recommend authors consult The Journal's Rigour and Reproducibility requirements to ensure that their manuscript is suitable for publication: https://physoc.onlinelibrary.wiley.com/pb-assets/hub-assets/physoc/documents/TJP-Rigour-and-Reproducibility-Requirements.pdf

REVISION CHECKLIST:

Check that your Methods section conforms to journal policy: https://jp.msubmit.net/cgi-bin/main.plex?form_type=display_requirements#methods

Check that data presented conforms to the statistics policy: https://jp.msubmit.net/cgi-bin/main.plex?form_type=display_requirements#statistics

Upload a full Response to Referees file. To create your 'Response to Referees' copy all the reports, including any comments from the Senior and Reviewing Editors, into a Microsoft Word, or similar, file and respond to each point, using font or background colour to distinguish comments and responses and upload as the required file type.

Please upload two versions of your manuscript text: one with all relevant changes highlighted and one clean version with no changes tracked. The manuscript file should include all tables and figure legends, but each figure/graph should be uploaded as separate, high-resolution files. The journal is now integrated with Wiley's Image Checking service. For further details, see: https://www.wiley.com/en-us/network/publishing/research-publishing/trending-stories/upholding-image-integrity-wileys-

image-screening-service

You may also upload:

- 'Potential Cover Art' for consideration as the issue's cover image
- Appropriate Supporting Information (Video, audio or data set: see https://jp.msubmit.net/cgi-bin/main.plex?form_type=display_requirements#supp).

We look forward to receiving your revised submission.

If you have any queries, please reply to this email and we will be pleased to advise.

Yours sincerely,

Richard Carson
Senior Editor
The Journal of Physiology

REQUIRED ITEMS

New:
- Authors are required to fully disclose the use of any Generative AI (GAI) tools used in the preparation of their manuscript. This disclosure should also be included in the 'Methods' section (or a suitable location for article types not requiring Methods), and include the name(s), model and version of AI technology used, and exact details on the purpose for, and methods of, its use.

If no AI tool has been used, this should also be specifically stated within the manuscript.

EDITOR COMMENTS

Reviewing Editor:
The reviewers are satisfied that their concerns have been addressed

Senior Editor:
Please note that the Journal does not typically publish Supplementary Materials.

See:

https://jp.msubmit.net/cgi-bin/main.plex?form_type=display_requirements#suppinfo

Specifically:

"The Journal of Physiology encourages authors to include as much relevant material as possible within each published article. Figures, tables, text (e.g. expanded/detailed methods or results), equations, and other material, if essential for the complete understanding of the manuscript and can fit on a printed page or pages, must be incorporated into the manuscript itself as part of the text or as standard figures or tables, and not supplied as supporting information.

If inclusion of this material would interrupt the 'flow' of the paper, authors are welcome to include this additional material as an Appendix at the end of the article (rather than as separate supporting information). An Appendix should be included within the article file of a revised version; any figures and tables should be numbered as Fig. A1, Fig. A2, Table A1, etc. - and each should be cited in the text".

I therefore recommend that the authors include the content of both Supplementary Information - Methods and Supplementary Information - Results (and Bibliography Supplementary Information) within the main body of the manuscript.

The "Detailed balance training plan" can be included in an appendix.

REFEREE COMMENTS

Referee #1:

Points raised in the original review have been satisfactorily addressed.

Referee #2:

The authors appropriately responded to my comments.

I have no further remark.

END OF COMMENTS

REQUIRED ITEMS

New:
- Authors are required to [<a]https://jp.msubmit.net/cgi-bin/main.plex?form_type=display_requirements#aigcfully disclose the use of any Generative AI (GAI) tools used in the preparation of their manuscript. This disclosure should also be included in the 'Methods' section (or a suitable location for article types not requiring Methods), and include the name(s), model and version of AI technology used, and exact details on the purpose for, and methods of, its use.

If no AI tool has been used, this should also be specifically stated within the manuscript.

We have added the following paragraph at the end of the Methods section (page and line numbers correspond to the clean manuscript version):

[p. 18, l. 515] **Generative Artificial Intelligence (GAI)**

Microsoft 365 Copilot (GPT-5 chat model) was used to support code debugging during statistical analyses and to assist in refining the existing manuscript by improving flow, readability, conciseness, and language quality. No original content was generated by GAI and all GAI-assisted text was reviewed and revised by the authors to ensure accuracy and clarity.

EDITOR COMMENTS

Reviewing Editor:
The reviewers are satisfied that their concerns have been addressed

Senior Editor:
Please note that the Journal does not typically publish Supplementary Materials.

See:

https://jp.msubmit.net/cgi-bin/main.plex?form_type=display_requirements#suppinfo

Specifically:

"The Journal of Physiology encourages authors to include as much relevant material as possible within each published article. Figures, tables, text (e.g. expanded/detailed methods or results), equations, and other material, if essential for the complete understanding of the manuscript and can fit on a printed page or pages, must be incorporated into the manuscript itself as part of the text or as standard figures or tables, and not supplied as supporting information.

If inclusion of this material would interrupt the 'flow' of the paper, authors are welcome to include this additional material as an Appendix at the end of the article (rather than as separate supporting information). An Appendix should be included within the article file of a revised version; any figures and tables should be numbered as Fig. A1, Fig. A2, Table A1, etc. - and each should be cited in the text".

I therefore recommend that the authors include the content of both Supplementary Information - Methods and Supplementary Information - Results (and Bibliography Supplementary Information) within the main body of the manuscript.

The "Detailed balance training plan" can be included in an appendix.

We thank the editor for highlighting this journal policy. In response, we have added the supplementary tables to the main manuscript and the following text to the Methods and Results section:

Methods section:

[p. 17, l. 465] **Training specificity – Resistance Training Control Group**

The primary comparisons relevant to the hypotheses of this study focused on the BAL and CON group. We added a resistance training group (RT) which served as an active control to assess specificity of balance training effects relative to general physical activity.

Participants

Twenty volunteers aged 65 to 79 years were recruited through newspaper advertisements and an information session after first mention of interest. All participants were free of any known neurological and orthopaedic diseases or injuries and contraindications to MRI and TMS measurements. Participants received appropriate compensation in accordance with the guidelines of the ethics commission. Two participants dropped out before POST tests due to detected diseases during the study intervention. Consequently, 18 participants (7 females, 70.11 ± 4.00 years old) were included in the analysis.

Resistance training intervention

Similar to the balance training intervention, the resistance training was conducted in groups. Participants attended supervised sessions three times a week, totalling at least 30 training sessions of 45 minutes each. Each session included a warm-up, the main part, and a cool-down. The warm-up and cool-down together taking approximately 15 minutes and the main part 30 minutes. The main part of the session consisted of four exercises targeting the lower extremities. Each session included one exercise for ankle dorsiflexion, ankle plantarflexion, knee flexion, and knee extension. The intensity of the resistance training progressively increased through adjustments in the number of repetitions, sets, and external load, as well as the inclusion of explosive exercises to provide optimal stimuli (Fragala *et al.*, 2019). The most relevant exercise for this study was ankle dorsiflexion, while the other exercises were added to make the training more beneficial and attractive for the subjects. Ankle dorsiflexion was trained against the resistance of elastic bands. Participants engaged in three weeks of traditional training, during which the number of sets increased from one to three sets of 10-15 repetitions and the resistance band strength was increased. Over the following nine-week period, the training program comprised alternating sessions of explosive training (2-3x 6-10 repetitions, contraction as fast as possible – hold shortly – slow excentric movement back) and traditional training (2-3x 8-12 repetitions, slow concentric and excentric movement). The rest interval between sets was

1-2 minutes. The participants added weight cuffs on their foot, increasing the weight by 0.5 kg every week. The resistance was reduced by 1 kg for the explosive executions.

Explosive strength performance assessment

The force assessment was carried out in seated position on an isokinetic machine (Cybex Norm, HUMAC, CA, USA). The right leg of the participant was placed on the foot pedal of the machine. To achieve the most valid power transmission possible, the participants were equipped with customized shoes directly attached to the foot pedal, additionally attached with Velcro straps. Participants executed isometric ankle dorsiflexion in a neutral zero position and were verbally encouraged to maximize peak rate of torque development (RTD) by fast dorsiflexion. The participants performed two familiarization trials prior to the measurement, which consisted of five trials with at least 15 seconds of rest between each trial (Maffiuletti *et al.*, 2016). The peak RTD was identified as the steepest point of the RTD force curve using a 20 ms moving window. Valid trials were averaged for pre to post comparison. Trials failing to meet requirements (i.e. countermovement or upper body movements) were excluded from the analysis. We had to delete data of four participants, because the participants changed their execution strategy from PRE to POST. Furthermore, we had to delete three trials from two different participants, all other individuals had five valid trials.

Statistical analyses

We compared parameters assessed at baseline and post-intervention with paired t-tests.

Results section:

[p. 22, l. 627] **Training Specificity – Resistance Training Control Group**

The RT group showed increased explosive strength after training, while the GABAergic system and subjective sleep quality remained unchanged (Table 2), indicating that these effects were specific to balance training rather than general physical activity.

REFEREE COMMENTS

Referee #1:

Points raised in the original review have been satisfactorily addressed.

Thank you for the positive feedback.

Referee #2:

The authors appropriately responded to my comments.

I have no further remark.

Thank you for the positive feedback.

END OF COMMENTS

Dear Dr Scherrer,

Re: JP-RP-2026-290164R2 "**Improved Subjective Sleep Quality in Older Adults by Enhancing the GABAergic System in the Sensorimotor Cortex**" by Selin Scherrer, Sven Egger, Xinyu Liu, Anna Zoé Wick, Björn Rasch, Lijing Xin, Benedikt Lauber, and Wolfgang Taube

Thank you for submitting your manuscript to The Journal of Physiology. It has been assessed by a Reviewing Editor and by 0 expert referees and we are pleased to tell you that it is acceptable for publication following satisfactory revision.

The referee reports are copied at the end of this email.

Please address all the points raised and incorporate all requested revisions or explain in your Response to Referees why a change has not been made. We hope you will find the comments helpful and that you will be able to return your revised manuscript within 4 weeks. If you require longer than this, please contact journal staff: jp@physoc.org.

Your revised manuscript should be submitted online using the link in your Author Tasks Link Not Available. This link is accessible via your account as Corresponding Author; it is not available to your co-authors. If this presents a problem, please contact journal staff (jp@physoc.org). Image files from the previous version are retained on the system. Please ensure you replace or remove any files that are being revised.

If you do not wish to submit a revised version of your manuscript, you must inform our journal staff (jp@physoc.org) or reply to this email to request withdrawal. Please note that a manuscript must be formally withdrawn from the peer review process at one journal before it may be submitted to another journal.

TRANSPARENT PEER REVIEW POLICY: To improve the transparency of its peer review process The Journal of Physiology publishes online, as supporting information, the peer review history of all articles accepted for publication. Readers will have access to decision letters, including Editors' comments and referee reports, for each version of the manuscript, as well as any author responses to peer review comments. Referees can decide whether or not they wish to be named on the peer review history document.

ABSTRACT FIGURES: Authors are expected to use The Journal's premium BioRender account to create/redraw their Abstract Figures. Information on how to access this account is here: https://physoc.onlinelibrary.wiley.com/journal/14697793/biorender-access.

This will enable Authors to create and download high-resolution figures. If authors have used the free BioRender service, they can use the instructions provided in the link above to download a high-resolution version suitable for publication.

The link provided should only be used for the purposes of this submission. Authors will be charged for figures created on this account if they are not related to this manuscript submission.

LANGUAGE EDITING AND SUPPORT FOR PUBLICATION: If you would like help with English language editing, or other article preparation support, Wiley Editing Services offers expert help, including English Language Editing, as well as translation, manuscript formatting, and figure formatting at www.wileyauthors.com/eeo/preparation. You can also find resources for Preparing Your Article for general guidance about writing and preparing your manuscript at www.wileyauthors.com/eeo/prepresources.

RIGOUR AND REPRODUCIBILITY: We recommend authors consult The Journal's Rigour and Reproducibility requirements to ensure that their manuscript is suitable for publication: https://physoc.onlinelibrary.wiley.com/pb-assets/hub-assets/physoc/documents/TJP-Rigour-and-Reproducibility-Requirements.pdf

REVISION CHECKLIST:

Check that your Methods section conforms to journal policy: https://jp.msubmit.net/cgi-bin/main.plex?form_type=display_requirements#methods

Check that data presented conforms to the statistics policy: https://jp.msubmit.net/cgi-bin/main.plex?form_type=display_requirements#statistics

Upload a full Response to Referees file. To create your 'Response to Referees' copy all the reports, including any comments from the Senior and Reviewing Editors, into a Microsoft Word, or similar, file and respond to each point, using font or background colour to distinguish comments and responses and upload as the required file type.

Please upload two versions of your manuscript text: one with all relevant changes highlighted and one clean version with no changes tracked. The manuscript file should include all tables and figure legends, but each figure/graph should be uploaded as separate, high-resolution files. The journal is now integrated with Wiley's Image Checking service. For further details, see: https://www.wiley.com/en-us/network/publishing/research-publishing/trending-stories/upholding-image-integrity-wileys-

image-screening-service

You may also upload:

- 'Potential Cover Art' for consideration as the issue's cover image
- Appropriate Supporting Information (Video, audio or data set: see https://jp.msubmit.net/cgi-bin/main.plex?form_type=display_requirements#supp).

We look forward to receiving your revised submission.

If you have any queries, please reply to this email and we will be pleased to advise.

Yours sincerely,

Richard Carson
Senior Editor
The Journal of Physiology

EDITOR COMMENTS

Senior Editor:

Please provide a version of the most recent submission in which the changes have been highlighted

The current "changes highlighted" file (submitted 03/06/2026) does not match the file without highlighting.

END OF COMMENTS

EDITOR COMMENTS

Senior Editor:

Please provide a version of the most recent submission in which the changes have been highlighted

The current "changes highlighted" file (submitted 03/06/2026) does not match the file without highlighting.

Thank you for highlighting this discrepancy. We apologize for the mismatch between the tracked-changes file and the clean version in our previous submission.

We have now uploaded a corrected highlighted version that accurately reflects all revisions made to the clean manuscript.

Please let us know if anything further is needed.

____

END OF COMMENTS

Dear Ms Scherrer,

Re: JP-RP-2026-290164R3 "**Improved Subjective Sleep Quality in Older Adults by Enhancing the GABAergic System in the Sensorimotor Cortex**" by Selin Scherrer, Sven Egger, Xinyu Liu, Anna Zoé Wick, Björn Rasch, Lijing Xin, Benedikt Lauber, and Wolfgang Taube

We are pleased to tell you that your paper has been accepted for publication in The Journal of Physiology.

The last Word (or similar) version of the manuscript provided will be used by the Production Editor to prepare your proof. When this is ready you will receive an email containing a link to Wiley's Online Proofing System. The proof should be thoroughly checked and corrected as promptly as possible.

Authors should note that it is too late at this point to offer corrections prior to proofreading. Major corrections at proof stage, such as changes to figures, will be referred to the Editors for approval before they can be incorporated. Only minor changes, such as to style and consistency, should be made at proof stage. Changes that need to be made after proof stage will usually require a formal correction notice.

All queries at proof stage should be sent to: TJP@wiley.com.

Following copyediting and typesetting the accepted version will be published online.

Yours sincerely,

Richard Carson
Senior Editor
The Journal of Physiology

IMPORTANT POINTS TO NOTE FOLLOWING ACCEPTANCE OF YOUR PAPER:

• IMPORTANT NOTICE ABOUT OPEN ACCESS: To assist authors whose funding agencies mandate immediate public access to published research findings, The Journal of Physiology allows authors to pay an Open Access (OA) fee to have their papers made freely available immediately on publication.

The Corresponding Author will receive an email from Wiley with details on how to register or log in to Wiley Authors where you will be able to place an order.

• You can check if your funder or institution has a Wiley Open Access Account here:
https://authors.wiley.com/author-resources/Journal-Authors/open-access/author-compliance-tool.html

• TRANSPARENT PEER REVIEW POLICY: To improve the transparency of its peer review process, The Journal of Physiology publishes online as supporting information the peer review history of all articles accepted for publication. Readers will have access to decision letters, including Editors' comments and referee reports, for each version of the manuscript as well as any author responses to peer review comments. Referees can decide whether or not they wish to be named on the peer review history document.

• You can help your research get the attention it deserves! Check out Wiley's free Promotion Guide for best-practice recommendations for promoting your work at: www.wileyauthors.com/eeo/guide. You can learn more about Wiley Editing Services which offers professional video, design, and writing services to create shareable video abstracts, infographics, conference posters, lay summaries, and research news stories for your research at: www.wileyauthors.com/eeo/promotion.

• If you would like to receive our 'Research Roundup', a monthly newsletter highlighting the cutting-edge research published in The Physiological Society's family of journals (The Journal of Physiology, Experimental Physiology, Physiological Reports, The Journal of Nutritional Physiology and The Journal of Precision Medicine: Health and Disease), please click this link, fill in your name and email address and select 'Research Roundup':
https://www.physoc.org/journals-and-media/membernews
